# Formation of Irida-β-ketoimines and PCN^amine^-Ir(III) Complexes by Reacting Irida-β-diketones with Aliphatic Diamines: Catalytic Activity in Hydrogen Release by Methanolysis of H_3_N–BH_3_

**DOI:** 10.1021/acs.organomet.2c00451

**Published:** 2022-12-01

**Authors:** Itxaso Bustos, Jose M. Seco, Antonio Rodriguez-Dieguez, María A. Garralda, Claudio Mendicute-Fierro

**Affiliations:** † Department of Applied Chemistry, Faculty of Chemistry, University of The Basque Country UPV/EHU, Paseo Manuel Lardizabal 3, 20018Donostia-San Sebastián, Spain; ‡ Department of Inorganic Chemistry, Faculty of Science, University of Granada, 18071Granada, Spain

## Abstract

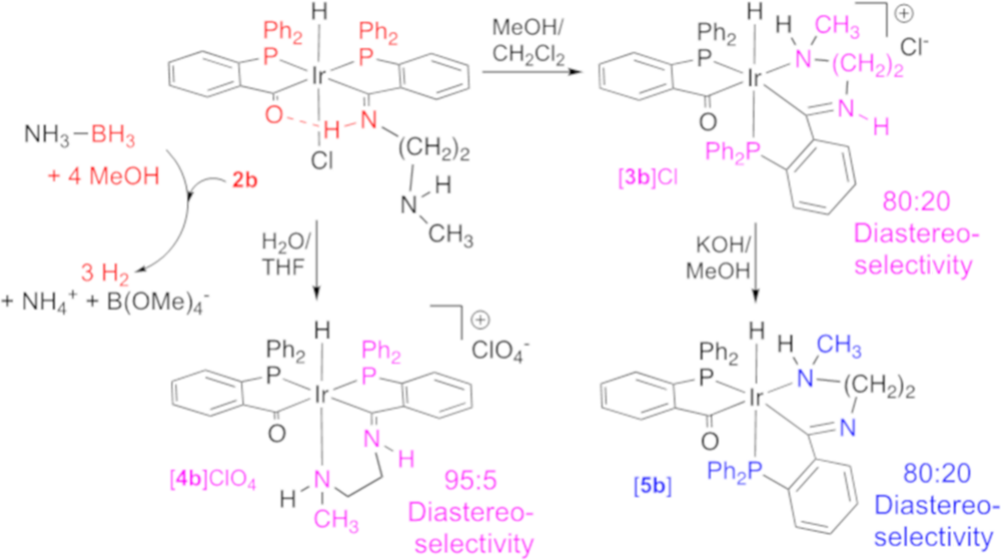

Aliphatic diamines [(H_2_N(CH_2_)_*n*_NHR) (**a–d**) *n* = 2: R = H (**a**), R = CH_3_ (**b**), R = C_2_H_5_ (**c**), *n* = 3, R = H (**d**) or *rac*-2-(aminomethyl)piperidine (**e**)] react with [IrH(Cl){(PPh_2_(*o*-C_6_H_4_CO))_2_H}] in THF to afford ketoimine complexes [IrH(Cl){(PPh_2_(*o*-C_6_H_4_CO))(PPh_2_(*o*-C_6_H_4_CN(CH_2_)_*n*_NHR))H}] (**2a**–**2d**) or [IrH(Cl){(PPh_2_(*o*-C_6_H_4_CO))(PPh_2_(*o*-C_6_H_4_CNCH_2_(C_5_H_9_NH)))H}] (**2e**), containing a bridging N–H···O hydrogen bond and a dangling amine. Complex **2e** consists of an almost equimolar mixture of two diastereomers. In protic solvents, the dangling amine in complexes **2** displaces chloride to afford cationic acyl-iminium compounds, [IrH(PPh_2_(*o*-C_6_H_4_CO))(PPh_2_(*o*-C_6_H_4_CNH(CH_2_)_*n*_NHR))]X (**3a**–**3d**, X = Cl) or [IrH(PPh_2_(*o*-C_6_H_4_CO))(PPh_2_(*o*-C_6_H_4_CNHCH_2_(C_5_H_9_NH)))]Cl (**3e**) and (**4a**–**4b**, X = ClO_4_), with new hemilabile terdentate PCN^amine^ ligands adopting a facial disposition. Complexes **3** contain the corresponding phosphorus atom trans to hydride and the amine fragment trans to acyl, while complexes **4** contain the amine *trans* to hydride. **3b** and **4b** consist of 80:20 and 95:5 mixtures of diastereomers, respectively, while **3e** contains a 65:35 mixture. In the presence of KOH, intermediate cationic acyl-iminium complexes **3** transform into neutral acyl-imine [IrH(PPh_2_(*o*-C_6_H_4_CO))(PPh_2_(*o*-C_6_H_4_CN(CH_2_)_*n*_NHR))] derivatives (**5**) with retention of the stereochemistry. Single-crystal X-ray diffraction analysis was performed on **2a**, [**3a**]Cl, [**3b**]Cl, [**4a**]ClO_4_, and **5b**. Complexes **2**, **3**, and **5** catalyze the methanolysis of ammonia-borane under air to release hydrogen. The highest activity is observed for ketoimine complexes **2**.

## Introduction

**Figure 1:**
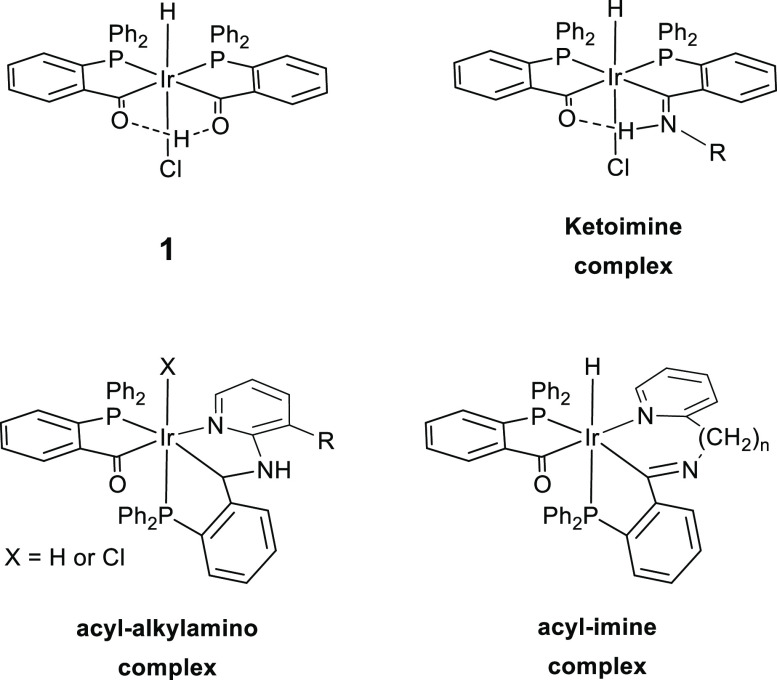
Irida-β-diketone **1**, irida-β-ketoimine, and the reaction products of **1** with aminopyridines.

Metalla-β-diketones can be considered as containing hydroxycarbene and acyl functionalities connected by an intramolecular hydrogen bond,^1^ which is reported to be stronger than that in acetylacetone^2^ and stabilizes the hydroxycarbene moiety. These complexes can be easily deprotonated and may behave as metalla-β-diketonate ligands toward main-group and transition metals. Nitrogen-containing nucleophiles typically attack the carbene carbon atom to afford metalla-β-ketoimines. In 2010, our group disclosed the hydridoirida-β-diketone **1** (see Figure 1) as the first homogeneous catalyst for the hydrolysis of ammonia-borane (H_3_N–BH_3_, AB) to release hydrogen.^3^ Release of H_2_, a sustainable energy source, from chemical hydrides such as AB is being intensively studied^4^ and includes, among others, this hydrolytic procedure.^5^ Efficient homogeneous hydrolysis catalysts based on Ir,^6^ Ru,^7^ or Rh^8^ have been reported. It has been reported that amino-complexes can catalyze the H_2_ release in these reactions;^9,10^ therefore, we have investigated the reactivity of **1** toward various nitrogen-containing compounds, such as ammonia,^9^ alkyl and aromatic monoamines,^9,11^ amines connected to pyridine functionalities,^12,13^ and furfurylamine.^14^

In general terms, these reactions have yielded several types of compounds depending on the reaction conditions. Aliphatic monoamines afford two different types of products, the condensation product, a ketoimine complex, see Figure 1, and a dehydrodechlorination product [IrH(PPh_2_(*o*-C_6_H_4_CO))_2_(amine)] with coordinated amine trans to phosphorus, while aromatic amines gave cationic hydridoirida-β-diketones [IrH{(PPh_2_(*o*-C_6_H_4_CO))_2_H}(amine)]^+^, with amine *trans* to hydride. Aminopyridines gave, in addition to ketoimine- and amine-type complexes, acyl-alkylamines or acyl-imines, which upon coordination of pyridine afforded Ir(III) complexes with PCN^py^-coordinated ligands, see Figure 1. This prompted a rearrangement of the ligands and placed the pyridinic atom of the newly formed PCN ligand *trans* to the acyl group. The intended use of these complexes in the hydrolysis of ammonia borane was marred by their lack of solubility in water or water/tetrahydrofuran mixtures.

As a result, we initiated the study of the methanolysis of ammonia borane, for which an advantageous regeneration method of ammonia-borane is available.^15^ Following our leading article on the homogeneous ruthenium-catalyzed solvolysis of H_3_N–BH_3_,^16^ we revisited compound **1** as the catalyst in the methanolysis of AB, which, despite its lack of solubility in methanol, liberates hydrogen in an efficient and fast homogeneous fashion.^17^ However, this is not the case for any of the aforementioned monoamine derivatives or PCN^py^ complexes derived from aminopyridines thus far studied.

Pincer complexes have been widely used in organometallic chemistry on account of their stability and stereoelectronic tunability. The unsymmetric version affords a potentially hemilabile environment around the metal center that may be relevant to the reactivity and catalytic activity of the complexes.^18^ We found it interesting to study the reaction of complex **1** with aliphatic diamines that could afford coordinated PCN^amine^ ligands with hard N and soft P donors, of very different *trans* influences, occupying the arms of the ligand and allowing us to study the influence of the length of the spacer connecting both N-functionalities.^19^ PCN^amine^ complexes have been reported as more reactive than related PCP complexes.^20^ An Ir^III^ (PCN^pyrazole^)HCl complex proves more efficient than the symmetrical (PCP)IrHCl as the AB dehydrogenation catalyst though less efficient than related (POCOP)IrH_2_.^21^

**Figure 2:**
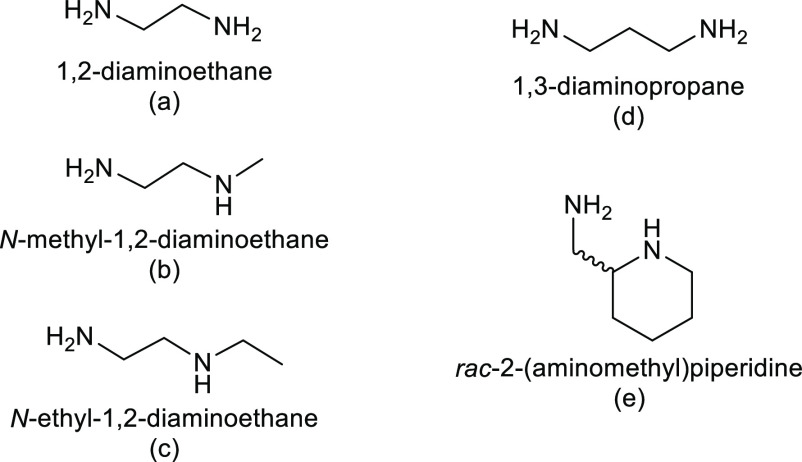
Diamines used in this work.

Here, we present the reaction of **1** with different alkyl diamines, see Figure 2, both primary (1,2-diaminoethane, **a**, and 1,3-diaminopropane, **d**) and mixed primary/secondary amines (*N*-methyl-1,2-diaminoethane, **b**; N-ethyl-1,2-diaminoethane, **c**; and racemic 2-(aminomethyl)piperidine, **e**). Worth noting is that secondary amines generate a stereogenic center upon coordination, and when they bind a prochiral or even racemic metal center, they can give rise to high diastereoselective products.^22^ These ligands yield two new types of structures, elusive intermediate acyl-iminium compounds and acyl-imines in PCN^amine^ complexes, which have been isolated and characterized using spectroscopic techniques and single-crystal X-ray diffraction. In addition, their capability in the methanolysis of ammonia borane has been tested.

## Results and Discussion

### Ketoimine-Type Complex Formation (**2**)

**Scheme 1:**
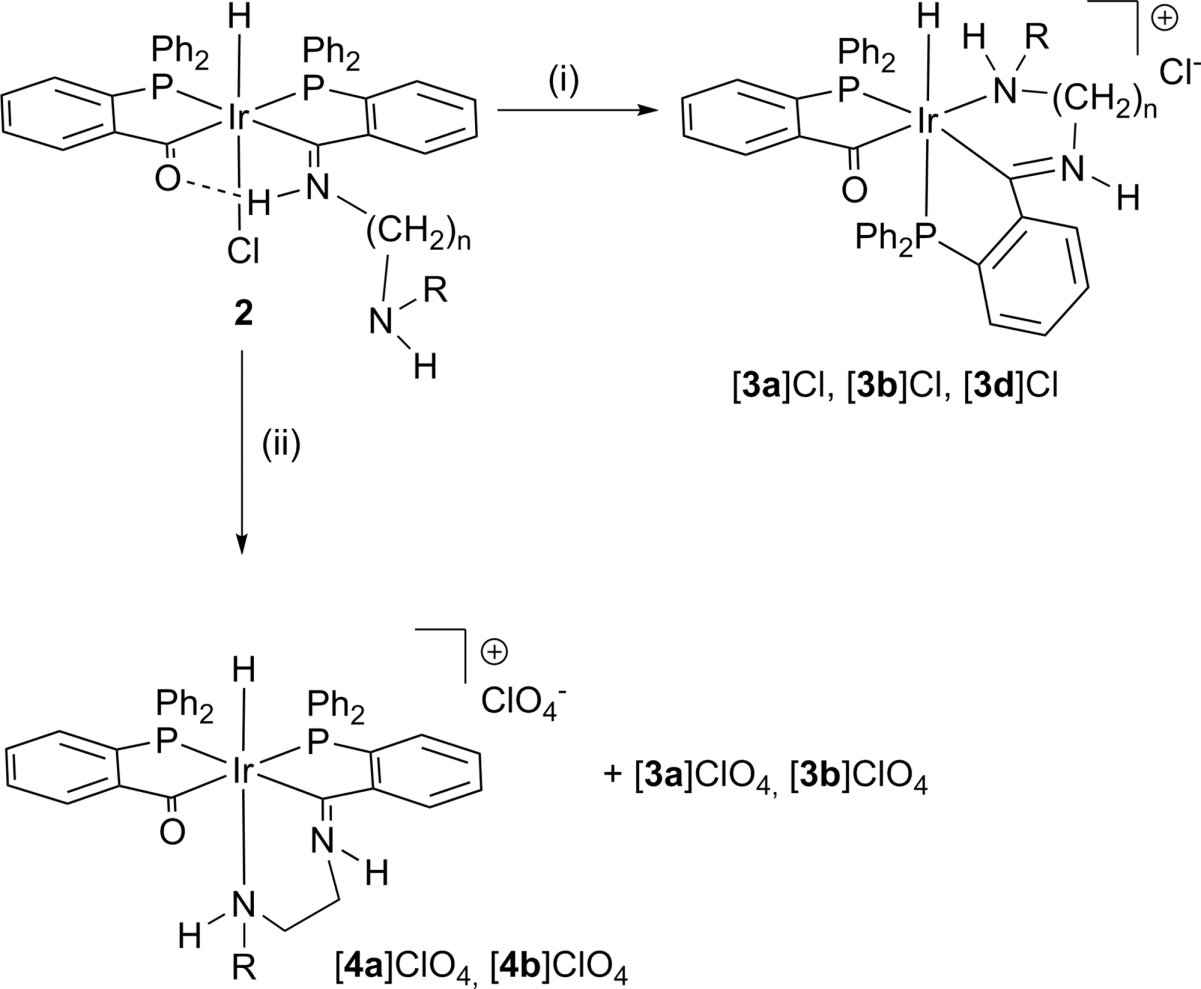
Formation of Cationic Acyl-Iminium Complexes (i) In MeOH/CH_2_Cl_2_ (24 h), only [**3**]Cl compounds. (ii) In THF/H_2_O (24 h) and addition of NaClO_4_. [**4a**]ClO_4_/[**3a**]ClO_4_ = 80:20 and [**4b**]ClO_4_/[**3b**]ClO_4_ = 60:40 mixtures. *n* = 2: R = H (**a**), R = CH_3_ (**b**), *n* = 3, R = H (**d**).

**Scheme 2:**
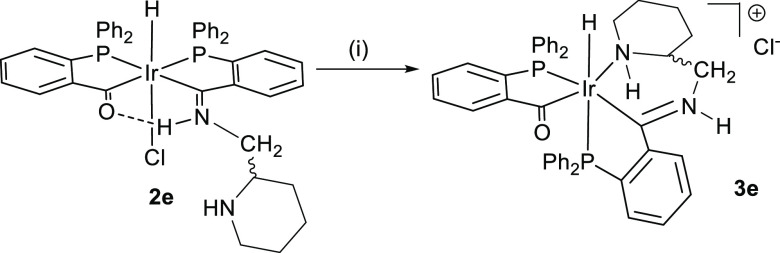
Formation of the Cationic Acyl-Iminium Complex with *rac*-2-(Aminoethyl)pyridine and the Generation of **3** Stereogenic Centers (i) In MeOH/CH_2_Cl_2_.

The reaction of **1** with the different alkyl diamines (H_2_N(CH_2_)_*n*_NHR) in THF leads to the formation of ketoimine-type complexes (see Figure 1). In all cases, complexes [IrH(Cl){(PPh_2_(*o*-C_6_H_4_CO))(PPh_2_(*o*-C_6_H_4_CN(CH_2_)_*n*_NHR))H}] (**2a**–**2d**) in Scheme 1 or [IrH(Cl){(PPh_2_(*o*-C_6_H_4_CO))(PPh_2_(*o*-C_6_H_4_CNCH_2_(C_5_H_9_NH)))H}] (**2e**) in Scheme 2, with a dangling amine, were obtained. In these complexes, the condensation reaction of the primary amine leaves the coordination environment of **1** unchanged, and the initial ketoenolic proton is also located between two heteroatoms, in this case nitrogen and oxygen. Another different structural parameter in **2** is the presence of a variable group on the dangling nitrogen atom.

In the IR spectra, see Experimental Section for details, the formation of the compounds can be ascertained by the presence of signals due to the N–H bonds (ca. 3280 cm^–1^) and the maintenance of the Ir–H bond signals (ca. 2180 cm^–1^). However, only one signal for the C=N and C=O bonds can be observed (ca. 1555 cm^–1^). All complexes have also been characterized by multinuclear NMR spectroscopy. The most characteristic signals in the ^1^H NMR spectra of these hydrido-ketoimine complexes are the resonance at ca. 13 ppm, corresponding to the bridging O···H–N proton, and the signal at ca. −20.5 ppm, assigned to a hydride *trans* to a chloride ligand. In all complexes, the latter appears as a triplet, except for complex **2e**, derived from racemic 2-(aminomethyl)piperidine, where two doublets of doublets are observed, due to the expected two diastereomers, which are isolated in the ca. 55:45 ratio.

The ^31^P{^1^H} spectra of **2a**–**2d** show two doublets in the 14–17 and 28–30 ppm ranges with a coupling constant of 7 Hz, which indicate two phosphorus atoms in the relative *cis* position. For complex **2e**, however, the high field signal for each diastereomer appears as a broad singlet due to unavoidable proton-phosphorus incomplete decoupling. The most characteristic signals in the ^13^C{^1^H} spectra are those for the acyl and iminoacyl groups at 224 and 242 ppm, which appear as doublets, with a coupling constant of 102–106 Hz indicating coupling with *trans* phosphorus atoms.

**Figure 3:**
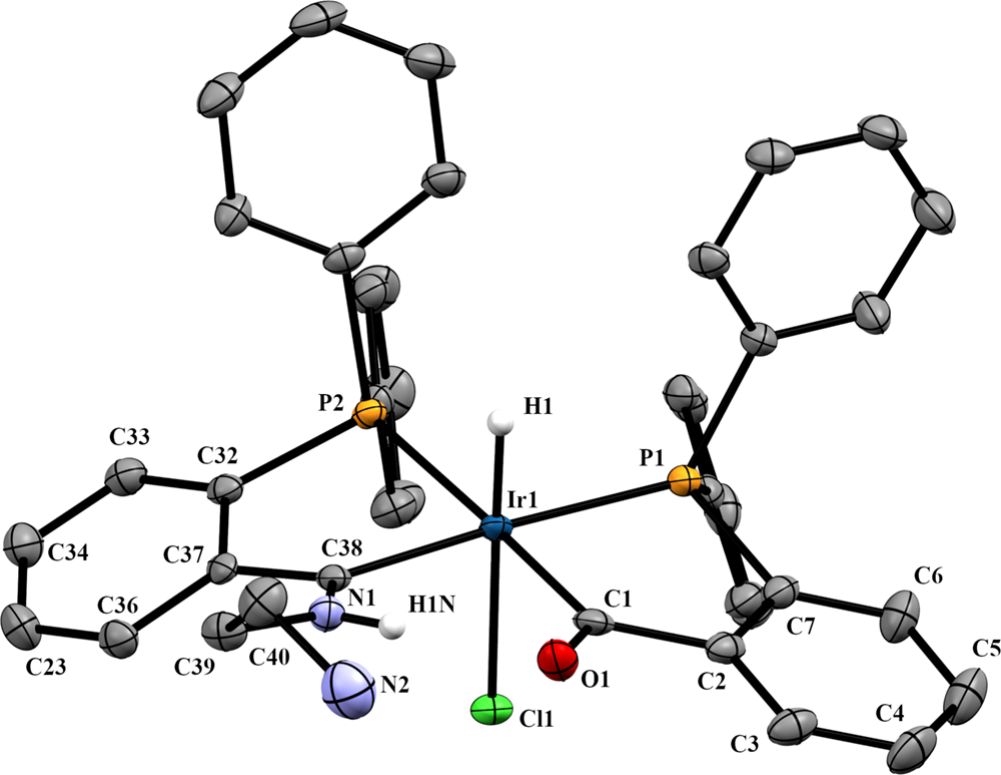
Molecular structure of **2a**, *OC*-6-65-*C* isomer. Displacement ellipsoids are drawn at the 50% probability level. Most of hydrogen atoms and crystallized methanol molecules have been omitted for clarity. Selected bond lengths (Å) and angles (°): Ir1–P1, 2.3000(7); Ir1–P2, 2.3361(8); Ir1–C1, 2.0486(7); Ir1–C38, 2.0625(7); Ir1–Cl1, 2.4919(7); Ir1–H1, 1.4844(4); C38–N1, 1.2994(3); P1–Ir1–C1, 82.9(1); P2–Ir1–C38, 81.5(1); P2–Ir1–C1, 173.0(1); P1–Ir1–C38, 175.9(1); C2–C1–O1, 115.0(3); C37–C38–N1, 119.8(3).

Crystals of **2a** consist of a racemate of the proposed structure with a distorted octahedral geometry, see Figure 3, where all bond distances and angles are very similar to those in analogous reported structures derived from methylamine,^3^ 2-(aminoethyl)pyridine,^13^ and furfurylamine.^14^

### Acyl-Iminium-Type Complex Formation (**3** and **4**)

In protic solvents, ketoimine-type complexes **2**, containing a dangling amine, undergo amine coordination with the displacement of chloride. This rearrangement leads to the cleavage of the O···H–N hydrogen bond in **2**, to give acyl-iminium-type compounds, with terdentate PCN^amine^ ligands, as shown in Schemes 1 and 2. This type of cationic complexes were proposed as intermediates in the formation of PCN^py^ terdentate ligands, containing acyl-imine moieties in neutral complexes, when using 2-(aminoalkyl)pyridines^13^ though they could be neither isolated nor detected. By using aliphatic diamines, we have succeeded in the isolation of different isomers shown in Scheme 1 depending on the employed solvent.

When using MeOH/CH_2_Cl_2_ mixtures (Scheme 1i), ketoimine complexes **2a–2d** undergo slow amine coordination and also slow isomerization with loss of the coplanarity of the CPPC fragment, frequent upon hydrogen bond cleavage in the related quasi-tetradentate ligand-containing complexes,^11^ to afford cationic complexes [**3a**]Cl**-**[**3d**]Cl after 24 h. In pure MeOH, the reaction is still slower and fails to reach completion. In the IR spectra, the vibrations of the N–H bonds remain almost unaltered; however, there is a dramatic change in the ν(Ir–H) stretching shifting to ca. 2050 cm^–1^, reflecting the different *trans* character of the chloride and phosphane ligands. No appreciable change in the vibrations of the C=N or C=O bonds is observed.

The multinuclear NMR spectroscopy study of the complexes also shows the changes around the metal center. As in the IR spectra, the largest change is observed for the hydride signal in the ^1^H NMR, which now appears at ca. −8.7 ppm as a doublet of doublets. This is assigned to coupling with a *trans* phosphorus atom, with a coupling constant of ca. 123 Hz, and coupling with a *cis* phosphorus atom, with a coupling constant of ca. 19 Hz. The signal for the iminium proton, only observed in **3d**, is slightly displaced toward a higher field. Interestingly, upon coordination of the secondary amine −NHMe moiety in complex **3b**, a new stereogenic center is created, which results in **3b** being a mixture of diastereomers, in approximately **3b**:**3b′** = 80:20 ratio.

When the same reaction is undertaken with the 2-(aminoethyl)pyridine derivative, the same type of compound is formed, see Scheme 2. **3e** presents three stereogenic centers, and four pairs of enantiomers can be obtained. However, according to the shape and chemical shifts of the signals in the NMR spectra, only two diastereomers are observed with an approximately **3e**:**3e′** = 65:35 ratio.

The ^31^P{^1^H} spectra show in all cases the phosphorus signals corresponding to two phosphorus atoms in *cis* disposition, though in the case of **3e**, two pairs of signals are observed due to the different diastereomers. In the case of **3b**, only the signal of the major diastereomer can be detected. In the ^13^C{^1^H} NMR spectra, two sets of signals are observed at ca. 210 ppm and ca. 230 ppm. Those at a higher field show a small coupling constant and are assigned to the acyl groups *cis* to a phosphorus atom. The second group is assigned to the iminiumacyl groups and shows a large coupling constant due to coupling to *trans* phosphorus atoms.

**Figure 4:**
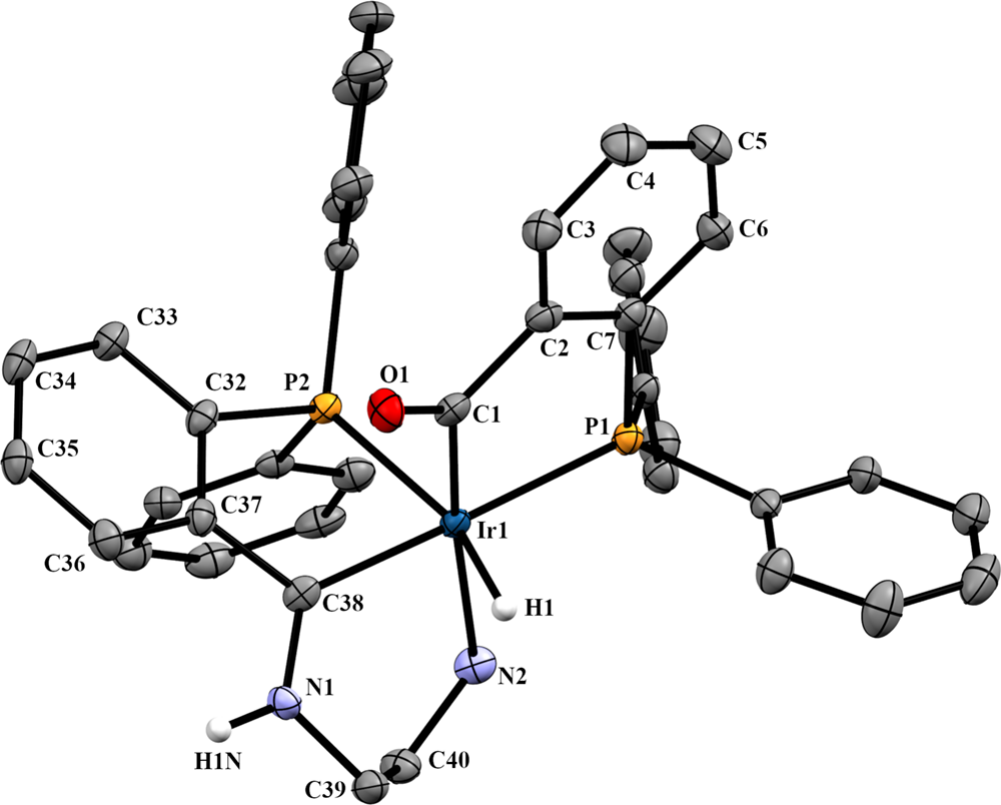
Molecular structure of **3a**, *OC-*6-56-*A* isomer. Displacement ellipsoids are drawn at the 50% probability level. Most of hydrogen atoms, counteranions, and crystallized methanol and pentane molecules have been omitted for clarity. Selected bond lengths (Å) and angles (°): Ir1–P1, 2.2984(6); Ir1–P2, 2.3425(7); Ir1–C1, 2.0306(5); Ir1–C38, 2.0450(5); Ir1–N2, 2.2163(6); Ir1–H1, 1.4193(5); C38–N1, 1.2911(3); P1–Ir1–C1, 84.5(1); P2–Ir1–C38, 78.4(1); P2–Ir1–C1, 89.8(1); P1–Ir1–C38, 177.1(1); C38–Ir1–N2, 88.1(2); C2–C1–O1, 116.6(4); C37–C38–N1, 117.3(4).

**Figure 5:**
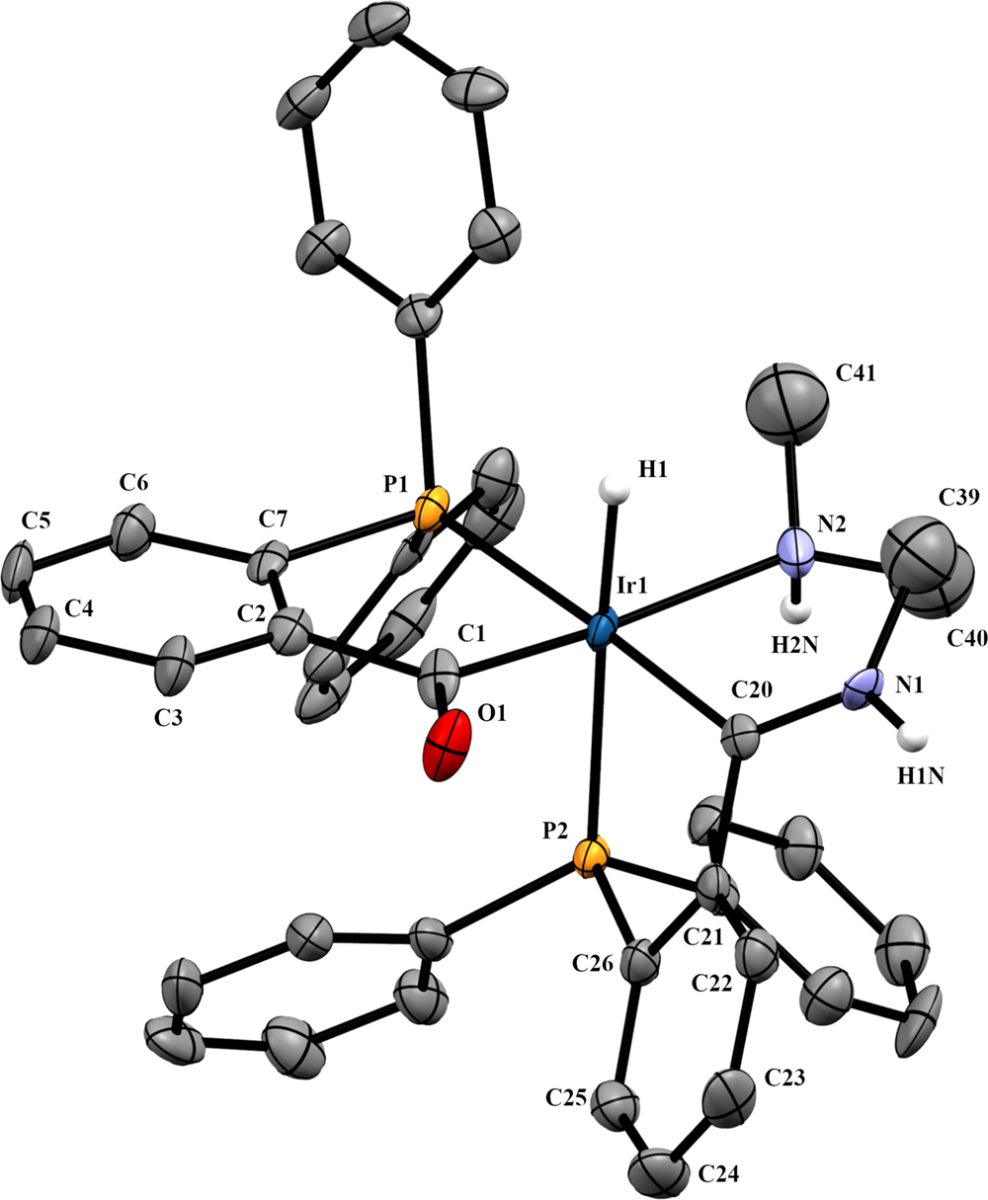
Molecular structure of **3b**, *OC-*6-56-*C,S* isomer. Displacement ellipsoids are drawn at the 50% probability level. Most of hydrogen atoms, counteranions, and crystallized chloroform molecules have been omitted for clarity. Selected bond lengths (Å) and angles (°): Ir1–P1, 2.3241(7); Ir1–P2, 2.3425(5); Ir1–C1, 2.0258(6); Ir1–C20, 2.0390(6); Ir1–N2, 2.2846(7); Ir1–H1, 1.4955(3); C20–N1, 1.2966(3); P1–Ir1–C1, 84.5(3); P2–Ir1–C20, 78.2(2); P2–Ir1–C1, 89.4(3); P1–Ir1–C20, 175.7(2); C20–Ir1–N2, 86.5(3); C2–C1–O1 116.6(8); C21–C20–N1, 116.1(7).

The X-ray analysis of **3a** and **3b** confirms the structure suggested by the spectroscopic techniques, and in both cases, the phosphorus atom *trans* to hydride belongs to the tridentate PCN^amine^ ligand (Figures 4 and 5). In both cases, the space group of the unit cell is P1̅, which indicates that both crystallize as a mixture of enantiomers; in the case of **3b**, only one of the diastereomers (the A,R–C,S pair of enantiomers) has been crystallized.

On the other hand, upon dissolution of the ketoimine-type compounds, **2a** and **2b**, in a THF/H_2_O = 1:1 mixture, the expected slow amine coordination occurs, but in this case, the isomerization appears limited, and a mixture of two isomers **4a/3a** = 80:20 or **4b/3b** = 60:40, as shown in Scheme 1ii, is formed after 24 h. In both cases, the major product consists of a coordinated amino group that is *trans* to hydride. The formation of **4** involves displacement of chloride by amine with retention of the conformation of the starting material. This behavior appears unusual upon cleavage of the hydrogen bond in irida-β-ketoimines and may be probably related to the increased polarity of the used solvent, inhibiting the isomerization. Isomers **3** with hydride trans to phosphine, a more frequently observed disposition, fail to transform into isomers **4** in THF/H_2_O. Longer reaction times allow some further slow transformation of **4** into **3**, which fails to reach completion. The **4**/**3** mixtures were isolated and characterized as perchlorate compounds. These **4**/**3** mixtures of complexes are also obtained when reacting complex **1** with ligands 1,2-diaminoethane (**a**) or *N*-methyl-1,2-diaminoethane (**b**) in THF/H_2_O = 1:1. As expected, complex **4b** is formed by two diastereomers, which are present in a **4b**:**4b′** = 95:5 ratio. The hydride resonances appear as triplet at −19.63 and −19.84 ppm for the major and minor isomers, respectively. The ^31^P{^1^H} spectra show doublets at 6.6 and 33.8 ppm for the major isomer and at 11.5 and 28.9 ppm for the minor isomer.

Even though chloride- and nitrogen-containing ligands can have a similar *trans* influence, the synthesis of **4a** and **4b** can be easily ascertained from the hydride region of the ^1^H NMR, where displacement of the signal toward a lower field, larger for the 1,2-diaminoethane derivative, is observed (see Experimental Section for details). That no further changes occur in the structure, compared to **2a** and **2b**, can be surmised from the largely unchanged nature of ^31^P{^1^H} NMR and ^13^C{^1^H} NMR.

**Figure 6:**
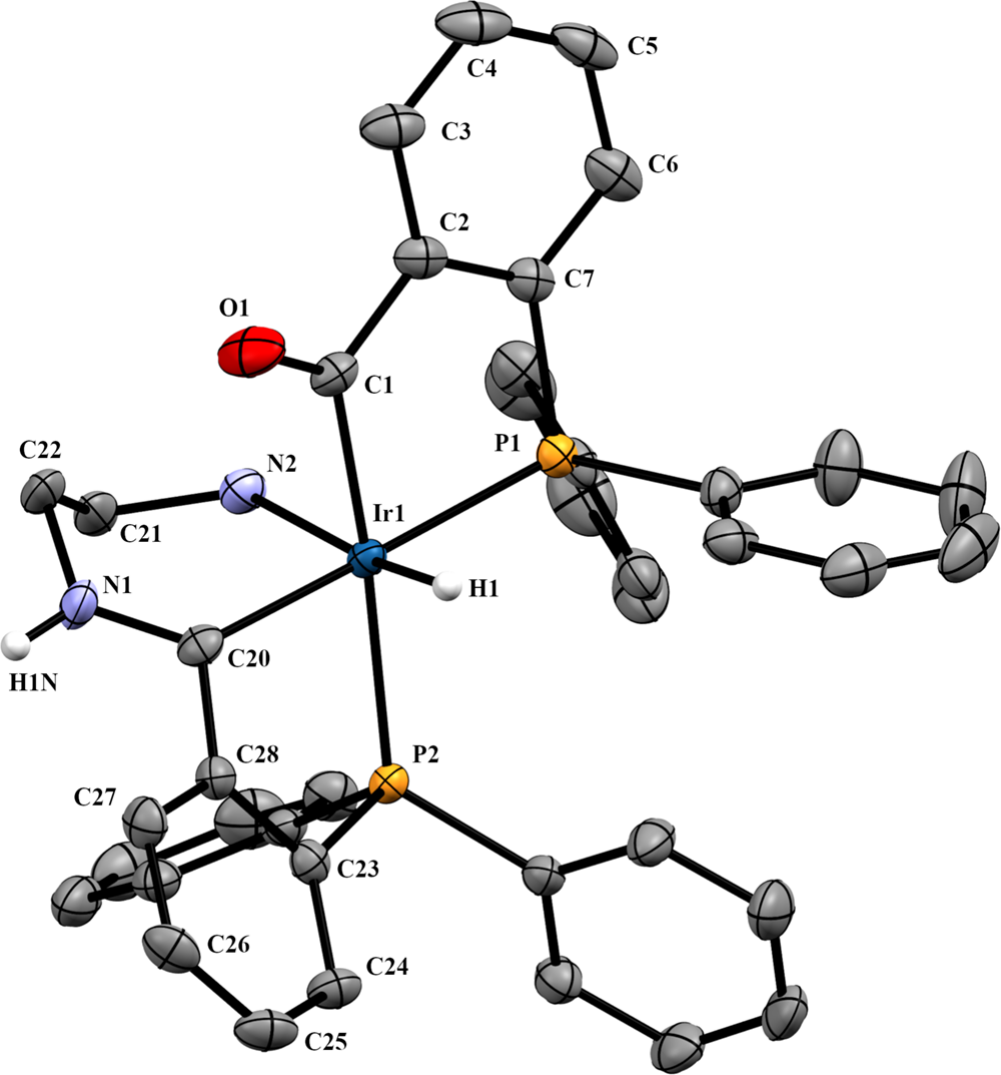
Molecular structure of **4a**, *OC*-6-54-*A* isomer. Displacement ellipsoids are drawn at the 50% probability level. Most of hydrogen atoms, counteranions, and crystallized diethyl ether molecules have been omitted. Selected bond lengths (Å) and angles (°): Ir1–P1, 2.3029(8); Ir1–P2, 2.3508(8); Ir1–C1, 2.079(3); Ir1–C20, 2.038(3); Ir1–N2, 2.232(3); Ir1–H1, 1.57(3); C20–N1, 1.296(4); P1–Ir1–C1, 83.22(9); P2–Ir1–C20, 76.81(9); P2–Ir1–C1, 169.10(9); P1–Ir1–C20, 177.2(1); C20–Ir1–N2, 88.5(1); C2–C1–O1, 117.2(3); C28–C20–N1, 115.9(3).

The structure proposed was confirmed by X-ray diffraction (see Figure 6). Compound **4a** crystallizes as a mixture of enantiomers in the P2_1_/c space group.

### Acyl-Imine-Type Complex Formation (**5**)

**Scheme 3:**
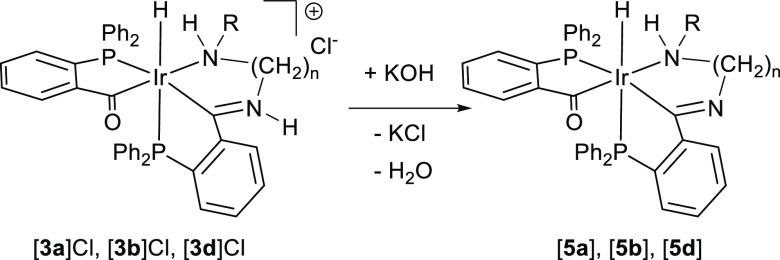
Formation of Neutral Acyl-Imine Compounds [**5**] from Cationic Acyl-Iminium Compounds [**3**]Cl

In the presence of strong bases such as potassium hydroxide, acyl-iminium complexes [**3a**]Cl-[**3d**]Cl undergo deprotonation to form neutral acyl-imine type of complexes [**5a**]–[**5d**], as shown in Scheme 3.

None of the spectroscopic signals is greatly altered, the Ir–H vibration appearing around 2020 cm^–1^ in the IR and the hydride signal at ca. −8.5 ppm in the ^1^H NMR, as a doublet of doublets, with one large coupling constant, due to coupling to a *trans* phosphorus atom, and a smaller coupling constant, assigned to a coupling to a phosphorus atom in *cis*. As is the case with compound **3b**, the neutral complex derived from *N*-methyl-1,2-diaminoethane, **5b**, also appears as a mixture of diastereomers, which maintains the same 80:20 ratio as in **3b**.

**Figure 7:**
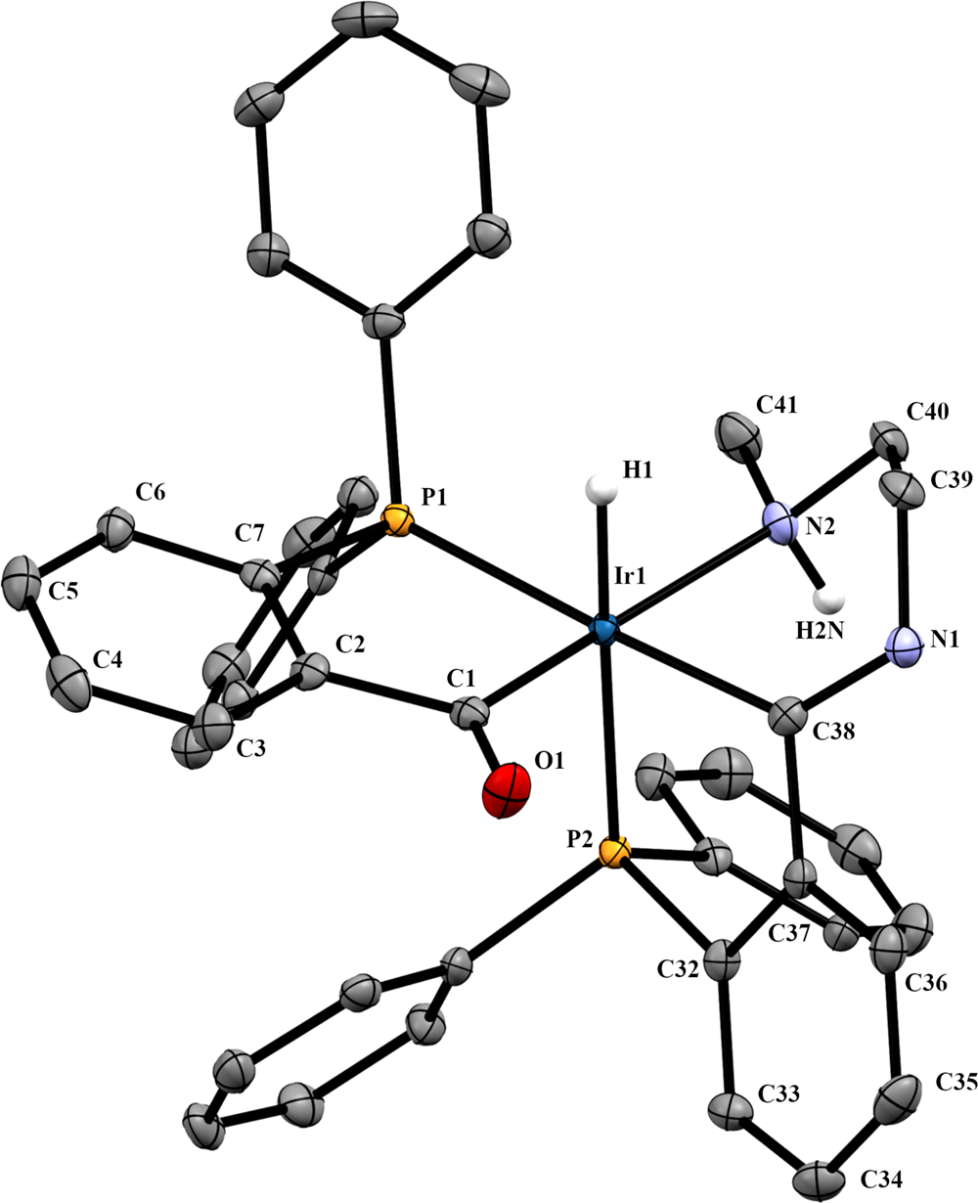
Molecular structure of **5b**, *OC*-6-56-*C,S* isomer. Displacement ellipsoids are drawn at the 50% probability level. Most of hydrogen atoms and crystallized chloroform and diethyl ether molecules have been omitted. Selected bond lengths (Å) and angles (°): Ir1–P1, 2.3145(10); Ir1–P2, 2.3268(10); Ir1–C1, 2.014(4); Ir1–C38, 2.077(4); Ir1–N2, 2.249(4); Ir1–H1, 1.59(6); C38–N1, 1.288(5); P1–Ir1–C1, 84.82(12); P2–Ir1–C38, 80.22(12); P2–Ir1–C1, 90.60(12); P1–Ir1–C38, 175.10(11); C20–Ir1–N2, 79.41(15); C2–C1–O1, 116.2(4); C37–C38–N1, 114.7(4).

The X-ray analysis of **5b** shows that it crystallizes in the P1̅ space group as a mixture of enantiomers, which, as in **3b**, are the C,S–A,R enantiomers. In addition, the structure, see Figure 7, reveals that the phosphorus atom *trans* to the hydride is one of the tridentate PCN^amine^ ligands; therefore, we can infer that the deprotonation of the acyl-iminium compounds is a simple deprotonation with no subsequent rearrangement reactions.

### Analysis of the X-ray Structures

The coordinative environment of the iridium atom in complexes **2a** (Figure 3) and **4a** (Figure 6) is a slightly distorted octahedron where four positions are occupied by the phosphorus and carbon atoms of the five-membered metallacycles. The other two positions are occupied by a hydride and a chlorine (**2a**) or the amine nitrogen (**4a**), which are mutually in the *trans* position. The Ir1–P1 distances, shorter than Ir1–P2, agree with a slightly larger *trans* influence for the acyl group than for the iminium group.^3^ The C_imine_–N bond lengths (1.2994(3) or 1.296(4) Å) evidence a substantial double bonding. In **2a**, the C2–C1–O1 and the C37–C38–N1 angles are 115.0(3) and 119.8(3)°, respectively. The O1···N1 distance, 2.653(3) Å, is in accordance with a moderate hydrogen bridge bond,^23^ and the O1–H2–N1 angle, 161.1°, is consistent with a nearly linear O···H–N bridge.

Schiff base formation leads to loss of coplanarity between the metallacycles and the aryl rings they are supported on. P–C chelates twist, and to a greater degree extent in the chelate bearing the imine carbon atom. The angle between the mean plane formed with P1, Ir1, and C1, the acylphosphine chelate, and the mean plane formed with the C2–C7 aryl ring form an angle of 15.03°. In **2a**, a larger angle of 25.07° between the mean plane formed with P2, Ir1, and C38 and the mean plane formed with the C32–37 aryl ring is observed. In compound **4a**, where the O···H–N hydrogen bond present in **2a** is broken with formation of an additional six-membered metallacycle, the angle between the mean plane formed with P2, Ir1, and C20 and the mean plane formed with the C23–C28 aryl ring is still larger, 38.69°. The bigger twist of the P2,C_imine_ chelate ring is also reflected in its bite angle, where P2–Ir–C_imine_ in **2a** is 81.5(1) and 76.8(1)°in **4a**.

Complexes **3a**, **3b**, and **5b** show an iridium(III) pseudo-octahedral environment with a hydride, a bidentate ligand [linked by the phosphorus atom (P1) and the carbon atom (C1) of the acyl group], and a PCN^amine^ terdentate ligand [linked by the phosphorus atom (P2), a sp^2^ carbon atom (C20), in iminium in **3a** and **3b** or imine in **5b**, and the amine group of the ligand (N2)]. The phosphorus of the bidentate ligand (P1) is in a trans position to the sp^2^ carbon atom (C_imine_). The distances and angles in **3a** and **3b** and also in **5b** are similar. The slight lengthening of the Ir–P1 bond, from 2.2984(6) Å in **3a** to 2.3241(7) Å in **3b**, is most likely due to steric reasons. In these complexes, significant changes in the twist of both chelate rings with respect to complex **4a** are observed. The chelate ring containing the acyl group recovers the planarity of **1** with angles between the mean plane formed with P1, Ir1, and C1 and the mean plane formed with the C2–C7 aryl ring of 8.16° for **3a**, 9.27° for **3b**, and 4.87° for **5b**. Meanwhile, the P2,C_imine_ chelate ring is also more planar than in **4a**, with a larger difference in the acyl-imino derivative **5b**, being the angle between the mean plane formed with P2, Ir1, and C_imine_ and the mean plane formed with the aryl ring that supports it 30.52 for **3a**, 29.18 for **3b**, and 23.79 for **5b**.

Interestingly, all bond lengths and angles are very similar in all the compounds presented in this work, regardless of the neutral or cationic nature of the metal center. Also, the geometry around the imine group does not change significantly in the different compounds. In all complexes containing the PCN^amine^ ligand, the six-membered metallacycles adopt a twisted boat conformation; curiously, the protonated compounds are more twisted than the neutral ones, thus the N2–C–C–N1 torsion angle for **5b** is 44.2(5)°, while that for **3a**, **3b**, and **4a** is 75.3(5), 82(1), and 75.1(3)°, respectively.

### Catalytic Methanolysis of Ammonia Borane

The efficiency of iridium-based homogeneous systems containing strong O···H···O intramolecular hydrogen bonds for the methanolysis under the air of ammonia-borane^17^ (see eq 1) led us to study the catalytic activity of our methanol-soluble compounds **2**, showing O···H···N hydrogen bond interactions, **3**, containing acyl-iminium functionalities and **5**, with acyl-imine moieties.
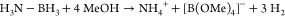
1

**Figure 8:**
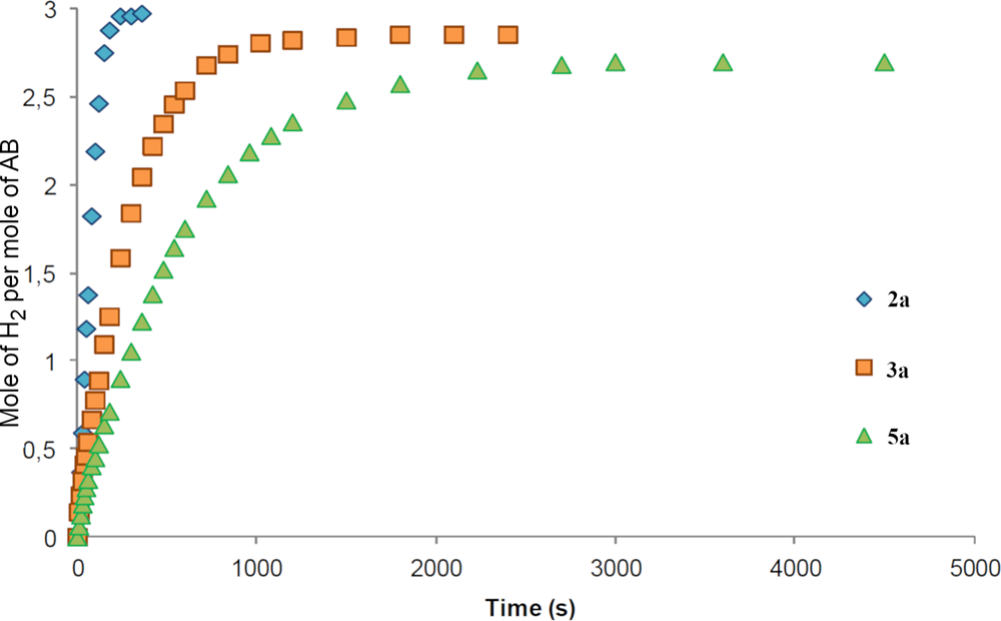
Hydrogen release in the methanolysis of AB by complexes **2a**, **3a**, and **5a** in methanol with 0.5% catalyst loading at 60 °C.

**Figure 9:**
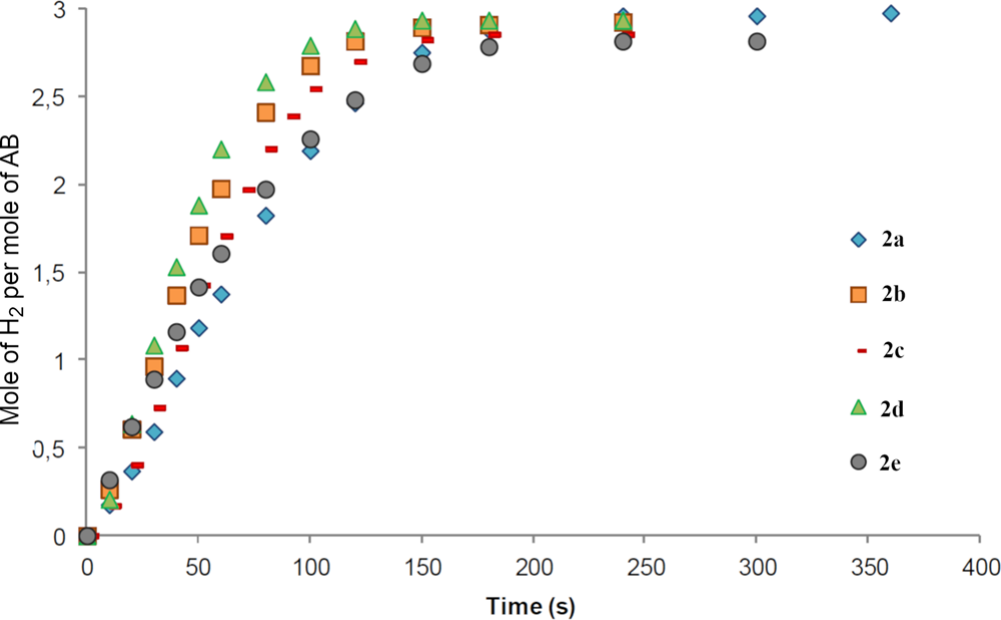
Hydrogen release in the methanolysis of AB by complexes **2a**, **2b**, **2c**, **2d**, and **2e** in methanol with 0.5% catalyst loading at 60 °C.

To first ascertain the most efficient type of compound, a comparison between **2a**, **3a**, and **5a** was carried out (see Figure 8). These compounds catalyze the release of H_2_, and worth noting is the ability of complexes **3a** and **5a**, containing PCN^amine^ ligands, most likely related to their hemilabile character. When using the initial AB concentration of 0.46 M and a 0.5 mol % catalyst loading at 60 °C, the fastest reaction occurs with the ketoimine complex **2a**, releasing 3 equiv of hydrogen in 240 s (TOF_50%_ of 257 mol_H2_ mol^–1^ min^–1^), while **3a** releases 2.9 equiv of hydrogen in 1200 s (TOF_50%_ of 78 mol_H2_ mol^–1^ min^–1^) and **5a** needs 3000 s to release 2.8 equiv of hydrogen (TOF_50%_ of 37 mol_H2_ mol^–1^ min^–1^). These results show that ketoimine **2a** or iminium **3a** complexes, containing an acidic functionality, are more active. The activity of the ketoimine derivative, with coordinated chloride instead of amine and a O···H–N fragment, appears significantly higher, though lower than that of complex **1** containing a O···H···O fragment. On view of these results, we compared the activity of ketoimine complexes **2**, shown in Figure 9, with **2d**, derived from 1,3-diaminopropane, being the most efficient catalyst among them, needing only 120 s to release 2.9 equiv of hydrogen (TOF_50%_ of 473 mol_H2_ mol^–1^ min^–1^), followed by **2b**, which needs 150 s (TOF_50%_ of 400 mol_H2_ mol^–1^ min^–1^) to release the same amount of hydrogen. Meanwhile, **2c** needs 150 s to release 2.8 equiv, and **2e** releases the same amount of hydrogen in 180 s, both with a TOF_50%_ of 327 mol_H2_ mol^–1^ min^–1^.

In situ multinuclear NMR experiments in CD_3_OD were carried out with **2a** because its NMR signals are easier to be identified than those of **2d**. The ^11^B NMR spectrum of a freshly prepared solution in CD_3_OD shows the presence of the H_3_N-BH_3_ substrate, the anionic reaction product [B(OCH_3_)_4_]^−^, and also of minor amounts of two ammonia-methoxyborane adduct intermediates, H_3_N-BH_2_(OCH_3_) and H_3_N-BH(OCH_3_)_2_ (see SI Figure S86) indicating successive and parallel methanolysis steps for the whole substrate as in the reaction catalyzed by the irida-β-diketone **1**.^17^ In the corresponding mechanism, a vacancy around the metal was proposed as required to allow coordination of AB or of the intermediate methanolysis products for complete borane dehydrogenation to occur. When using **2d** (TOF_50%_ of 473 mol_H2_ mol^–1^ min^–1^), the methanolysis of AB is slower than when using **1** (TOF_50%_ of 865 mol_H2_ mol^–1^ min^–1^), and we believe this can be due to a combination of a reduced Bronsted acidity of the OHN proton in the keitimine complex and the presence of the dangling amine functionality that may occupy the required vacancy, thus inhibiting borane coordination. The latter is in accordance with **2d** being the most efficient among complexes **2**, because upon amine coordination, a seven-membered metallacycle is formed, thus leading to a less competitive reaction than with other complexes **2** able to afford six-membered metallacycles. ^1^H NMR spectra show the release of HD, but unfortunately a myriad of compounds are formed, and no conclusion about the identity of the active species can be gathered, thus precluding any reliable specific mechanistic proposal (see SI Figure S87).

As when using complex **1**, the kinetic profile obtained in the methanolysis of AB catalyzed by complex **2d** at 60 °C can be considered to follow a pseudo-first-order reaction rate model with respect to the substrate, as shown by the linear plots^6a^ in SI, Figure S89, which was applied to determine the overall rate constants, *k*_obs_. The rate of the hydrogen release also depends on the catalyst loading (SI, Figure S88). Assuming a first-order dependence with respect to the substrate, the rate law agrees with *v*_exp_ = *k*_cat_[catalyst]_0_[substrate], where *k*_cat_[catalyst]_0_ = *k*_obs_. A plot of the pseudo-first-order rate constant (*k*_obs_) versus [catalyst]_0_ in the 1.86 × 10^–3^ to 0.46 × 10^–3^ M range (SI, Table S2 and Figure S90) allows the proposal of a first-order dependence on the catalyst and *k* = 6.9 ± 0.6 M^–1^ s^–1^. However, Figure S90 shows a substantial intercept, which could be related to some complexity during the first stages of the reaction.

## Conclusions

The reaction of hydridoirida-β-diketones with aliphatic diamines leads to hydridoirida-β-ketoimines with dangling amine, whose coordination in polar solvents afford cationic hydridoacyl compounds with new terdentate hemilabile PCN^amine^ ligands containing iminium functionalities, which in basic media afford imine functionalities. The PCN^amine^ moieties adopt a facial disposition, and high diastereoselectivity is obtained in complexes derived from mixed primary/secondary diamines. All these complexes behave as homogeneous catalysts for the methanolysis of ammonia-borane in air to release hydrogen with hydridoirida-β-ketoimines with dangling amine being the most efficient.

## Experimental Section

### General

Synthetic procedures were carried out at room temperature under nitrogen by standard Schlenk techniques. [IrHCl{(PPh_2_(*o*-C_6_H_4_CO))_2_H}] (**1**)^24^ was prepared as previously reported. All other reagents were purchased from commercial sources and used without further purification. Microanalysis was carried out using a Leco Truspec Micro microanalyzer. IR spectra were recorded using a Nicolet FTIR 510 spectrophotometer in the range 4000–400 cm^–1^ using KBr pellets. ^1^H NMR and ^13^C{^1^H} (TMS internal standard), ^31^P{^1^H} and ^31^P NMR (H_3_PO_4_ external standard), and ^11^B (BF_3_·Et_2_O external standard) NMR spectra were recorded using a Bruker Avance DPX 300 or Bruker Avance 400 or Bruker Avance 500 spectrometer.

*Warning*: Perchlorate salts and transition-metal perchlorate complexes may be explosive. Preparations on a scale larger than that reported herein should be avoided.

### Methanolysis

A solution of 1.16 mmol of the desired amine-borane adduct in 2 mL of methanol was prepared in a round-bottom 40 mL flask fitted with a gas outlet and a side arm sealed with a tight-fitting septum cap. The flask was connected via the gas outlet to a gas burette filled with water. The amine-borane adduct solution was immersed in a thermostated water bath to reach the desired temperature under atmospheric pressure (1 atm) and in the presence of air. A solution of the selected precatalyst, in 0.5 mL of methanol, was syringed through the septum into the reaction flask, connected with a magnetic stirrer, and timing started. Gas evolution began immediately, and the released gas was measured by determining periodically the volume of water displaced in the burette.

### X-ray Crystallographic Structure Determination

Crystals for **2a**, [**3a**]Cl, [**3b**]Cl, [**4a**]ClO_4_, and **5b** were mounted on a glass fiber and used for data collection on a Bruker D8 Venture with a Photon detector equipped with graphite monochromated Mo Kα radiation (λ = 0.71073 Å). The data reduction was performed with the APEX2^25^ software and corrected for absorption using SADABS.^26^ Crystal structures were solved by direct methods using the SIR97 program^27^ and refined by full-matrix least-squares on *F*^2^ including all reflections using anisotropic displacement parameters by means of the WINGX crystallographic package.^28^ Generally, anisotropic temperature factors were assigned to all atoms except for hydrogen atoms, which are riding their parent atoms with an isotropic temperature factor arbitrarily chosen as 1.2 times that of the respective parent. Hydrides were clearly located as a Fourier peak in a difference map and then fixed. Final *R*(*F*), *wR*(*F*^2^), and goodness-of-fit agreement factors and details on the data collection and analysis can be found in SI, Table S1.

### Synthesis of Ketoimine Compounds **2**

The corresponding diamine (0.048 mmol) was added to a Schlenk flask charged with a THF suspension of **1** (0.037 mmol). Then, the suspension turned yellow, and the solution was stirred for 2 h. Then, the solvent was removed under vacuum to afford a yellow solid that was washed with diethyl ether and then hexane and dried under vacuum.

#### *OC*-6-65—[IrHCl{(PPh_2_(*o*-C_6_H_4_CO))(PPh_2_(*o*-C_6_H_4_CN(CH_2_)_2_NH_2_))H}] (**2a**)

Yield 86%. IR (KBr, cm^–1^): 3372 (w, N–H); 2177 (s, Ir–H); 1552 (s, C=O and C=N). Elemental Analysis for IrC_40_H_36_P_2_ON_2_Cl: Calculated: C 56.50, H 4.27, N 3.29. Found: C 56.21, H 3.96, N 3.02. ^1^H NMR (300 MHz, CDCl_3_): δ −20.47 (t, ^2^*J*_P,H_ = 14.4 Hz, 1H, *H*-Ir); 3.10 (dt, ^2^*J*_H,H_ = 4.9 Hz, ^2^*J*_H,H_ = 13.4, 1H, *H*_2_C-NH_2_); 3.28 (m, 1H, *H*_2_C-NH_2_); 4.15 (p, ^2^*J*_H,H_ = 5.7 Hz, ^2^*J*_H,H_ = 6.3, 2H, *H*_2_C-NC); 7–8.1 (28H, aromatics); 12.82 (br, 1H, O--*H*--N) ppm. ^31^P{^1^H} NMR (161.96 MHz, CDCl_3_): δ 15.6 (d, ^2^*J*_P,P_ = 7 Hz); 29.9 (d, ^2^*J*_P,P_ = 7 Hz) ppm. ^13^C{^1^H} NMR (125.78 MHz, CDCl_3_): δ 42.1 (s, *C*H_2_-NH_2_); 54.7 (d, ^2^*J*_P,C_ = 5 Hz, H_2_*C*-NC); 123.0–162.0 (aromatics); 224.0 (d, ^2^*J*_P,C_ = 102 Hz, *C*=O or *C*=N); 243.0 (d, ^2^*J*_P,C_ = 106 Hz, *C*=O or *C*=N) ppm.

Yellow single crystals of **2a** suitable for X-ray diffraction were obtained by vapor diffusion of diethyl ether into a solution of **2a** in methanol at −20 °C.

#### *OC*-6-65—[IrHCl{(PPh_2_(*o*-C_6_H_4_CO))(PPh_2_(*o*-C_6_H_4_CN(CH_2_)_2_NHCH_3_))H}] (**2b**)

Yield 66%. IR (KBr, cm^–1^): 3280 (w, N–H); 2171 (s, Ir–H); 1564 (s, C=O and C=N). Elemental Analysis for IrC_41_H_38_P_2_ON_2_Cl·H_2_O:

Calculated: C 55.81, H 4.57, N 3.17. Found: C 55.82, H 4.68, N 3.34. ^1^H NMR (400 MHz, CDCl_3_): δ −20.44 (t, ^2^*J*_P,H_ = 14.5 Hz, 1H, *H*-Ir); 2.18 (s, 3H, *H*_3_C-NH); 2.94 (m, 1H, *H*_2_C-NH); 3.19 (m, 1H, *H*_2_C-NH); 4.16 (m, 1H, *H*_2_C-NC); 4.34 (m, 1H, *H*_2_C-NC); 6.9–8.1 (28H, aromatics); 12.62 (br, 1H, O--*H*--N) ppm. ^31^P{^1^H} NMR (161.96 MHz, CDCl_3_): δ 16.0 (d, ^2^*J*_P,P_ = 7.4 Hz); 29.9 (d, ^2^*J*_P,P_ = 7.4 Hz) ppm. ^13^C{^1^H} NMR (125.76 MHz, CDCl_3_): δ 35.6 (s, *C*H_3_-NH); δ 50.7 (s, *C*H_2_-NH); 50.9 (d, ^2^*J*_P,C_ = 5 Hz, H_2_*C*-NC); 122.0–162.0 (aromatics); 224.3 (d, ^2^*J*_P,C_ = 102 Hz, *C*=O or *C*=N); 242.0 (d, ^2^*J*_P,C_ = 104 Hz, *C*=O or *C*=N) ppm.

#### *OC*-6-65—[IrHCl{(PPh_2_(*o*-C_6_H_4_CO))(PPh_2_(*o*-C_6_H_4_CN(CH_2_)_2_NHCH_2_CH_3_))H}] (**2c**)

Yield 72%. IR (KBr, cm^–1^): 3268 (w, N–H); 2179 (s, Ir–H); 1553 (s, C=O and C=N). Elemental Analysis for IrC_42_H_40_P_2_ON_2_Cl: Calculated: C 57.43, H 4.59, N 3.19. Found: C 57.67, H 4.53, N 3.45. ^1^H NMR (400 MHz, CDCl_3_): δ −20.40 (t, ^2^*J*_P,H_ = 14.5 Hz, 1H, *H*-Ir); 0.83 (m, 3H, *H*_3_C-H_2_C-NH); 2.46 (m, 2H, H_3_C-*H*_2_C-NH); 3.00 (m, 1H, *H*_2_C-H_2_C-NC); 3.22 (m, 1H, *H*_2_C-H_2_C-NC); 4.15 (m, 1H, *H*_2_C-NC); 4.30 (m, 1H, *H*_2_C-NC); 6.9–8.1 (28H, aromatics); 12.64 (br, 1H, O--*H*--N) ppm. ^31^P{^1^H} NMR (161.96 MHz, CDCl_3_): δ 16.0 (d, ^2^*J*_P,P_ = 7.4 Hz); 30.4 (d, ^2^*J*_P,P_ = 7.4 Hz) ppm. ^13^C{^1^H} NMR (100.61 MHz, CDCl_3_): δ 15.0 (s, *C*H_3_-CH_2_-NH); 43.6 (s, CH_3_-*C*H_2_-NH); 48.9 (s, *C*H_2_-CH_2_-NC); 50.9 (d, ^2^*J*_P,C_ = 5.6 Hz, H_2_*C*-NC); 122.0–162.0 (aromatics); 224.2 (d, ^2^*J*_P,C_ = 102 Hz, *C*=O or *C*=N); 242.0 (d, ^2^*J*_P,C_ = 104 Hz, *C*=O or *C*=N) ppm.

#### *OC*-6-65—[IrHCl{(PPh_2_(*o*-C_6_H_4_CO))(PPh_2_(*o*-C_6_H_4_CN(CH_2_)_3_NH_2_))H}] (**2d**)

Yield 70%. IR (KBr, cm^–1^): 3280 (w, N–H); 2189 (s, Ir–H); 1553 (s, C=O and C=N). Elemental Analysis for IrC_41_H_38_P_2_ON_2_Cl: Calculated: C 56.97, H 4.43, N 3.24. Found: C 56.89, H 4.50, N 3.16. ^1^H NMR (400 MHz, CDCl_3_): δ −20.66 (t, ^2^*J*_P,H_ = 14.0 Hz, 1H, *H*-Ir); 2.08 (m, 2H, H_2_C-*H*_2_C-CH_2_); 2.88 (m, 2H, *H*_2_C-NH_2_); 4.11 (m, 2H, *H*_2_C-NC); 6.9–8.1 (28H, aromatics); 12.99 (br, 1H, O--*H*--N) ppm. ^31^P{^1^H} NMR (161.96 MHz, CDCl_3_): δ 14.6 (d, ^2^*J*_P,P_ = 7 Hz); 29.6 (d, ^2^*J*_P,P_ = 7 Hz) ppm. ^13^C{^1^H} NMR (100.60 MHz, CDCl_3_): δ 33.3 (s, CH_2_-*C*H_2_-CH_2_); δ 39.4 (s, *C*H_2_-NH_2_); 48.9 (d, ^2^*J*_P,C_ = 5.5 Hz, H_2_*C*-NC); 122.0–162.0 (aromatics); 221.4 (d, ^2^*J*_P,C_ = 103 Hz, *C*=O or *C*=N); 243.4 (d, ^2^*J*_P,C_ = 106 Hz, *C*=O or *C*=N) ppm.

#### *OC*-6-65—[IrHCl{(PPh_2_(*o*-C_6_H_4_CO))(PPh_2_(*o*-C_6_H_4_CNCH_2_(C_5_H_9_NH)))H}] (**2e**)

Yield 76%. IR (KBr, cm^–1^): 3276 (w, N–H); 2170 (s, Ir–H); 1554 (s, C=O and C=N). Elemental Analysis for IrC_44_H_42_P_2_ON_2_Cl·(H_2_O)_0.75_: Calculated: C 57.57, H 4.78, N 3.05. Found: C 57.75, H 4.77, N 2.61. ^1^H NMR (300 MHz, CDCl_3_): δ −20.57 (dd, ^2^*J*_P,H_ = 14.8 Hz, ^2^*J*_P,H_ = 13.6 Hz, 1H, *H*-Ir); −20.44 (dd, ^2^*J*_P,H_ = 15.6 Hz, ^2^*J*_P,H_ = 14.1 Hz, 1H, *H*-Ir); 1.17 (m, 1H, *H*_2_C-NH); 1.33 (m, 1H, *H*_2_C-CH_2_-CH) and (m, 1H, *H*_2_C-CH_2_-NH); 1.39 (m, 1H, *H*_2_C-CH_2_-CH) and (m, 1H, *H*_2_C-CH_2_-NH); 1.46 (m, 1H, *H*_2_C-NH); 1.51 (m, 2H, *H*_2_C-CH_2_-CH); 1.72 (m, 1H, *H*_2_C-NH); 1.77 (m, 1H, *H*_2_C-CH_2_-NH); 1.84 (m, 1H, *H*_2_C-CH_2_-NH); 1.86 (m, 1H, *H*_2_C-NH); 2.55 (m, 1H, *H*_2_C-CH); 2.57 (m, 1H, *H*_2_C-CH); 2.76 (m, 1H, *H*_2_C-CH); 2.88 (m, 1H, *H*_2_C-CH); 3.07 (m, 1H, *H*C); 3.24 (m, 1H, *H*C); 4.00 (m, 1H, *H*_2_C-NC); 4.01 (m, 1H, *H*_2_C-NC); 4.10 (m, 1H, *H*_2_C-NC); 4.16 (m, 1H, *H*_2_C-NC); 6.8–8.0 (56 H, aromatics); 12.77 (br, 1H, O--*H*--N); 12.98 (br, 1H, O--*H*--N) ppm. ^31^P{^1^H} NMR (161.96 MHz, CDCl_3_): δ 15.07 (s, br); 16.91 (s, br); 29.13 (d, ^2^*J*_P,P_ = 7.3 Hz); 29.89 (d, ^2^*J*_P,P_ = 7.0 Hz) ppm. ^13^C{^1^H} NMR (100.60 MHz, CDCl_3_): 24.4 (s, *C*H_2_-NH); 24.5 (s, *C*H_2_-NH); 25.6 (s, *C*H_2_-CH_2_-NH); 25.7 (s, *C*H_2_-CH_2_-NH); 29.6 (s, *C*H_2_-CH_2_-CH); 30.1 (s, *C*H_2_-CH_2_-CH); 46.2 (s, *C*H_2_-CH); 46.6 (s, *C*H_2_-CH); 56.2 (s, *C*H); 56.6 (s, *C*H); 56.8 (s, *C*H_2_-NC); 57.6 (s, *C*H_2_-NC); 122.0–162.0 (aromatics); 224.1 (d, ^2^*J*_P,C_ = 102 Hz, *C*=O or *C*=N); 242.0 (d, ^2^*J*_P,C_ = 104 Hz, *C*=O or *C*=N); 242.8 (d, ^2^*J*_P,C_ = 104 Hz, *C*=O or *C*=N) ppm.

### Synthesis of Acyl-Iminium Type of Compounds [**3**]Cl

The corresponding ketoimine precursor **2** (0.037 mmol) was stirred in a 1:1 solution of methanol:dichloromethane for 24 h after which the solvent was removed under vacuum. The resulting yellow solid was washed with diethyl ether and dried in vacuum.

#### *OC*-6-56—[IrH(PPh_2_(*o*-C_6_H_4_CO))(PPh_2_(*o*-C_6_H_4_C=N(*H*)CH_2_CH_2_NH_2_))]Cl ([**3a**]Cl)

Yield 77%. IR (KBr, cm^–1^): 3316 (w, N–H); 2015 (s, Ir–H); 1575 (s, C=O and C=N). Elemental Analysis for IrC_40_H_36_P_2_ON_2_Cl·(CH_2_Cl_2_)_0.25_: Calculated: C 55.47, H 4.22, N 3.21. Found: C 55.16, H 4.37, N 3.02. ^1^H NMR (300 MHz, CD_3_OD): δ −8.74 (dd, ^2^*J*_P,H_ = 122.0 Hz, ^2^*J*_P,H_ = 18.4 Hz, 1H, *H*-Ir); 1.46 (m, 1H, *H*_2_C-NH_2_); 1.89 (m, 1H, N*H*_2_); 2.80 (m, 1H, *H*_2_C-NH_2_); 3.88 (m, 1H, *H*_2_C-NC); 3.96 (m, 1H, *H*_2_C-NC); 4.71 (m, 1H, N*H*_2_); 7–8.1 (28H, aromatics) ppm. ^31^P NMR (161.96 MHz, CD_3_OD): δ 15.5 (d, ^2^*J*_H,P_ = 122 Hz); 25.8 (s, br) ppm. ^13^C{^1^H} NMR (125.78 MHz, CDCl_3_): δ 39.7 (s, *C*H_2_-NH_2_); 55.8 (s, H_2_*C*-NC); 123.0–162.0 (aromatics); 217.8 (dd, ^2^*J*_P,C_ = 16 Hz, ^2^*J*_P,C_ = 6.2 Hz, *C*=O); 232.2 (d, ^2^*J*_P,C_ = 90 Hz, *C*=N) ppm.

Yellow single crystals of [**3a**]Cl were obtained by vapor diffusion of diethyl ether into a solution of [**3a**]Cl in methanol at −20 °C.

#### *OC*-6-56—[IrH(PPh_2_(*o*-C_6_H_4_CO))(PPh_2_(*o*-C_6_H_4_C=N(*H*)CH_2_CH_2_NHCH_3_))]Cl ([**3b**]Cl)

Yield 70%. IR (KBr, cm^–1^): 3282 and 3198 (w, N–H); 2014 (s, Ir–H); 1575 (s, C=O and C=N). Elemental Analysis for IrC_41_H_38_P_2_ON_2_Cl·(CH_2_Cl_2_): Calculated: C 53.14, H 4.25, N 2.95. Found: C 52.78, H 4.02, N 2.46. ^1^H NMR (400 MHz, CDCl_3_): δ −9.15 (dd, ^2^*J*_P,H_ = 124.5 Hz, ^2^*J*_P,H_ = 18.9 Hz, 1H, *H*-Ir); −8.46 (dd, ^2^*J*_P,H_ = 122.6 Hz, ^2^*J*_P,H_ = 19.8 Hz, 1H, *H*-Ir); 2.36 (d, ^3^*J*_H,H_ = 6 Hz, 3H, C*H*_3_); 2.36 (m, 1H, C*H*_2_-NC); 2.60 (m, 1H, C*H*_2_-NC); 4.00 (m, 1H, C*H*_2_-NIr); 4.47 (m, 1H, C*H*_2_-NH); 6.3–8.7 (56H, aromatics) ppm. ^31^P NMR (161.96 MHz, CDCl_3_): δ 15.4 (d, ^2^*J*_H,P_ = 127 Hz); 25.7 (s, br) ppm. ^13^C{^1^H} NMR (100.61 MHz, CDCl_3_): δ 45.6 (s, *C*H_3_); 52.5 (s, *C*H_2_-NC); 54.5 (s, *C*H_2_-NIr); 120–160 (aromatics); 208.4 (s, *C*=O); 228.8 (d, ^2^*J*_P,C_ = 92 Hz, *C*=N).

Yellow single crystals of [**3b**]Cl were obtained by vapor diffusion of hexane into a solution of [**3b**]Cl in chloroform at −20 °C.

#### *OC*-6-56—[IrH(PPh_2_(*o*-C_6_H_4_CO))(PPh_2_(*o*-C_6_H_4_C=N(*H*)CH_2_CH_2_CH_2_NH_2_))]Cl ([**3d**]Cl)

Yield 70%. IR (KBr, cm^–1^): 3311 (w, N–H); 2036 (s, Ir–H); 1575 (s, C=O and C=N). Elemental Analysis for IrC_41_H_38_P_2_ON_2_Cl·(H_2_O)_0.5_: Calculated: C 56.38, H 4.50, N 3.21. Found: C 56.21, H 4.66, N 2.40. ^1^H NMR (400 MHz, CDCl_3_): δ −8.36 (dd, ^2^*J*_P,H_ = 126.3 Hz, ^2^*J*_P,H_ = 19.1 Hz, 1H, *H*-Ir); 1.55 (m, 1H, N*H*_2_); 1.94 (m, 2H, CH_2_-*H*_2_C-CH_2_); 2.56 (m, 2H, *H*_2_C-NH_2_); 2.93 (m, 1H, N*H*_2_); 4.56 (m, 1H, *H*_2_C-NC); 4.85 (m, 1H, *H*_2_C-NC); 6.2–8.9 (28H, aromatics) 12.79 (br, 1H, *H*N=C) ppm. ^31^P{^1^H} NMR (161.96 MHz, CDCl_3_): δ 18.2 (d, br, ^2^*J*_P,P_ = 12 Hz); 27.0 (d, ^2^*J*_P,P_ = 12 Hz) ppm. ^13^C{^1^H} NMR (100.61 MHz, CDCl_3_): δ 28.9 (s, CH_2_-*C*H_2_-CH_2_); 44.1 (s, H_2_*C*-NH_2_); 51.2 (s, H_2_*C*-NC); 122.0–162.0 (aromatics); 211.2 (d, ^2^*J*_P,C_ = 5.7 Hz, *C*=O); 226.7 (d, ^2^*J*_P,C_ = 93 Hz, *C*=N) ppm.

#### *OC*-6-56—[IrH(PPh_2_(*o*-C_6_H_4_CO))(PPh_2_(*o*-C_6_H_4_C=N(*H*)CH_2_(C_5_H_9_NH)))]Cl ([**3e**]Cl)

Yield 73.5%. IR (KBr, cm^–1^): 3320 and 3238 (w, N–H); 2086 (s, Ir–H); 1560 (s, C=O and C=N). Elemental Analysis for IrC_44_H_42_P_2_ON_2_Cl·(CH_2_Cl_2_)_0.75_: Calculated: C 55.52, H 4.53, N 2.89. Found: C 55.63, H 4.35, N 2.73. ^1^H NMR (400 MHz, CDCl_3_): δ −9.08 (dd, ^2^*J*_P,H_ = 124.2 Hz, ^2^*J*_P,H_ = 19.8 Hz, 1H, *H*-Ir); −8.56 (dd, ^2^*J*_P,H_ = 122.9 Hz, ^2^*J*_P,H_ = 20.3 Hz, 1H, *H*-Ir); −0.3-4.4 (26H, aliphatics from the piperidine ligand); 6.3–8.7 (56H, aromatics) ppm. ^31^P{^1^H} NMR (161.96 MHz, CDCl_3_): δ 21.05 (s, br); 22.96 (s, br); 26.97 (d, ^2^*J*_P,P_ = 12.6 Hz); 29.99 (d, ^2^*J*_P,P_ = 14.9 Hz) ppm. ^13^C{^1^H} NMR (100.61 MHz, CDCl_3_): δ 20–80 (aliphatics from the piperidine ligand); 122.0–162.0 (aromatics); 206.2 (d, ^2^*J*_P,C_ = 6 Hz, *C*=O); 211.2 (d, ^2^*J*_P,C_ = 6 Hz, *C*=O); 227.6 (d, ^2^*J*_P,C_ = 85 Hz, *C*=N); 231.0 (d, ^2^*J*_P,C_ = 95 Hz, *C*=N) ppm.

### Synthesis of Acyl-Iminium Type of Compounds [**4**]ClO_4_/[**3**]ClO_4_

The appropriate ketoimine type of complex **2** (0.03 mmol) was dissolved in 1.5 mL of THF to which another 1.5 mL of water was added. The solution was stirred for 24 h after which THF was removed under vacuum. The compounds were extracted with dichloromethane to which a solution of NaClO_4_ (0.03 mmol) in 2 mL of methanol was added. Light yellow precipitates of [**4a**]ClO_4_/[**3a**]ClO_4_ 80:20 mixtures (yield 63%) or of [**4b**]ClO_4_/[**3b**]ClO_4_ 60:40 mixtures (yield 45%) appeared, which were filtered and dried under vacuum.

#### *OC*-6-54—[IrH(PPh_2_(*o*-C_6_H_4_CO))(PPh_2_(*o*-C_6_H_4_C=N(*H*)CH_2_CH_2_NH_2_))]ClO_4_ ([**4a**]ClO_4_)

IR (KBr, cm^–1^): 3316 (w, N–H); 2165 (s, Ir–H); 1575 (s, C=O and C=N). Elemental Analysis for IrC_40_H_36_P_2_O_5_N_2_Cl·(CH_2_Cl_2_): Calculated: C 49.28, H 3.83, N 2.80. Found: C 49.56, H 3.62, N 2.76. ^1^H NMR (300 MHz, CDCl_3_): δ −17.62 (t, ^2^*J*_P,H_ = 16.5 Hz, 1H, *H*-Ir); 0.89 (m, 1H, *H*_2_C-NH_2_); 1.60 (m, 1H, N*H*_2_); 2.74 (m, 1H, *H*_2_C-NH_2_); 3.07 (m, 1H, N*H*_2_); 3.58 (m, 1H, *H*_2_C-NC); 3.76 (m, 1H, *H*_2_C-NC); 6.25–8.15 (28H, aromatics) ppm. ^31^P{^1^H} NMR (202.46 MHz, CDCl_3_): δ 10.8 (s); 32.4 (s) ppm. ^13^C{^1^H} NMR (125.76 MHz, CDCl_3_): δ 41.5 (s, *C*H_2_-NH_2_); 51.6 (s, H_2_*C*-NC); 121.0–162.0 (aromatics); 228.1 (d, ^2^*J*_P,C_ = 88 Hz, *C*=O or *C*=N); 235.2 (d, ^2^*J*_P,C_ = 93 Hz, *C*=O or *C*=N) ppm.

Single crystals of [**4a**]ClO_4_ were obtained by vapor diffusion of diethyl ether into a solution of [**4a**]ClO_4_ and [**3a**]ClO_4_ in methanol at −20 °C.

#### *OC*-6-54—[IrH(PPh_2_(*o*-C_6_H_4_CO))(PPh_2_(*o*-C_6_H_4_C=N(*H*)CH_2_CH_2_NHCH_3_))]ClO_4_ ([**4b**]ClO_4_)

IR (KBr, cm^–1^): 3224 (w, N–H); 2156 (s, Ir–H); 1604 and 1573 (s, C=O and C=N) and 1099 (s, Cl-O). Elemental Analysis for IrC_41_H_38_P_2_O_5_N_2_Cl: Calculated: C 53.04, H 4.13, N 3.02. Found: C 52.66, H 4.40, N 2.67. ^1^H NMR (300 MHz, CDCl_3_): δ *b*-19.84 (t, ^2^*J*_P,H_ = 18.3 Hz, 1H, *H*-Ir); *a* −19.63 (t, ^2^*J*_P,H_ = 17.8 Hz, 1H, *H*-Ir); 1.31 (m, 1H, *H*_2_C-NIr); 2.01 (m, 3H, *H*_3_C); 2.08 (m, 1H, *H*_2_C-NIr); 2.38 (m, 1H, *H*N-Ir), 3.90 (m, 1H, *H*_2_C-NC); 4.17 (m, 1H, *H*_2_C-NC); *b* 10.81 (s, 1H, *H*N-C); *a* 11.14 (s, 1H, *H*N-C); 6.25–8.15 (28H, aromatics) ppm. ^31^P{^1^H} NMR (165.95 MHz, CDCl_3_): δ *a* 6.6 (d, ^2^*J*_P,P_ = 11.7 Hz); *b* 11.5 (d, ^2^*J*_P,P_ = 11.5 Hz); *b* 28.9 (d, ^2^*J*_P,P_ = 11.5 Hz); *a* 33.8 (d, ^2^*J*_P,P_ = 12 Hz) ppm. ^13^C{^1^H} NMR (125.76 MHz, CDCl_3_): δ 45.6 (s, *C*H_3_); 48.3 (s, H_2_*C*-NC); 55.1 (s, H_2_*C*-NIr); 121.0–162.0 (aromatics) ppm.

### Synthesis of Acyl-Imine Type of Compounds **5**

KOH (0.075 mmol, 4.2 mg) was added to the appropriate solution of the acyl-iminium derivative (0.037 mmol) in methanol. The solution was stirred for 1 h, and then the solvent was evaporated in vacuum. The resulting solid was dissolved in dichloromethane and extracted with water. The organic solvent was removed under vacuum to afford a yellow solid which was washed with diethyl ether and hexane.

#### *OC*-6-56—[IrH(PPh_2_(*o*-C_6_H_4_CO))(PPh_2_(*o*-C_6_H_4_C=NCH_2_CH_2_NH_2_))] (**5a**)

Yield 71%. IR (KBr, cm^–1^): 3318 and 3356 (w, N–H); 2011 (s, Ir–H); 1601 (s, C=O and C=N). Elemental Analysis for IrC_40_H_35_P_2_ON_2_·(CH_2_Cl_2_): Calculated: C 54.79, H 4.15, N 3.12. Found: C 54.81, H 4.18, N 2.70. ^1^H NMR (400 MHz, CDCl_3_): δ −8.53 (dd, ^2^*J*_P,H_ = 122.0 Hz, ^2^*J*_P,H_ = 18.2 Hz, 1H, *H*-Ir); 1.25 (m, 1H, *H*_2_C-NH_2_); 2.35 (m, 1H, *H*_2_C-NH_2_); 3.07 (m, 2H, N*H*_2_); 3.54 (m, 1H, *H*_2_C-NC); 4.23 (m, 1H, *H*_2_C-NC); 6.5–8.3 (28H, aromatics) ppm. ^31^P{^1^H} NMR (161.96 MHz, CDCl_3_): δ 25.6 (s, br); 27.3 (d, ^2^*J*_P,P_ = 7.1 Hz) ppm. ^13^C{^1^H} NMR (100.61 MHz, CDCl_3_): δ 38.1 (s, *C*H_2_-NH_2_); 64.54 (s, H_2_*C*-NC); 122.0–164 (aromatics); 208.3 (d, ^2^*J*_P,C_ = 80.3 Hz, *C*=N); 214.8 (d, ^2^*J*_P,C_ = 6.8 Hz *C*=O) ppm.

#### *OC*-6-56—[IrH(PPh_2_(*o*-C_6_H_4_CO))(PPh_2_(*o*-C_6_H_4_C=NCH_2_CH_2_NHCH_3_))] (**5b**)

Yield 65%. IR (KBr, cm^–1^): 3281 (w, N–H); 2009 (s, Ir–H); 1600 (s, C=O and C=N). Elemental Analysis for IrC_41_H_37_P_2_ON_2_·(CH_2_Cl_2_)_0.75_: Calculated: C 56.24, H 4.35, N 3.14. Found: C 56.01, H 4.04, N 2.97. ^1^H NMR (500 MHz, CDCl_3_): δ −9.02 (dd, ^2^*J*_P,H_ = 124.4 Hz, ^2^*J*_P,H_ = 18.5 Hz, 1H, *H*-Ir); −8.35 (dd, ^2^*J*_P,H_ = 123.2 Hz, ^2^*J*_P,H_ = 19.7 Hz, 1H, *H*-Ir); 1.15 (m, 1H, N*H*); 2.19 (m, 1H, *H*_2_C-NH); 2.27 (d, ^3^*J*_H,H_ = 6.3 Hz, 3H, *H*_3_C); 2.33 (m, 1H, *H*_2_C-NH); 3.83 (td, ^2^*J*_H,H_ = 11.2 Hz, ^3^*J*_H,H_ = 6.6 Hz, 1H, *H*_2_C-NC); 4.32 (dd, ^2^*J*_H,H_ = 11.7 Hz, ^3^*J*_H,H_ = 4.9 Hz, 1H, *H*_2_C-NC); 6.4–8.4 (28H, aromatics) ppm. ^31^P NMR (161.96 MHz, CDCl_3_): δ 24.8 (d, ^2^*J*_H,P_ = 123 Hz); 26.7 (s) ppm. ^13^C{^1^H} NMR (125.76 MHz, CDCl_3_): δ 46.8 (d, ^2^*J*_P,C_ = 5.3 Hz, *C*H_3_); 51.9 (d, ^2^*J*_P,C_ = 5.1 Hz, *C*H_2_-NH_2_); 62.3 (s, H_2_*C*-NC); 122.0–163.0 (aromatics); 213.5 (s, *C*=O) ppm.

Yellow single crystals of **5b** were obtained by vapor diffusion of diethyl ether into a solution of **5b** in chloroform at −20 °C.

#### *OC*-6-56—[IrH(PPh_2_(*o*-C_6_H_4_CO))(PPh_2_(*o*-C_6_H_4_C=NCH_2_CH_2_CH_2_NH_2_))] (**5d**)

Yield 68%. IR (KBr, cm^–1^): 3312 and 3244 (w, N–H); 2027 (s, Ir–H); 1559 (s, C=O and C=N). Elemental Analysis for IrC_41_H_37_P_2_ON_2_·(CH_2_Cl_2_)_0.7_: Calculated: C 56.85, H 4.38, N 3.19. Found: C 56.70, H 4.82, N 3.00. ^1^H NMR (400 MHz, CDCl_3_): δ −7.74 (dd, ^2^*J*_P,H_ = 125.2 Hz, ^2^*J*_P,H_ = 18.3 Hz, 1H, *H*-Ir); 1.66 (m, 1H, *H*_2_C-NH_2_); 1.69 (m, 1H, CH_2_-*H*_2_C-CH_2_); 1.88 (m, 1H, CH_2_-*H*_2_C-CH_2_); 2.20 (m, 1H, *H*_2_C-NH_2_); 4.32 (m, 1H, *H*_2_C-NC); 4.48 (m, 1H, *H*_2_C-NC); 6.2–8.5 (28H, aromatics) ppm. ^31^P{^1^H} NMR (161.96 MHz, CDCl_3_): δ 25.9 (s, br); 27.5 (d, ^2^*J*_P,P_ = 6.4 Hz) ppm. ^13^C{^1^H} NMR (100.61 MHz, CDCl_3_): δ 28.5 (s, CH_2_-*C*H_2_-CH_2_); 42.3 (s, H_2_*C*-NH_2_); 58.1 (s, H_2_*C*-NC); 122.0–163 (aromatics); 213.1 (s, *C*=O) ppm.

## Supporting Information Available

X-ray diffraction tables, original spectra of all compounds, ^1^H and ^11^B NMR of the in situ methanolysis, and plots for the catalysis of **2d** (PDF)
