# Supplementary material for: Formation of Irida-β-ketoimines and PCNamine-Ir(III) Complexes by Reacting Irida-β-diketones with Aliphatic Diamines: Catalytic Activity in Hydrogen Release by Methanolysis of H3N–BH3
Source: Organometallics. 2022 Dec 1;41(23):3654–63. doi: 10.1021/acs.organomet.2c00451 (PMC10407880; doi:10.1021/acs.organomet.2c00451)
Supplement: Supplementary file 1 — om2c00451_si_001.pdf [file om2c00451_si_001.pdf]

Supporting information for:

Formation of Irida-β-ketoimines and PCN^{amine}-Ir(III) complexes by reacting irida-β-diketones with aliphatic diamines. Catalytic activity in hydrogen release by methanolysis of $H_3N–BH_3$.

Itxaso Bustos[a], Jose M. Seco[a], Antonio Rodriguez-Dieguez[b], María A. Garralda*[a], Claudio Mendicute-Fierro*[a]

a Department of Applied Chemistry, Faculty of Chemistry, University of The Basque Country UPV/EHU, Paseo Manuel Lardizabal 3, 20018, Donostia-San Sebastián, Spain.

b Departament of Inorganic Chemistry, Faculty of Science, University of Granada, 18071, Granada, Spain.

Email addresses for corresponding authors: mariaangeles.garralda@ehu.eus; claudio.mendiucte@ehu.eus

Table of contents

Characterisation of compounds 2

Compound 2a 2

Compound 2b 5

Compound 2c 8

Compound 2d 11

Compound 2e 14

Compound 3a 17

Compound 3b 20

Compound 3d 23

Compound 3e 26

Compound 4a 29

Compound 4b 32

Compound 5a 35

Compound 5b 38

Compound 5d 41

X-Ray diffraction data 45

Catalytic studies 46

Characterisation of compounds

Compound 2a

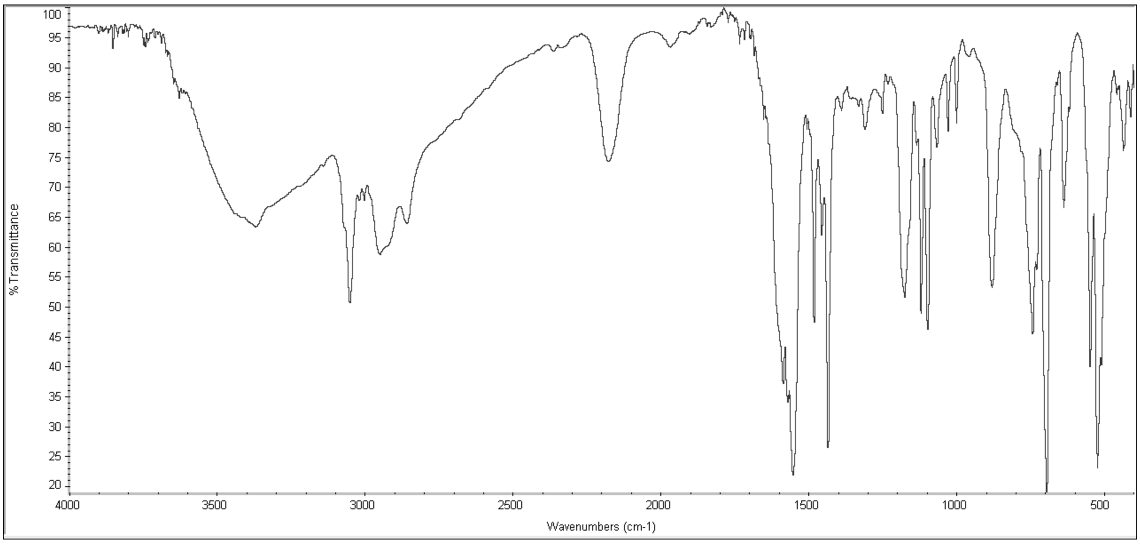

Figure S1 IR Spectrum of complex 2a.

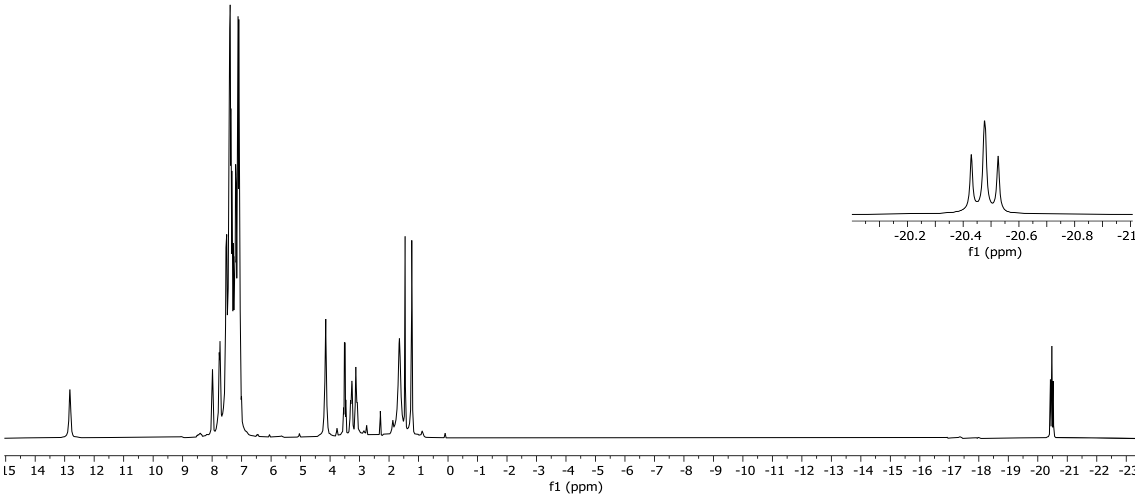

Figure S2 ^{1}H NMR of complex 2a in $CDCl_3$

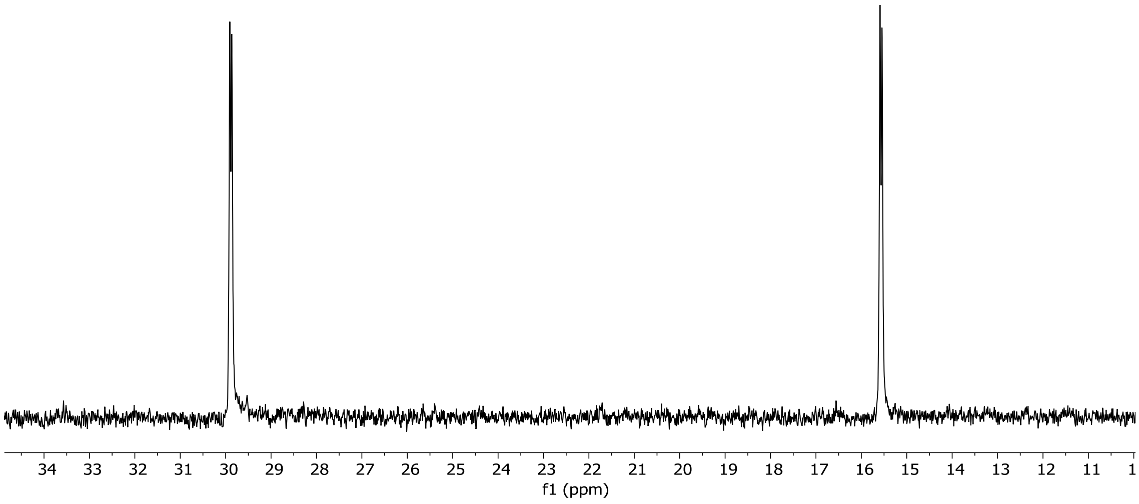

Figure S3 $^{31}P\{^{1}H\}$ NMR of complex 2a in $CDCl_3$

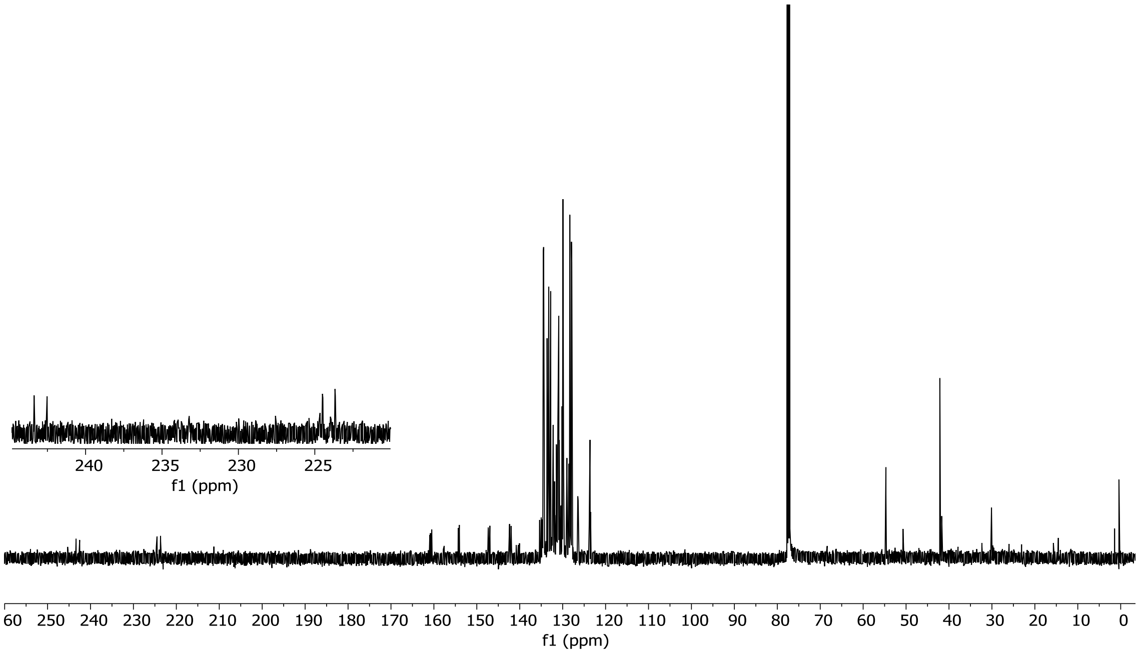

Figure S4 $^{13}C\{^{1}H\}$ NMR of complex 2a in $CDCl_3$

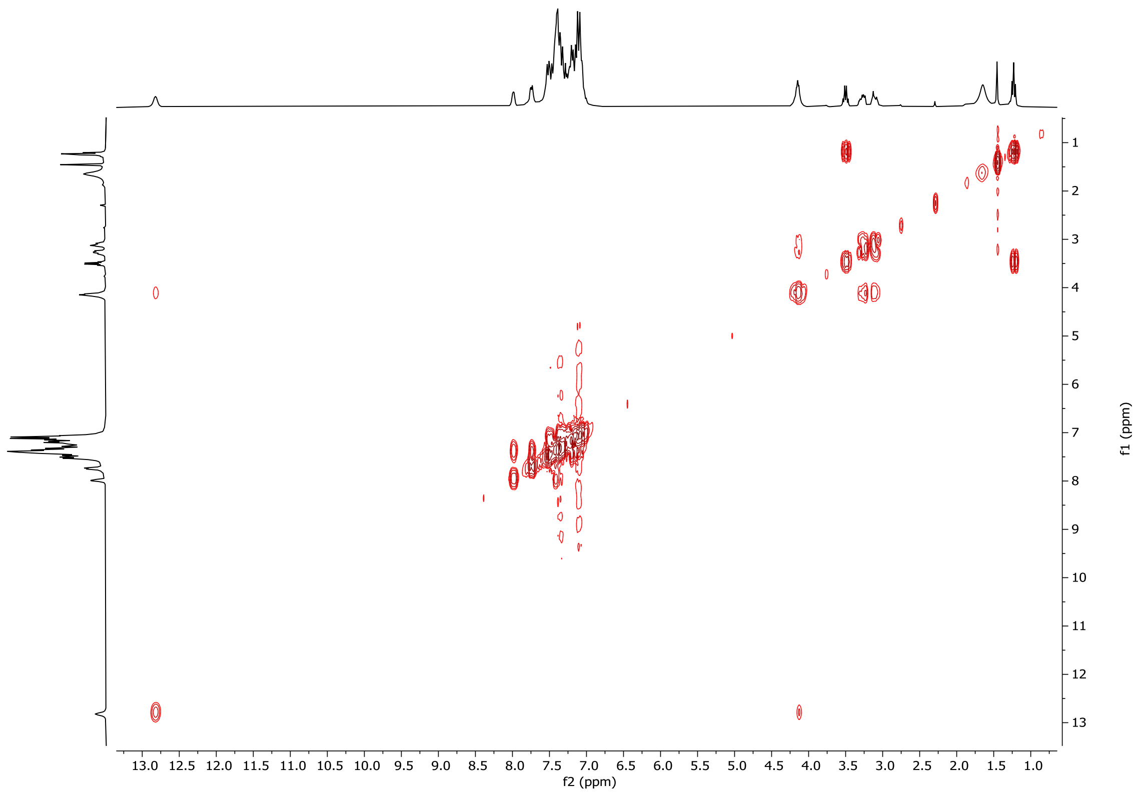

Figure S5 COSY spectrum of complex 2a in $CDCl_3$

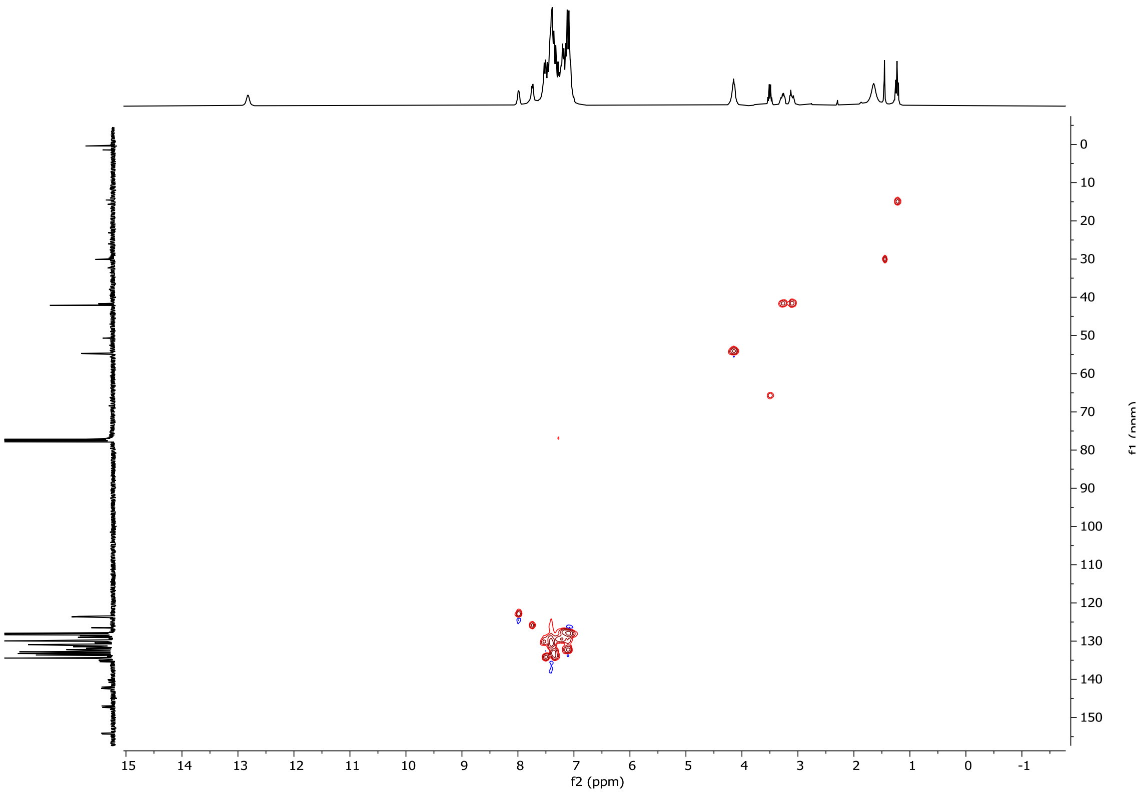

Figure S6 ^{1}H-^{13}C HSQC spectrum of complex 2a in $CDCl_3$

Compound 2b

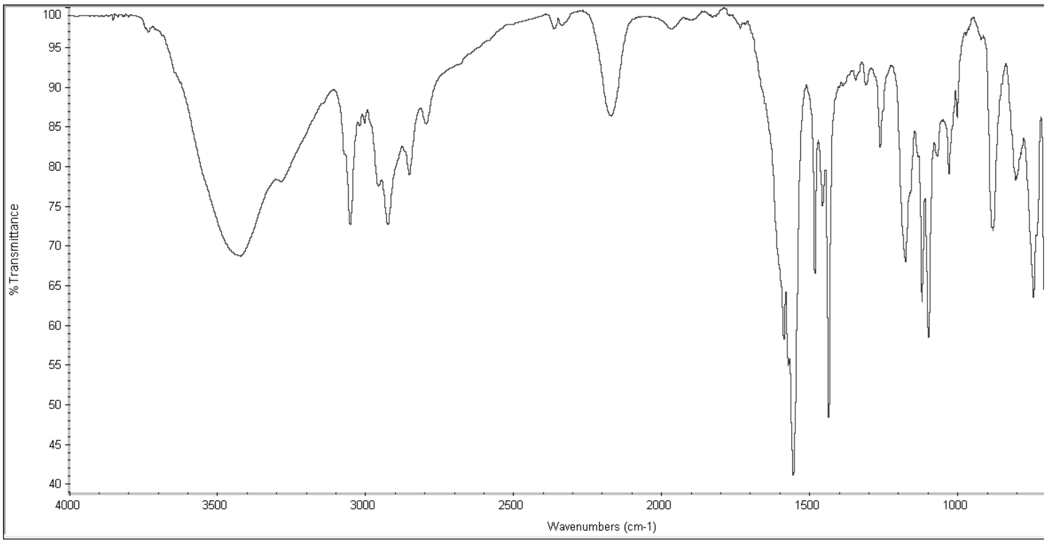

Figure S7 IR Spectrum of complex 2b

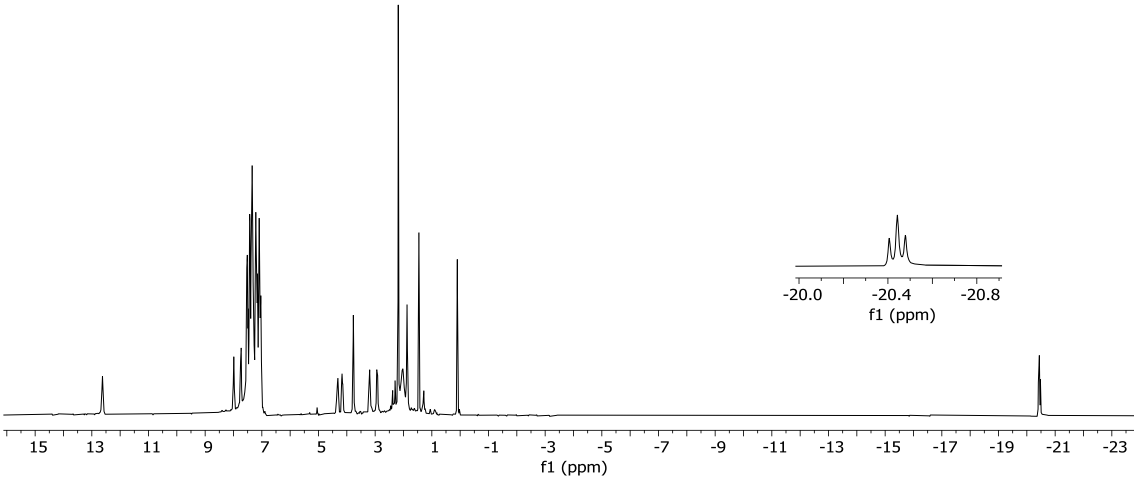

Figure S8 ^{1}H NMR of complex 2b in $CDCl_3$

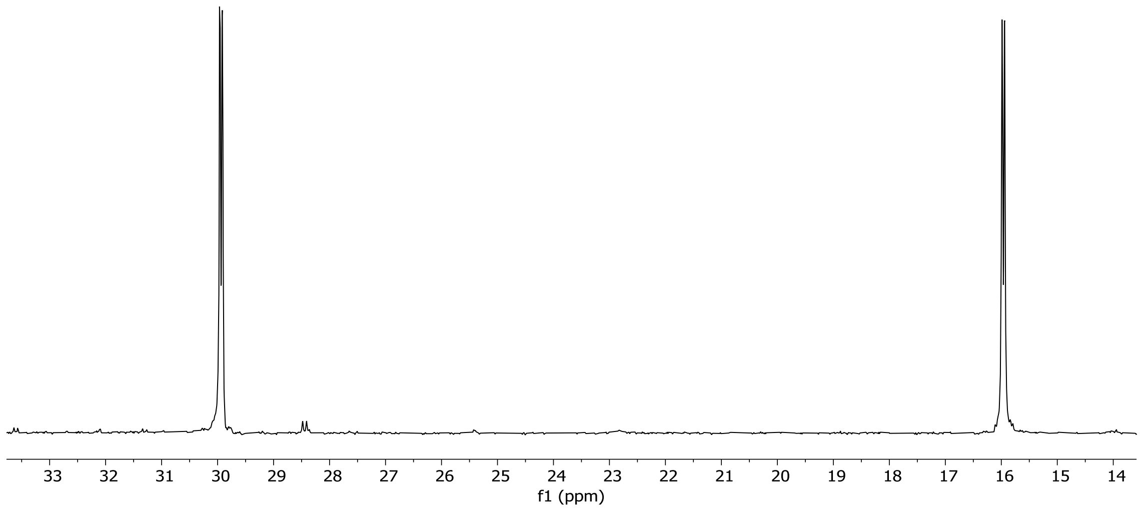

Figure S9 $^{31}P\{^{1}H\}$ NMR of complex 2b in $CDCl_3$

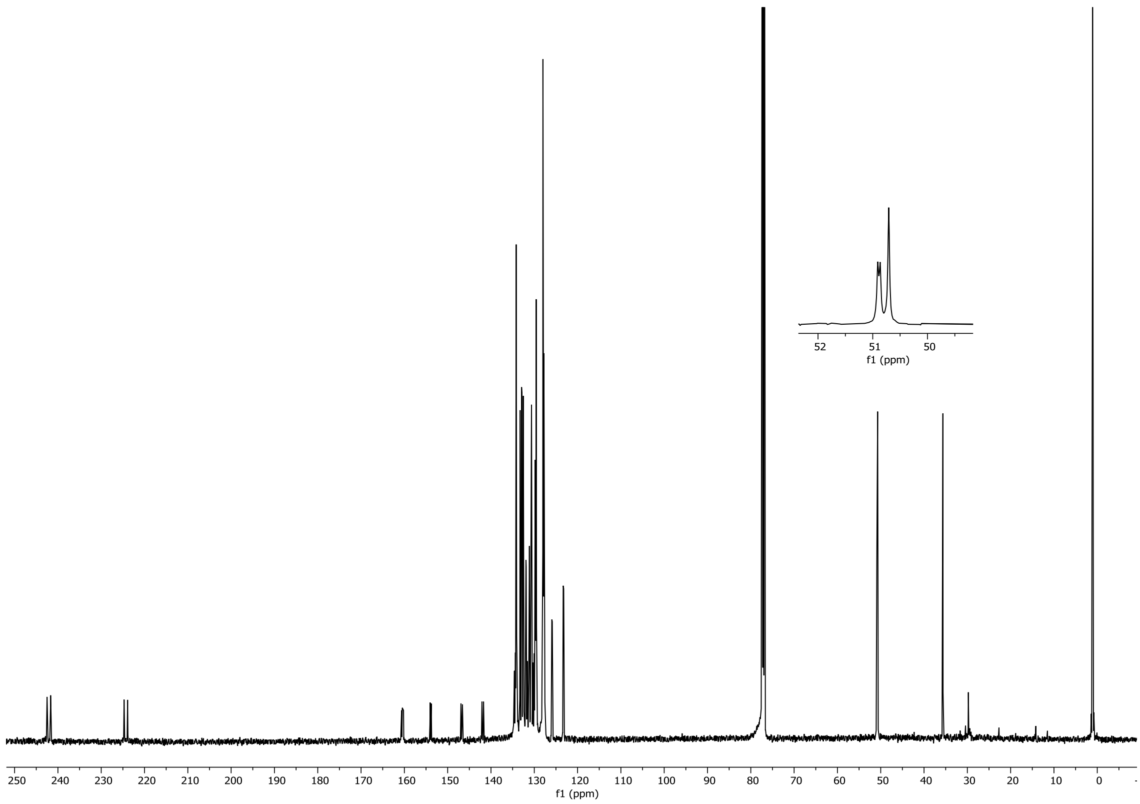

Figure S10 $^{13}C\{^{1}H\}$ NMR of complex 2b in $CDCl_3$

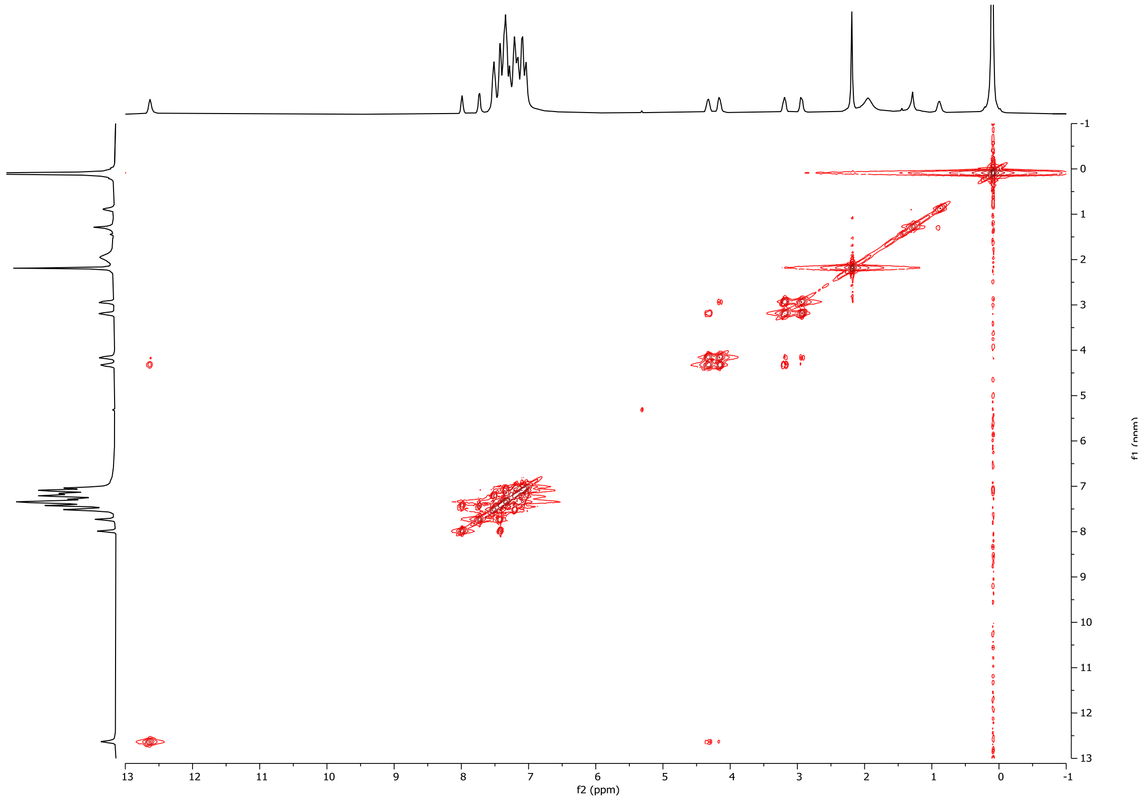

Figure S11 COSY spectrum of complex 2b in $CDCl_3$

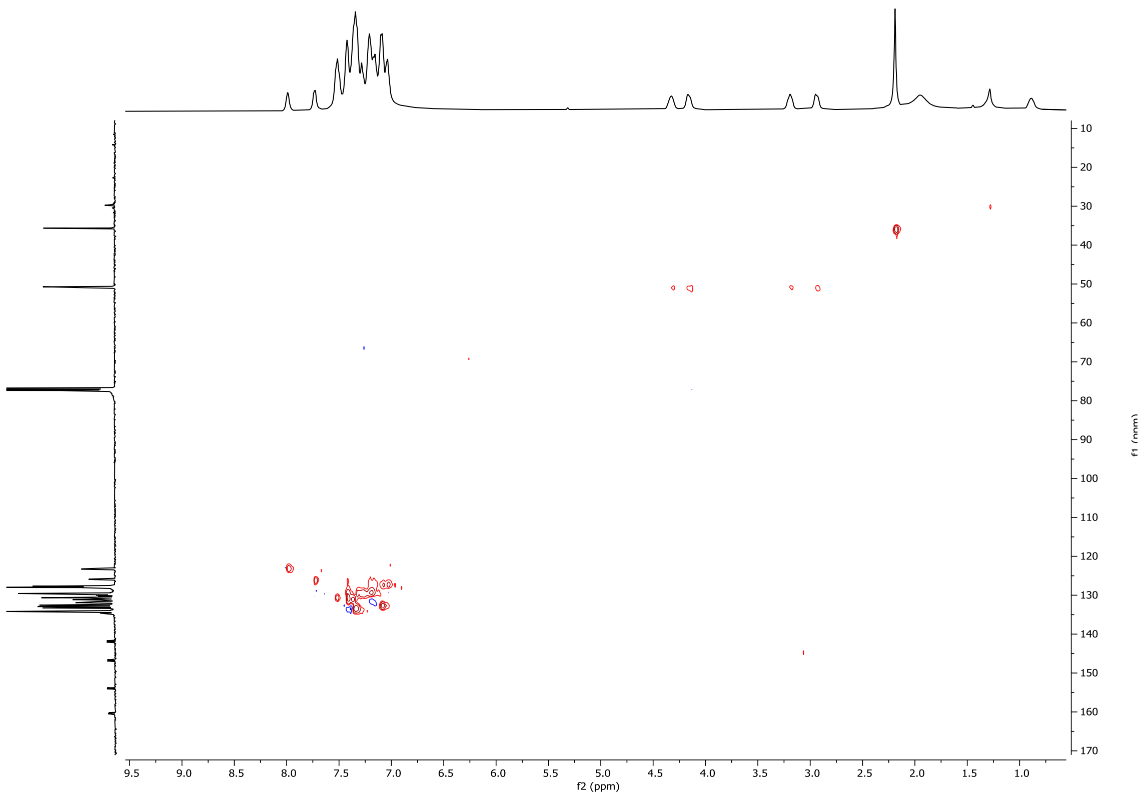

Figure S12 ^1H-^{13}C HSQC spectrum of complex 2b in $CDCl_3$

Compound 2c

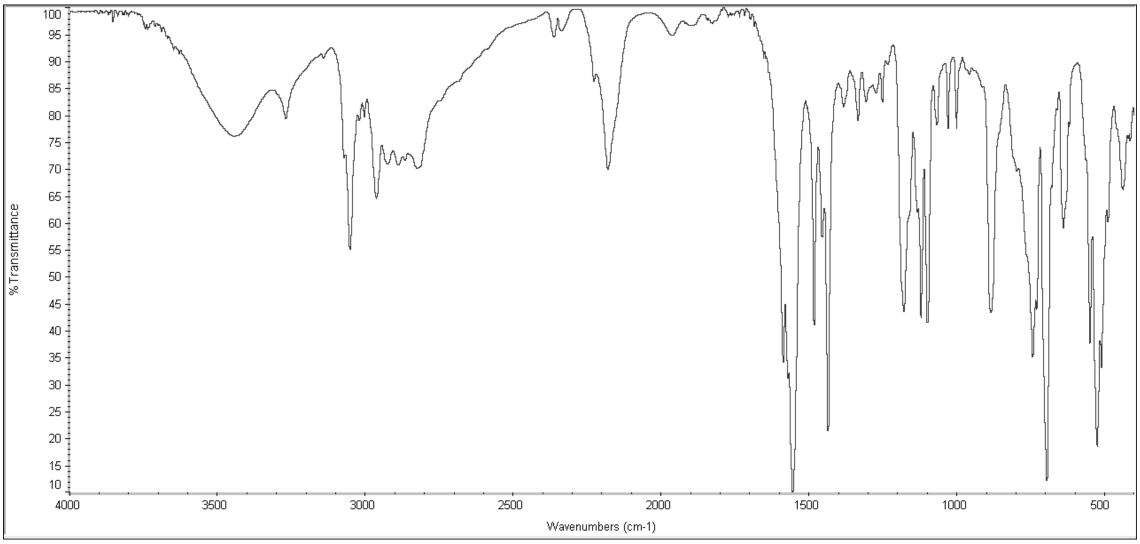

Figure S13 IR Spectrum of complex 2c

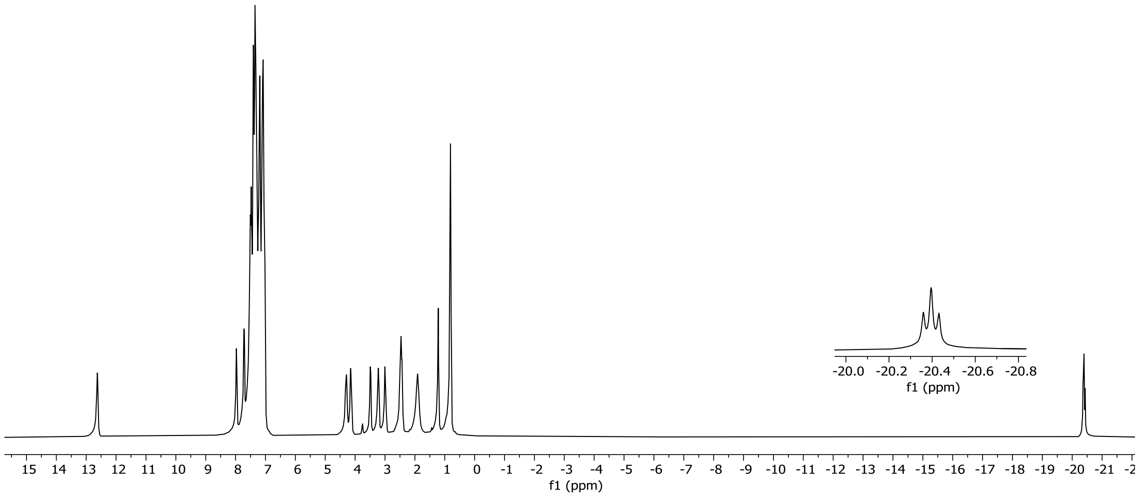

Figure S14 ^{1}H NMR of complex 2c in $CDCl_3$

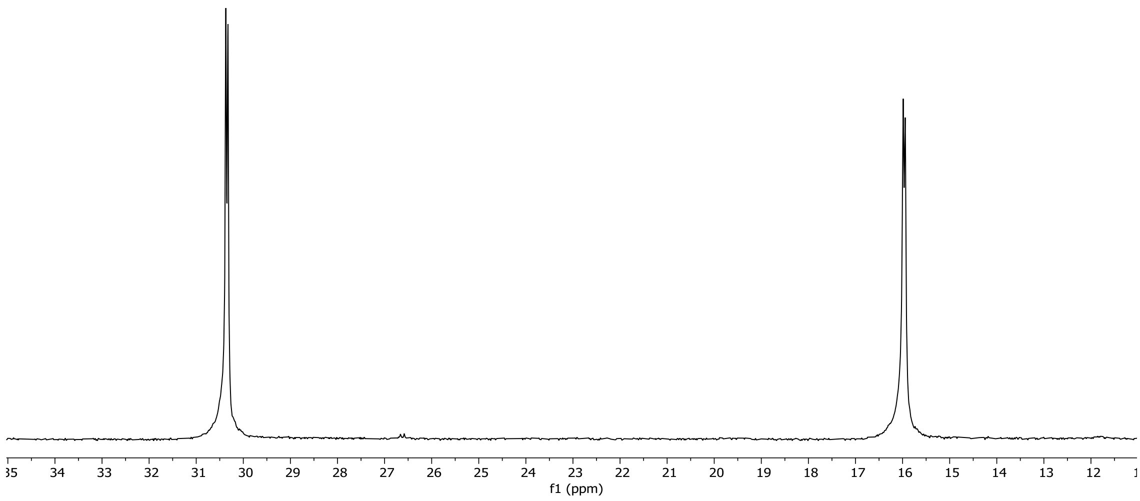

Figure S15 $^{31}P\{^1H\}$ NMR of complex 2c in $CDCl_3$

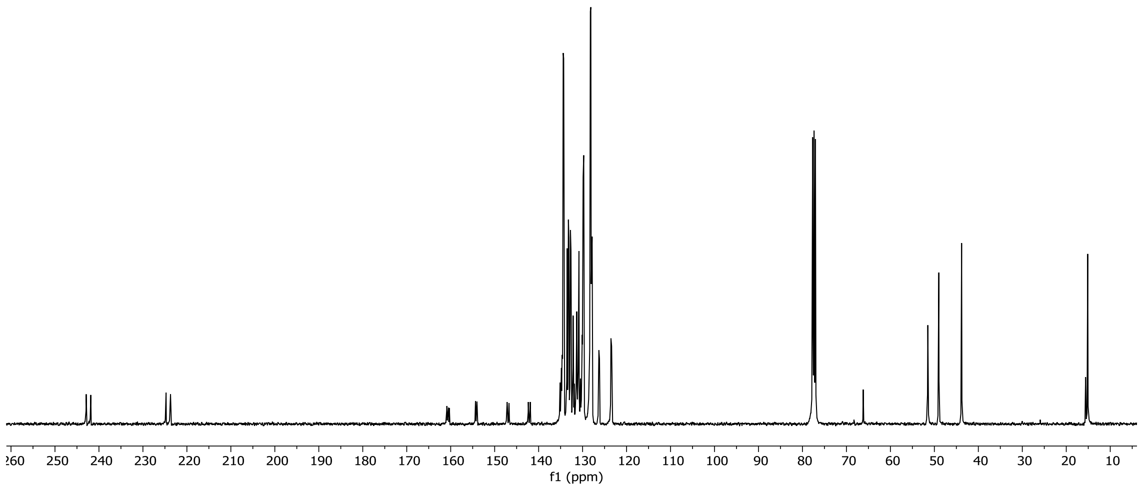

Figure S16 $^{13}C\{^1H\}$ NMR of complex 2c in $CDCl_3$

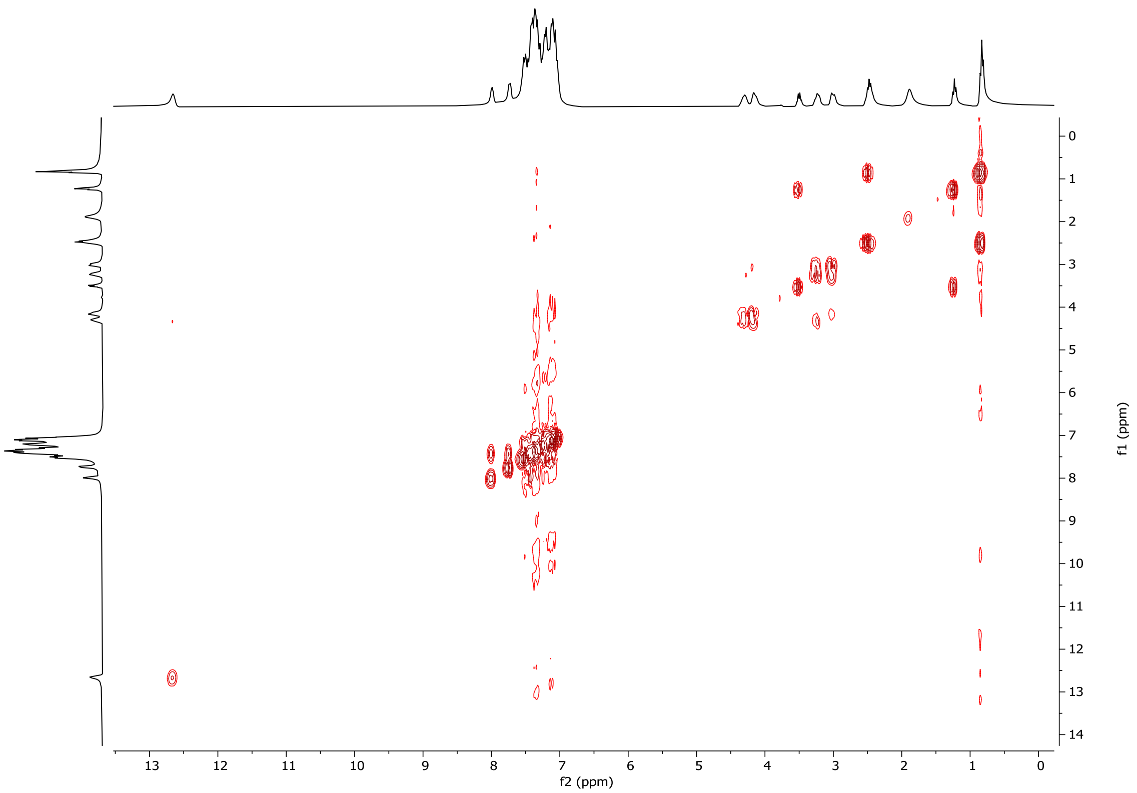

Figure S17 COSY spectrum of complex 2c in $CDCl_3$

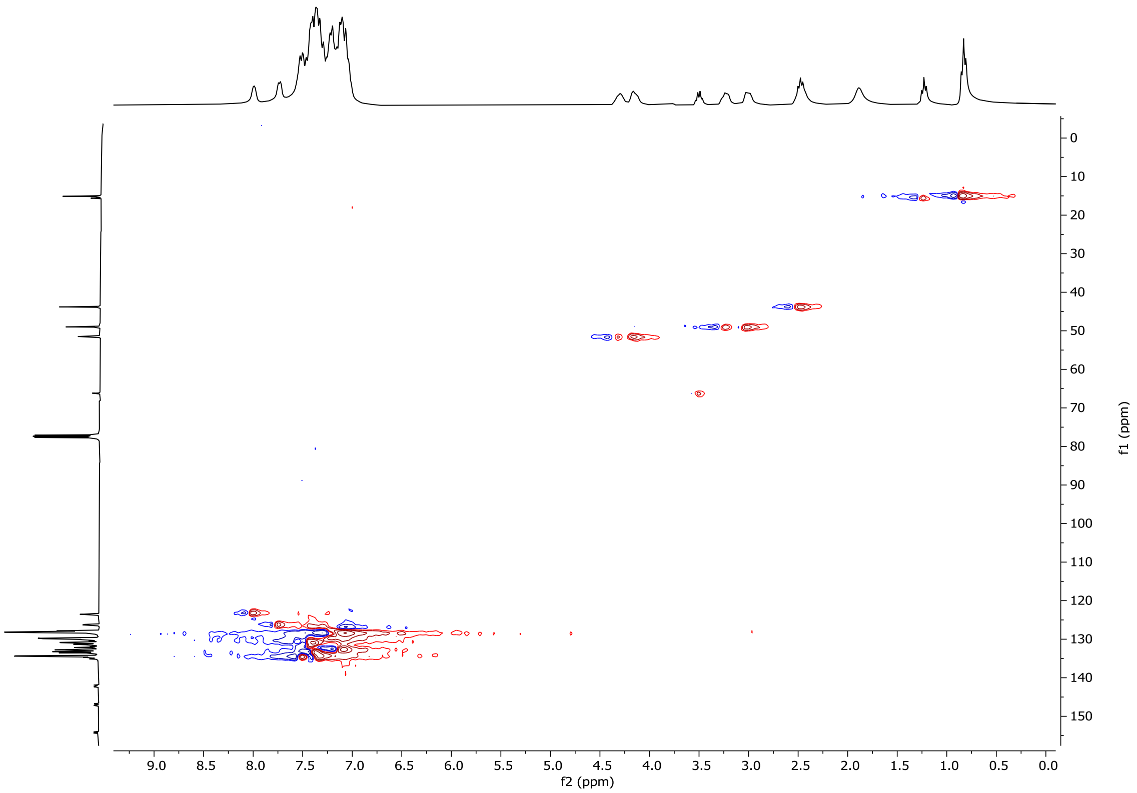

Figure S18 ^{1}H-^{13}C HSQC spectrum of complex 2c in $CDCl_3$

Compound 2d

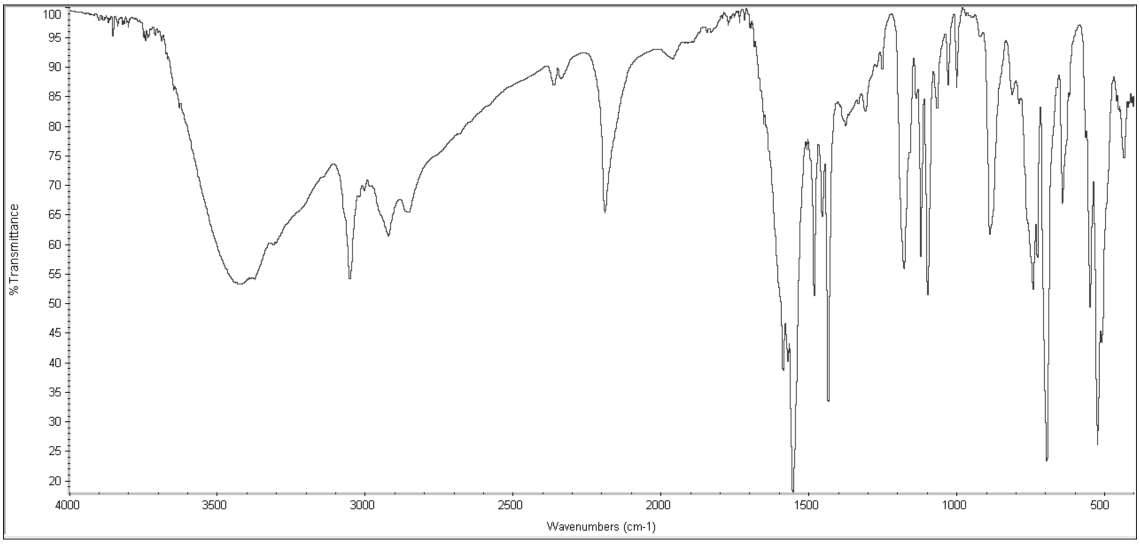

Figure S19 IR Spectrum of complex 2d

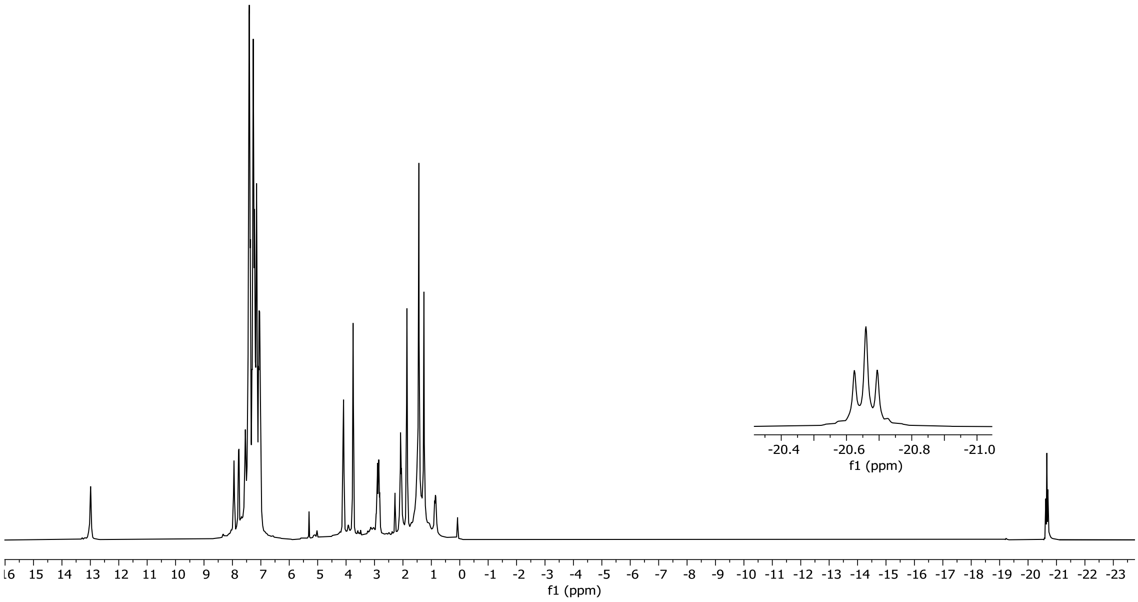

Figure S20 ^{1}H NMR of complex 2d in $CDCl_3$

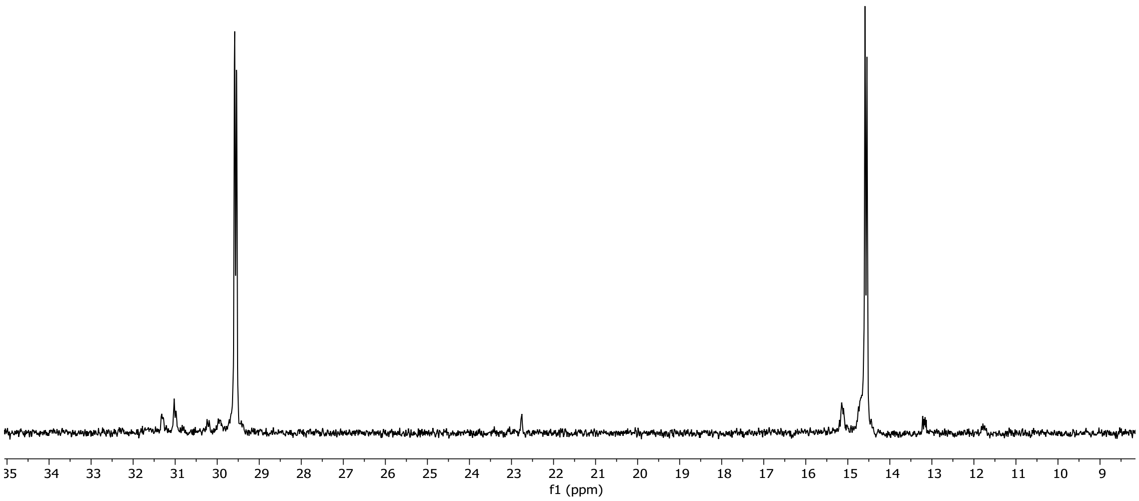

Figuree 21 $^{31}P\{^{1}H\}$ NMR of complex 2d in $CDCl_3$

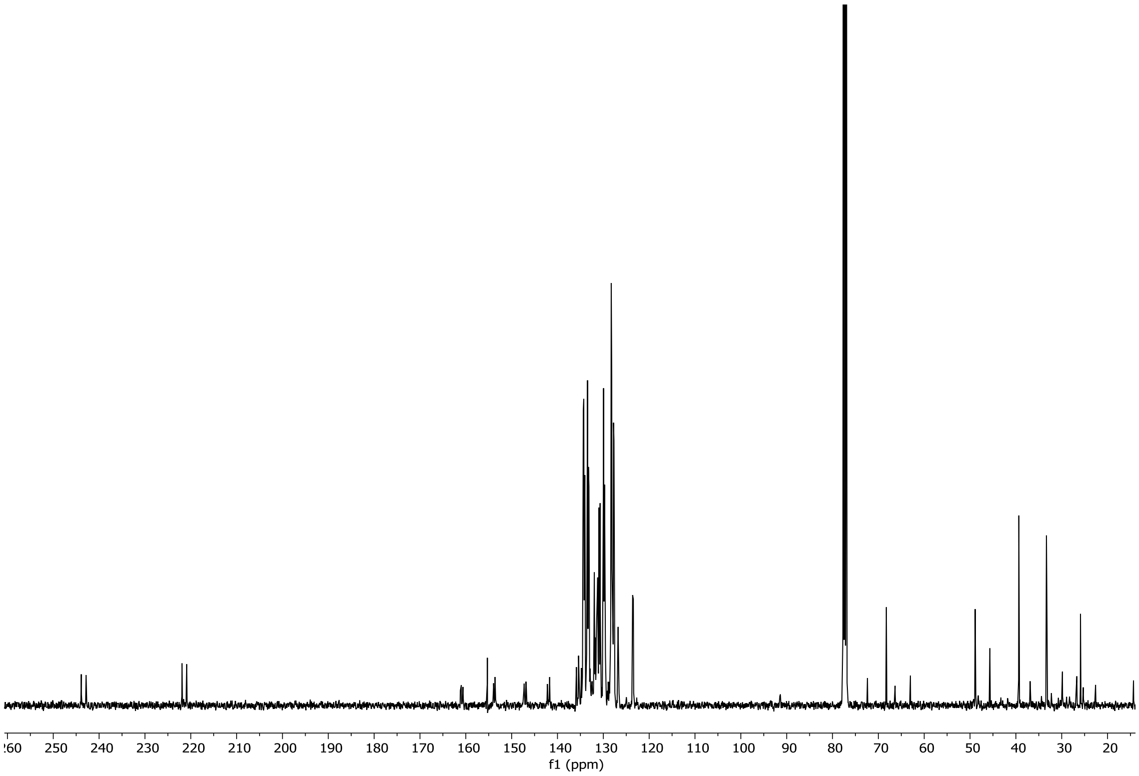

Figure S22 $^{13}C\{^{1}H\}$ NMR of complex 2d in $CDCl_3$

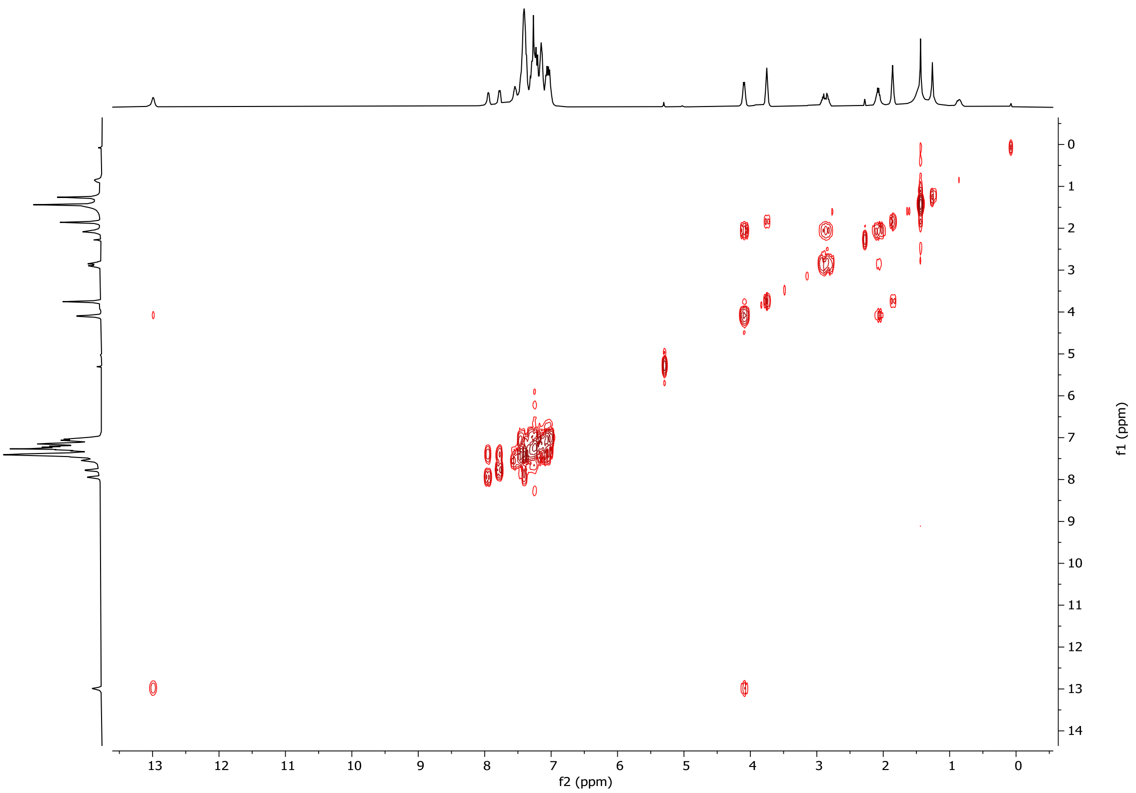

Figure S23 COSY spectrum of complex 2d in $CDCl_3$

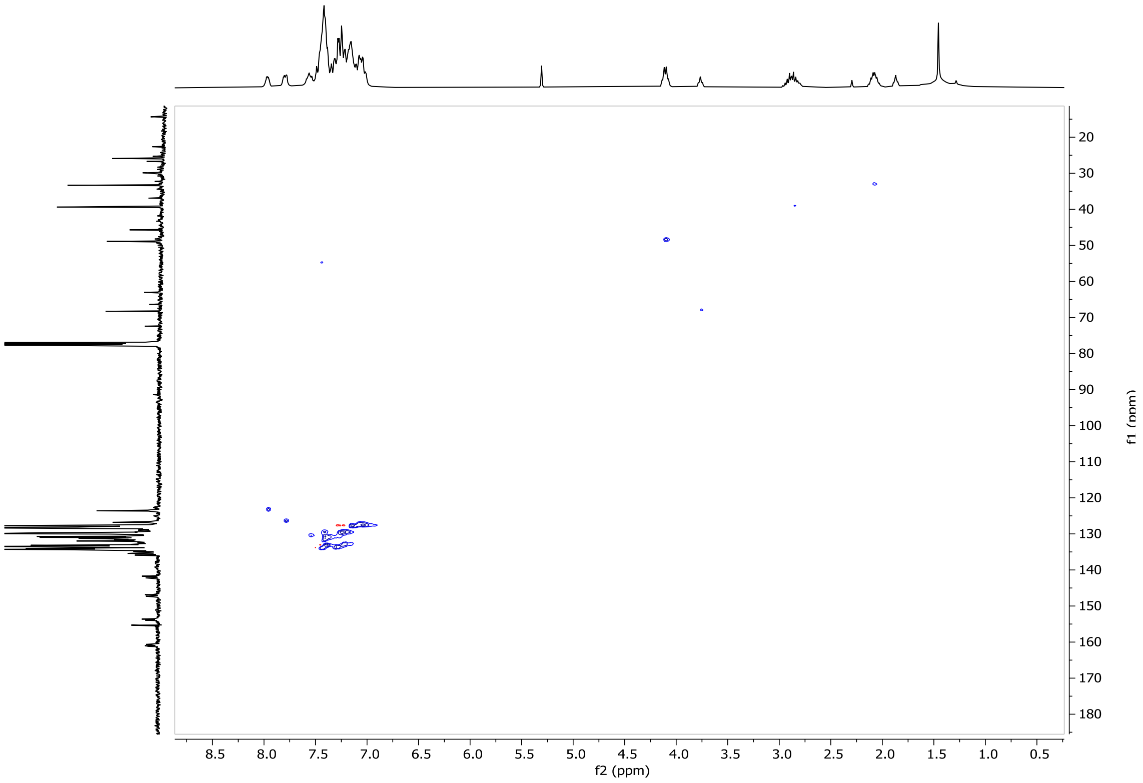

Figure S24 ^{1}H-^{13}C HSQC spectrum of complex 2d in $CDCl_3$

Compound 2e

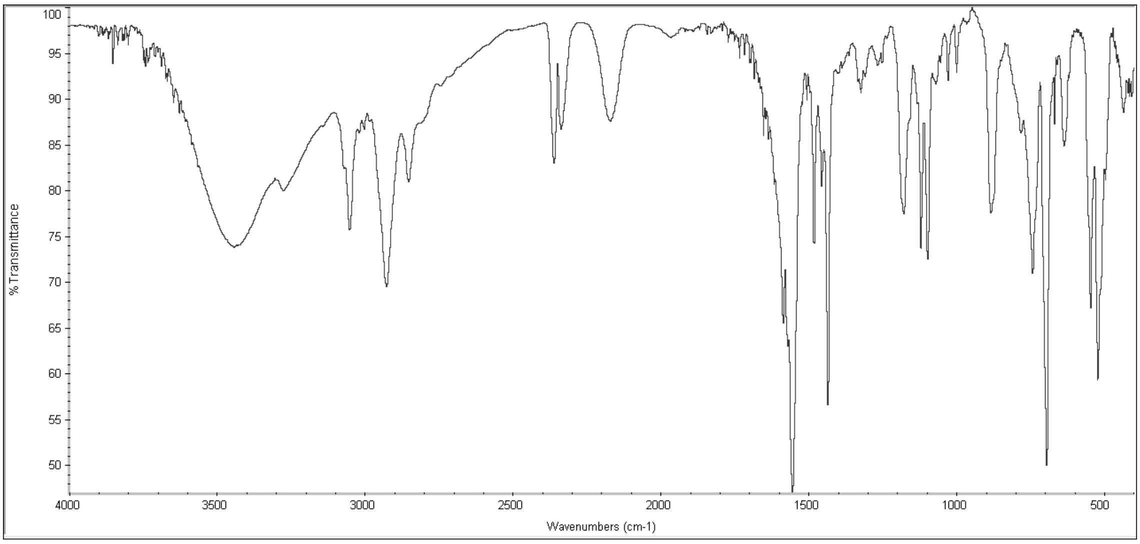

Figure S25 IR Spectrum of complex 2e

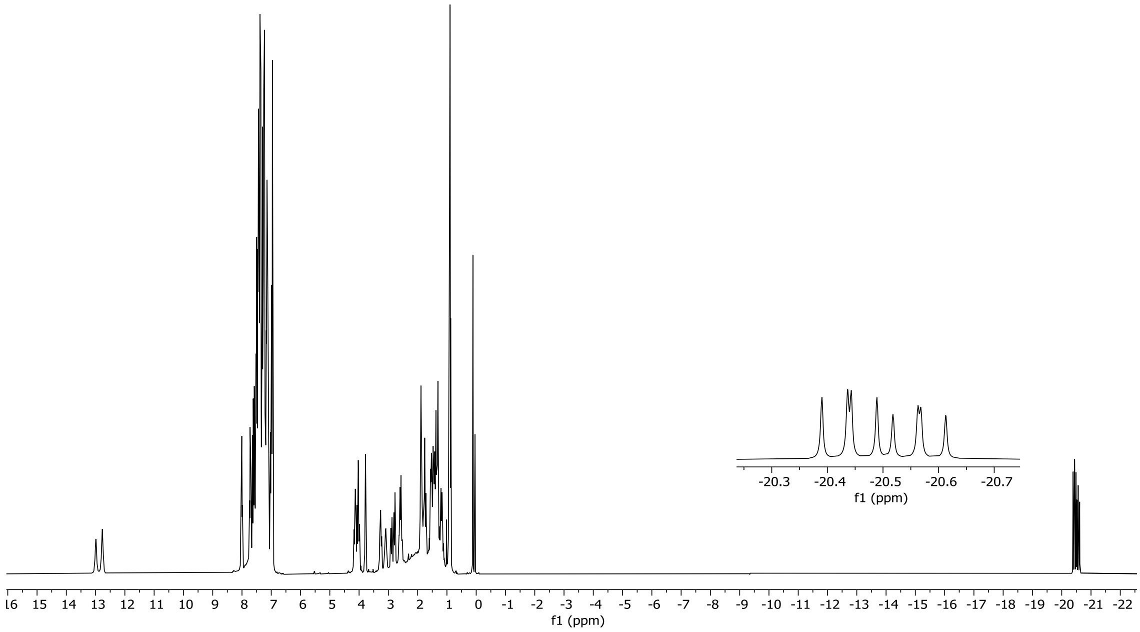

Figure S26 ^{1}H NMR of complex 2e in $CDCl_3$

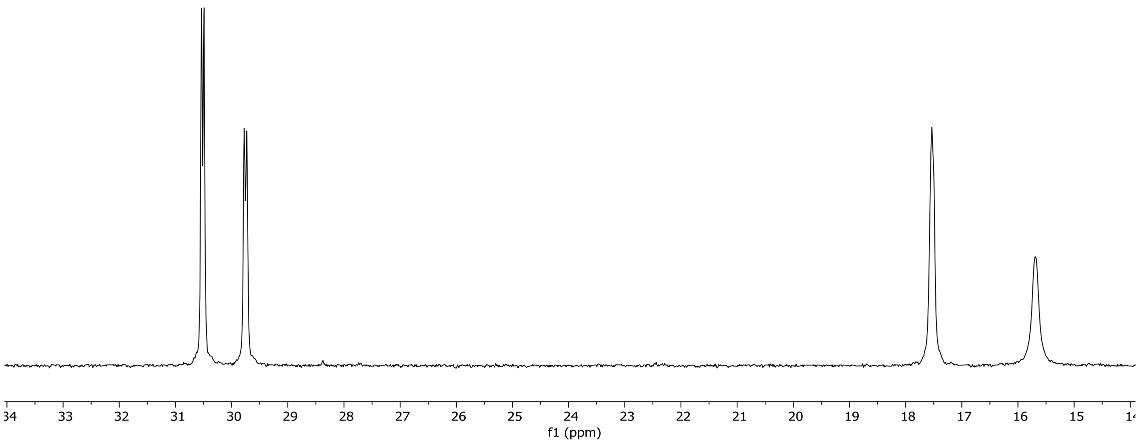

Figure S27 $^{31}P\{^{1}H\}$ NMR of complex 2e in $CDCl_3$

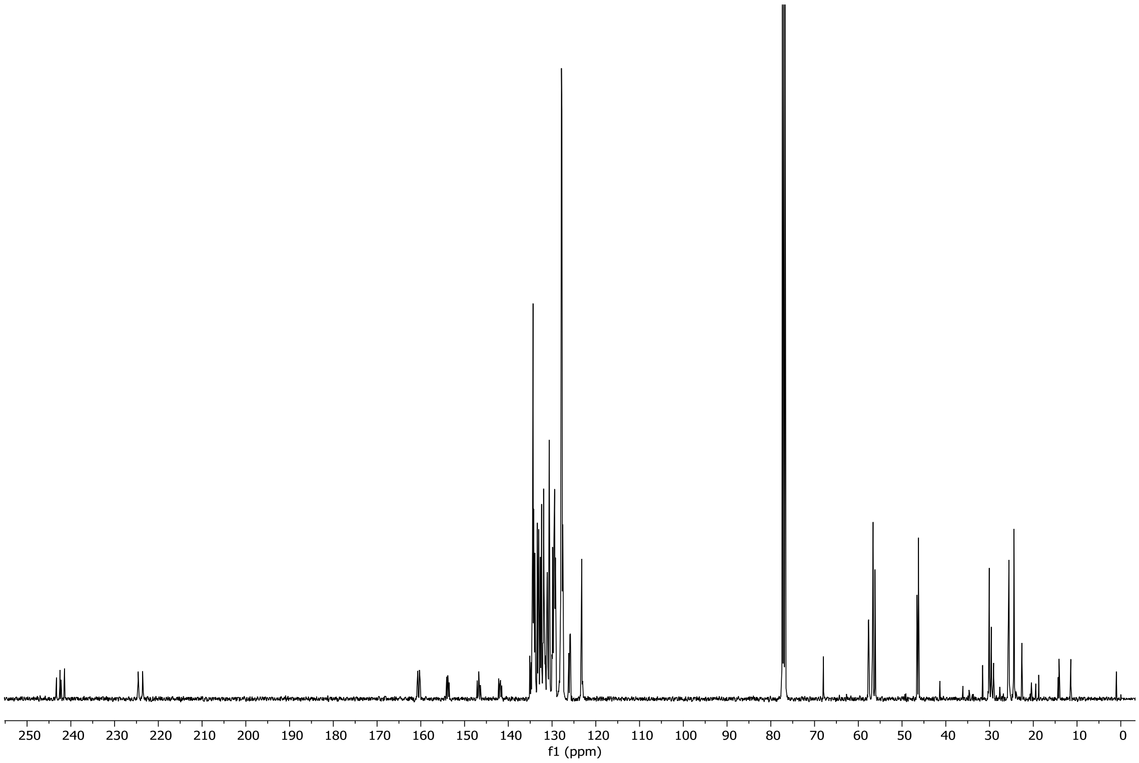

Figure S28 $^{13}C\{^{1}H\}$ NMR of complex 2e in $CDCl_3$

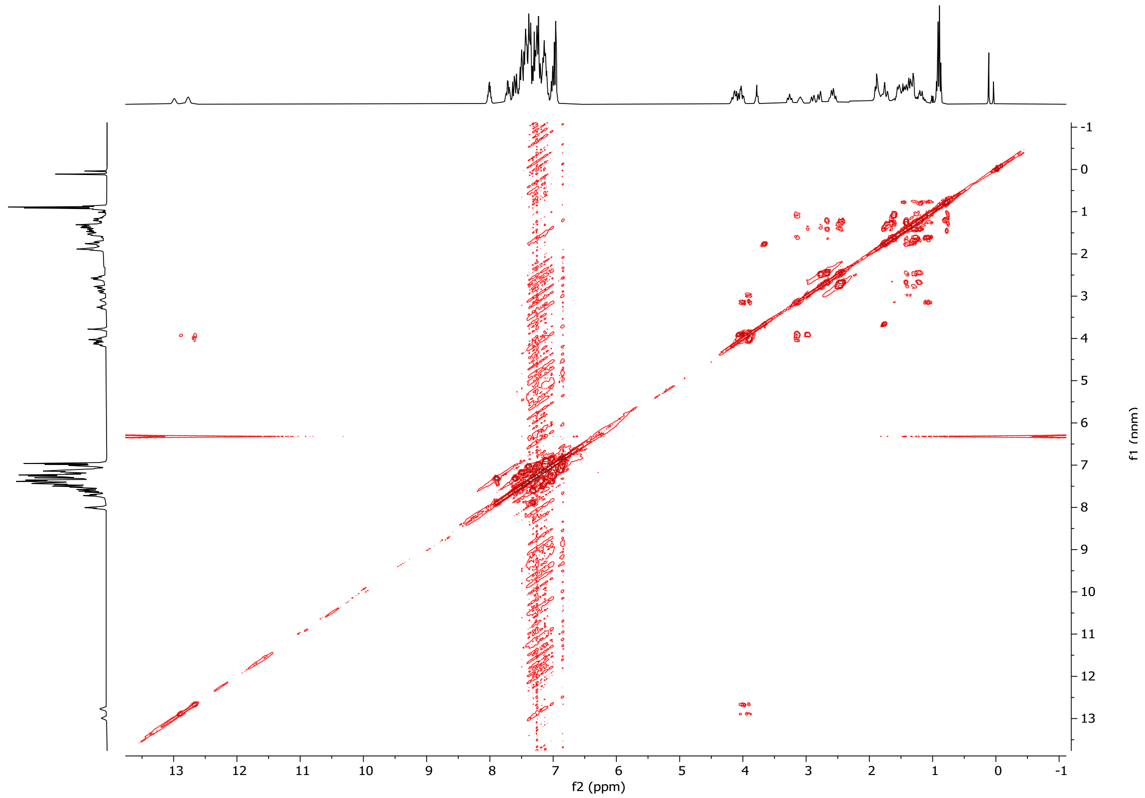

Figure S29 COSY spectrum of complex 2e in $CDCl_3$

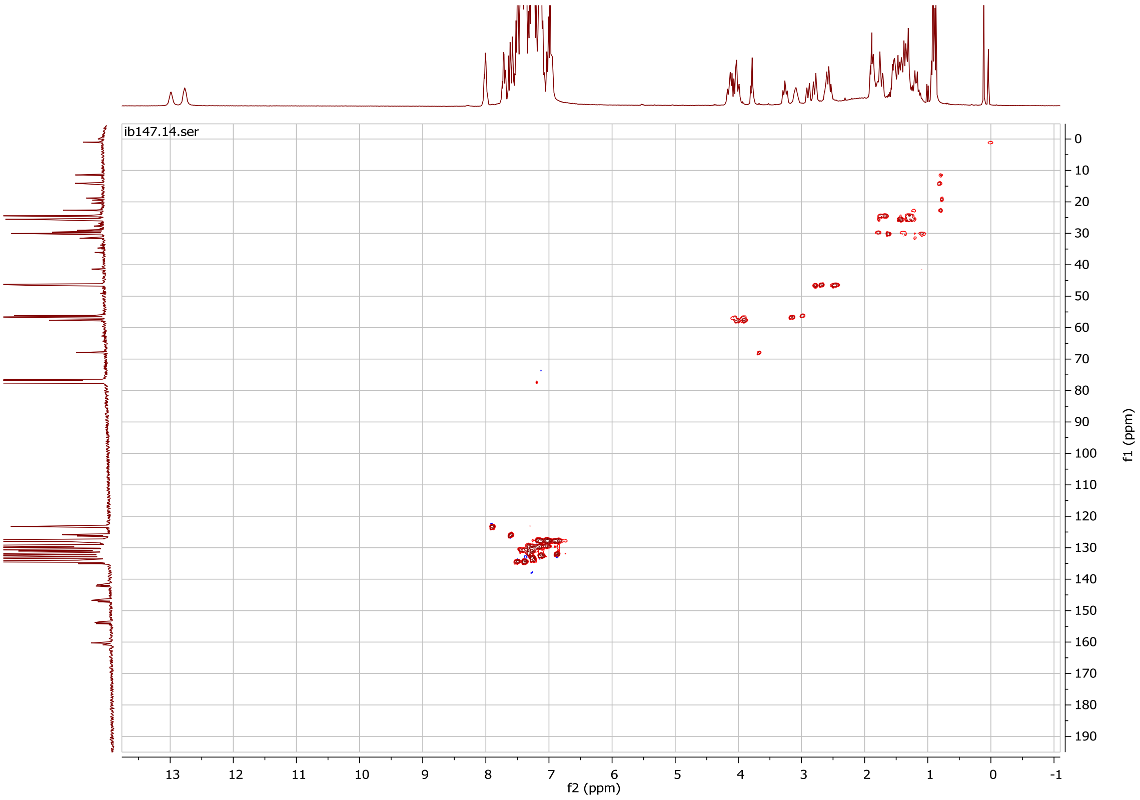

Figure S30 ^{1}H-^{13}C HSQC spectrum of complex 2e in $CDCl_3$

Compound 3a

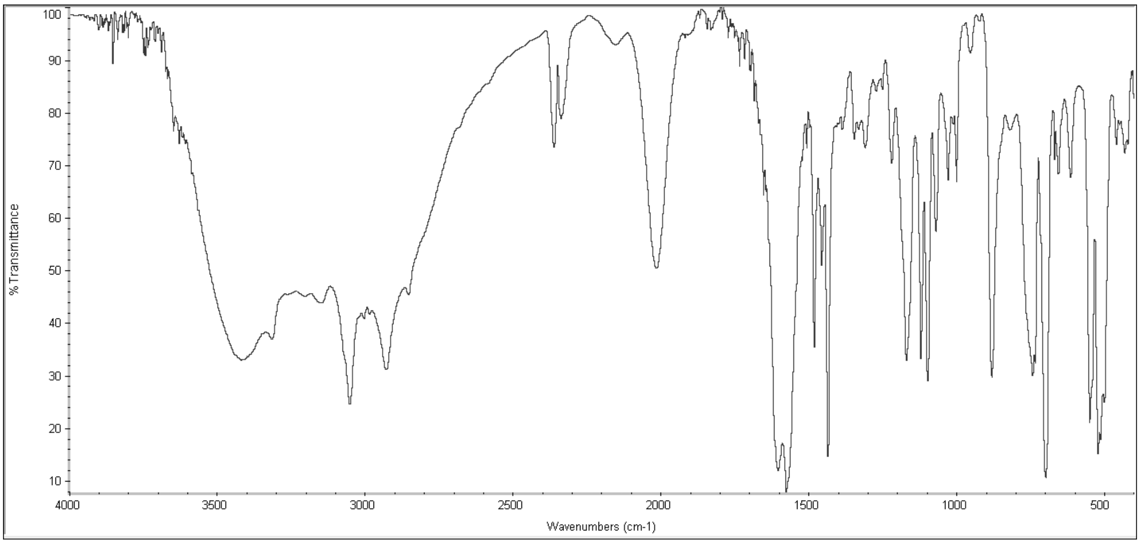

Figure S31 IR Spectrum of complex 3a

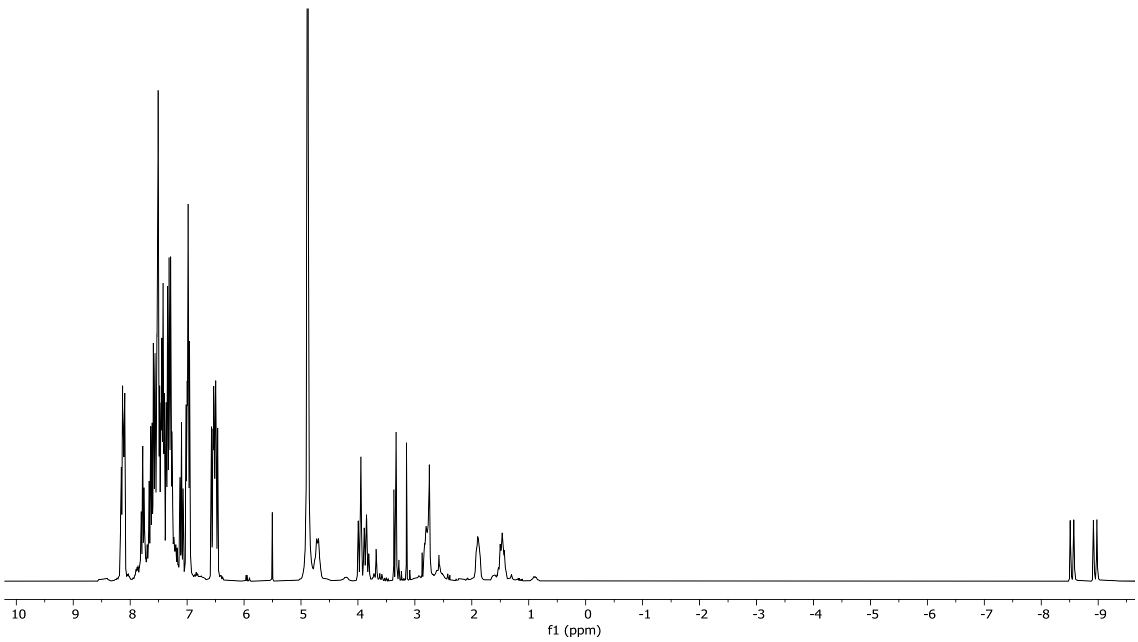

Figure S32 ^1H NMR of complex 3a in CD_3OD

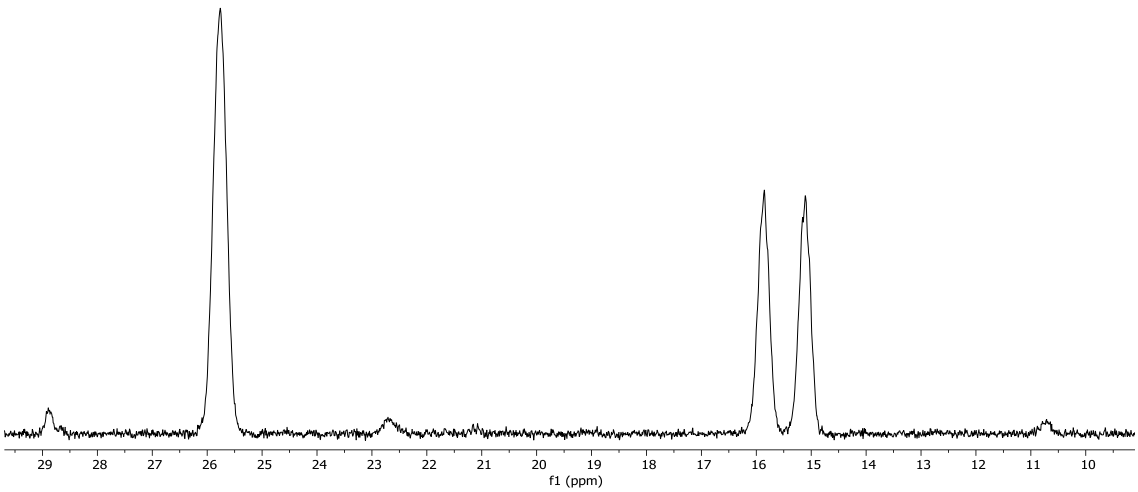

Figure S33 ^{31}P NMR of complex 3a in CD_3OD

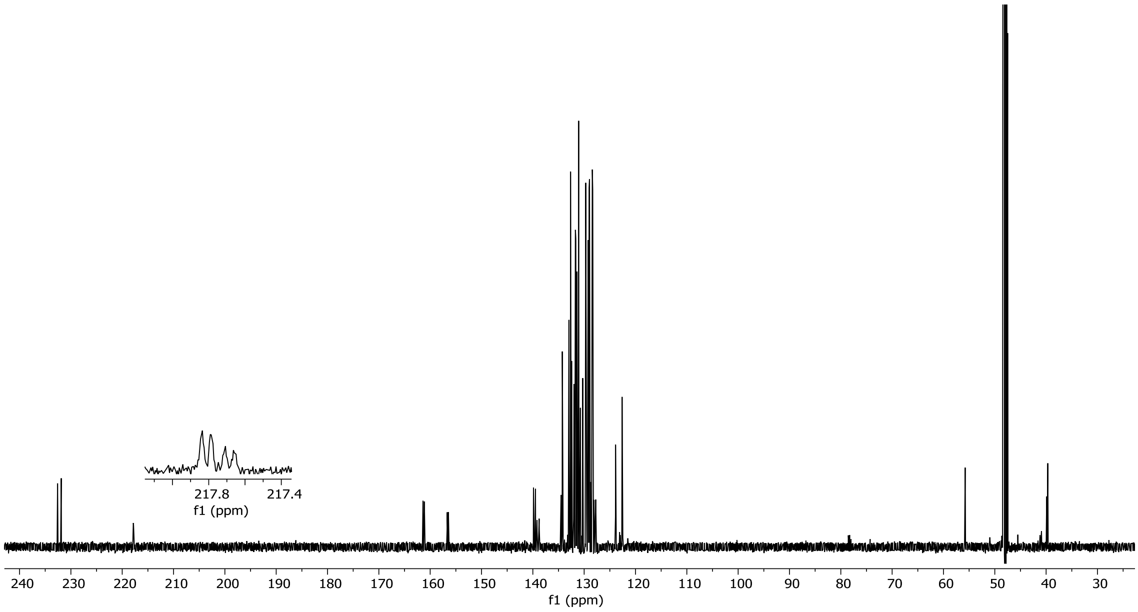

Figure S34 $^{13}C\{^1H\}$ NMR of complex 3a in $CDCl_3$

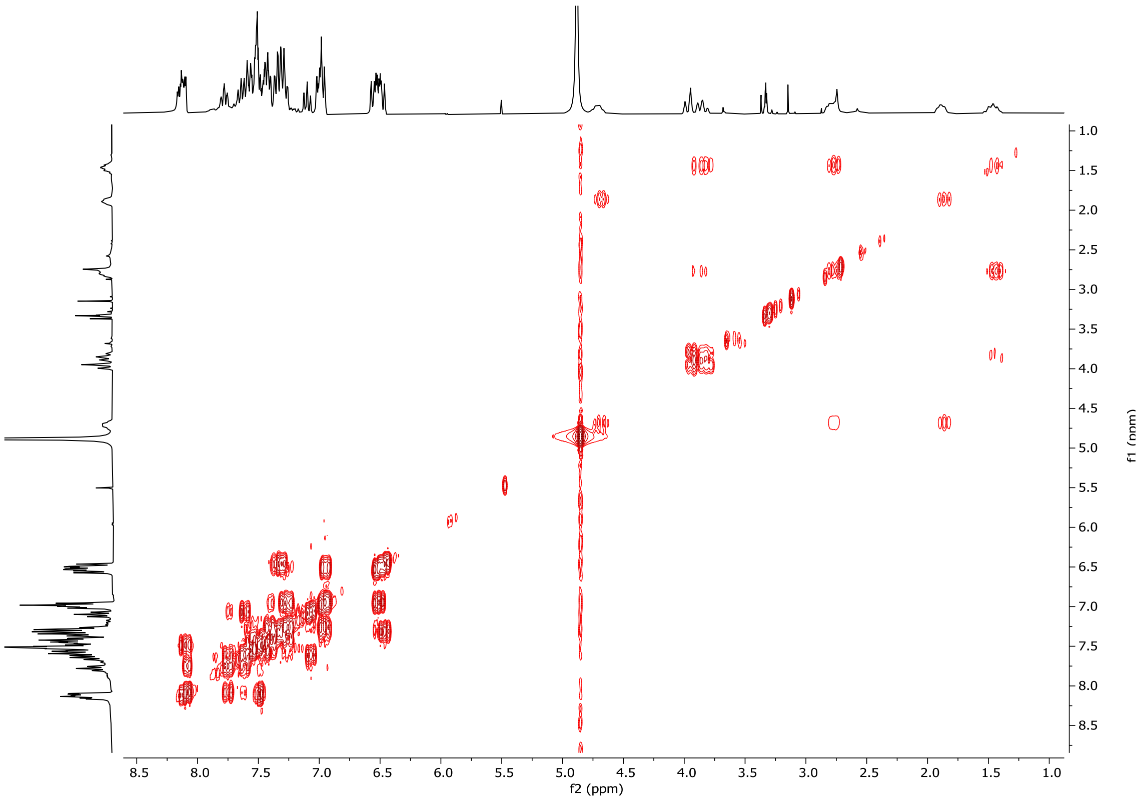

Figure S35 COSY spectrum of complex 3a in CD_3OD

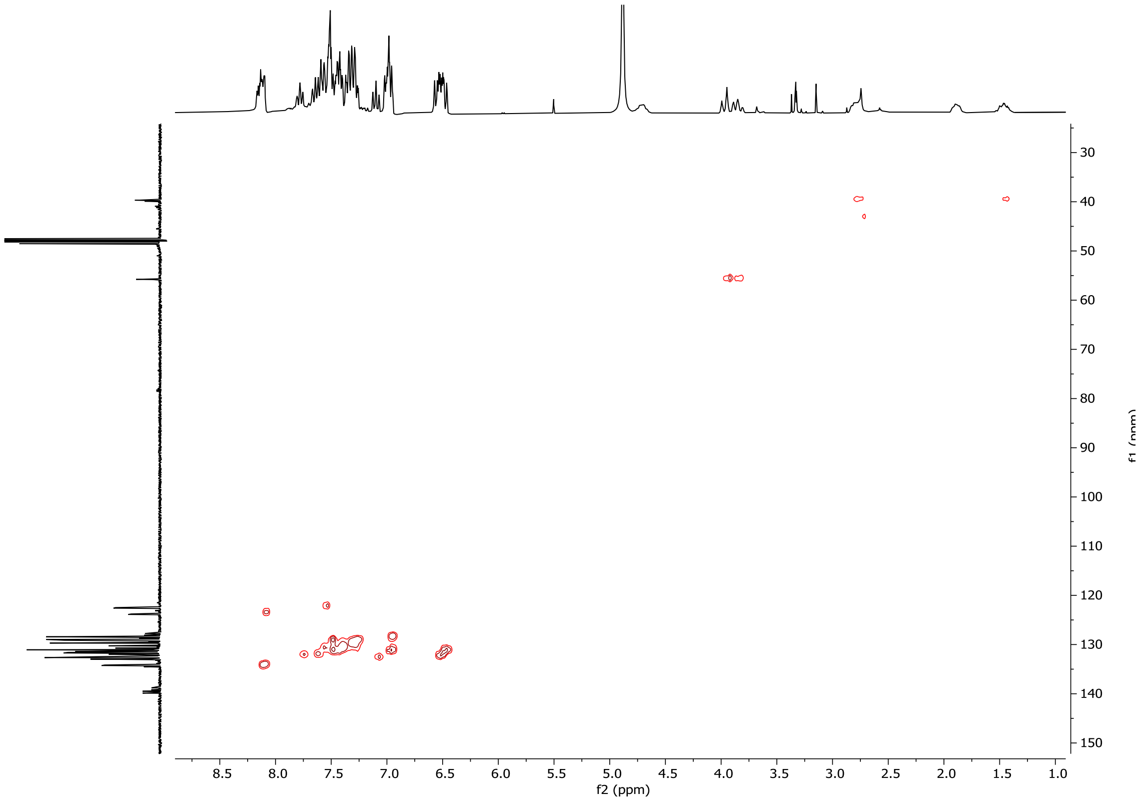

Figure S36 ^1H-^{13}C HSQC spectrum of complex 3a in CD_3OD

Compound 3b

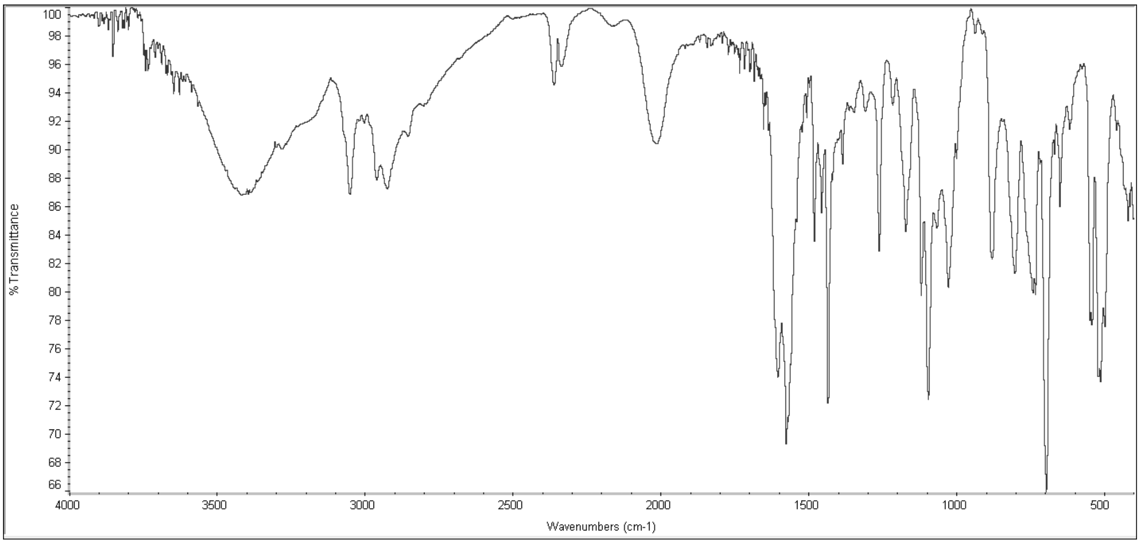

Figure S37 IR Spectrum of complex 3b

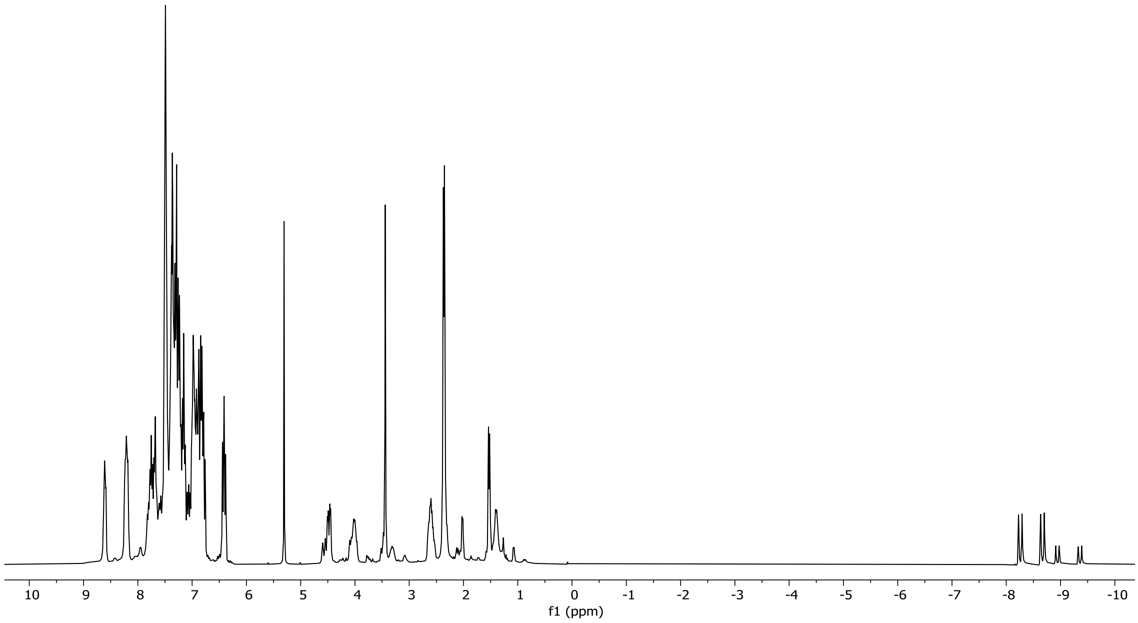

Figure S38 ^{1}H NMR of complex 3b in $CDCl_3$

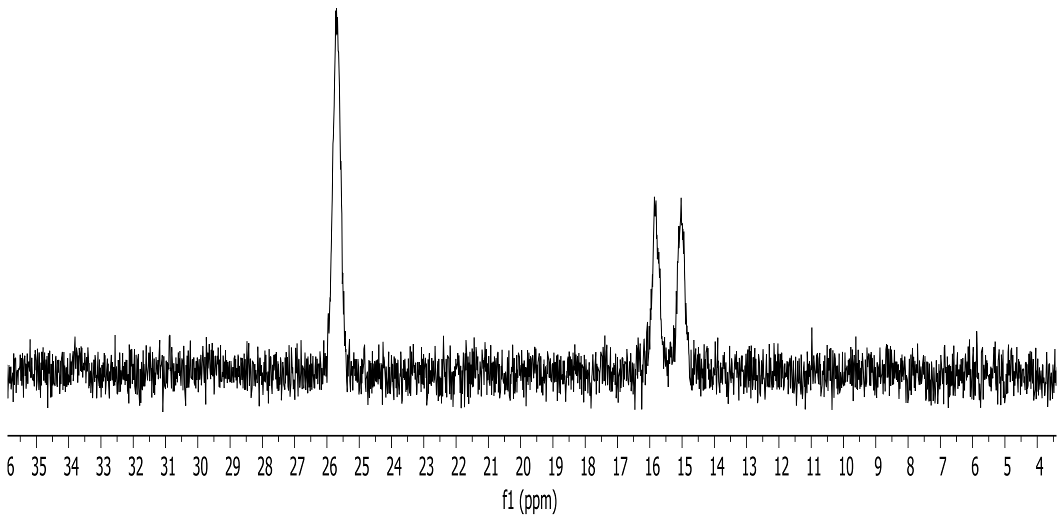

Figure S39 ^{31}P NMR of complex 3b in $CDCl_3$

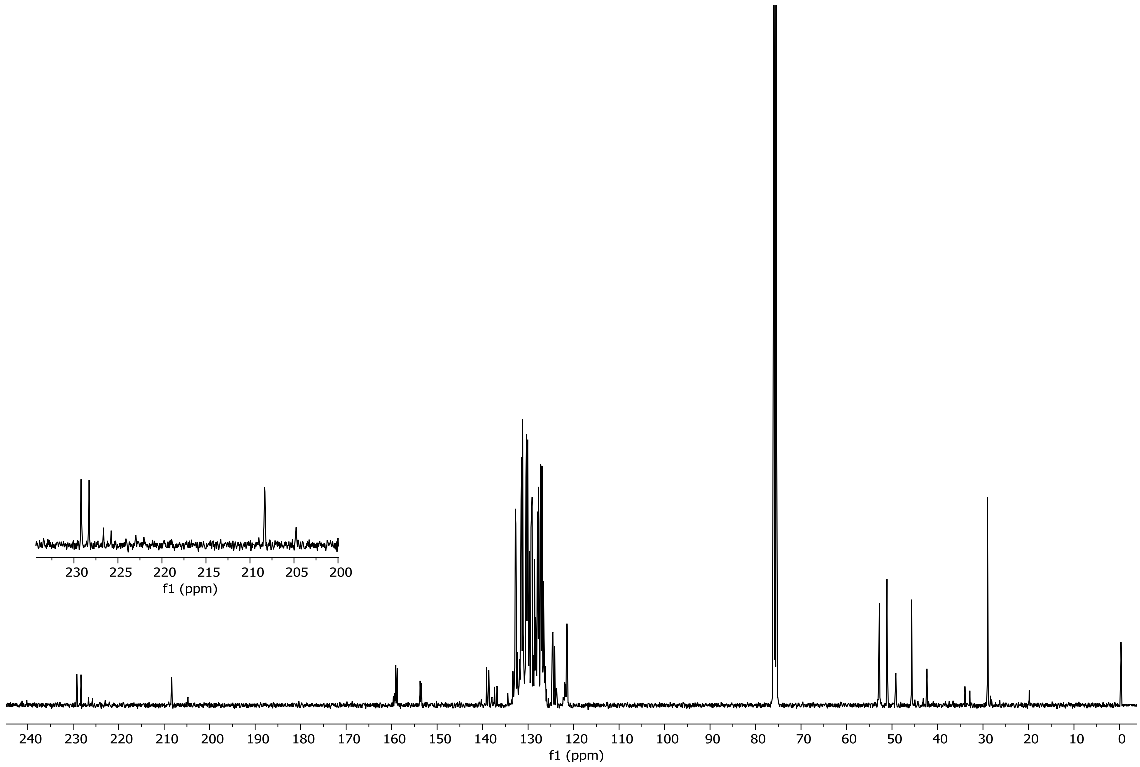

Figure S40 $^{13}C\{^1H\}$ NMR of complex 3b in $CDCl_3$

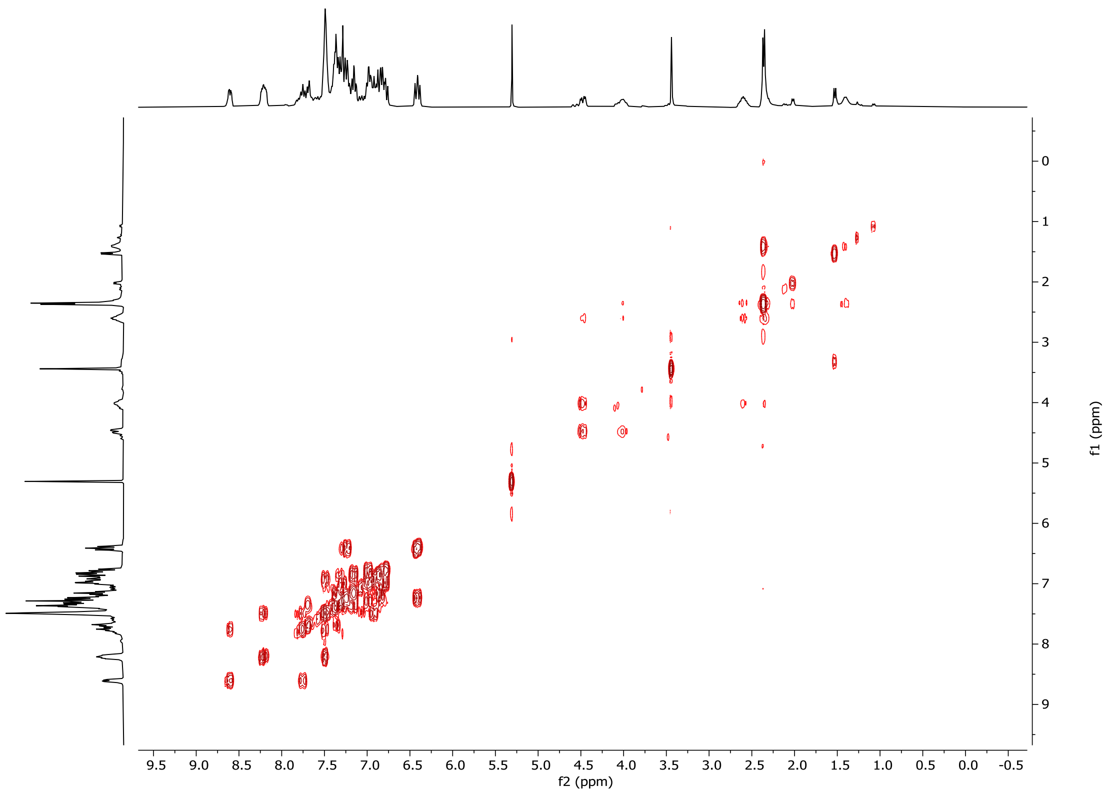

Figure S41 COSY spectrum of complex 3b in $CDCl_3$

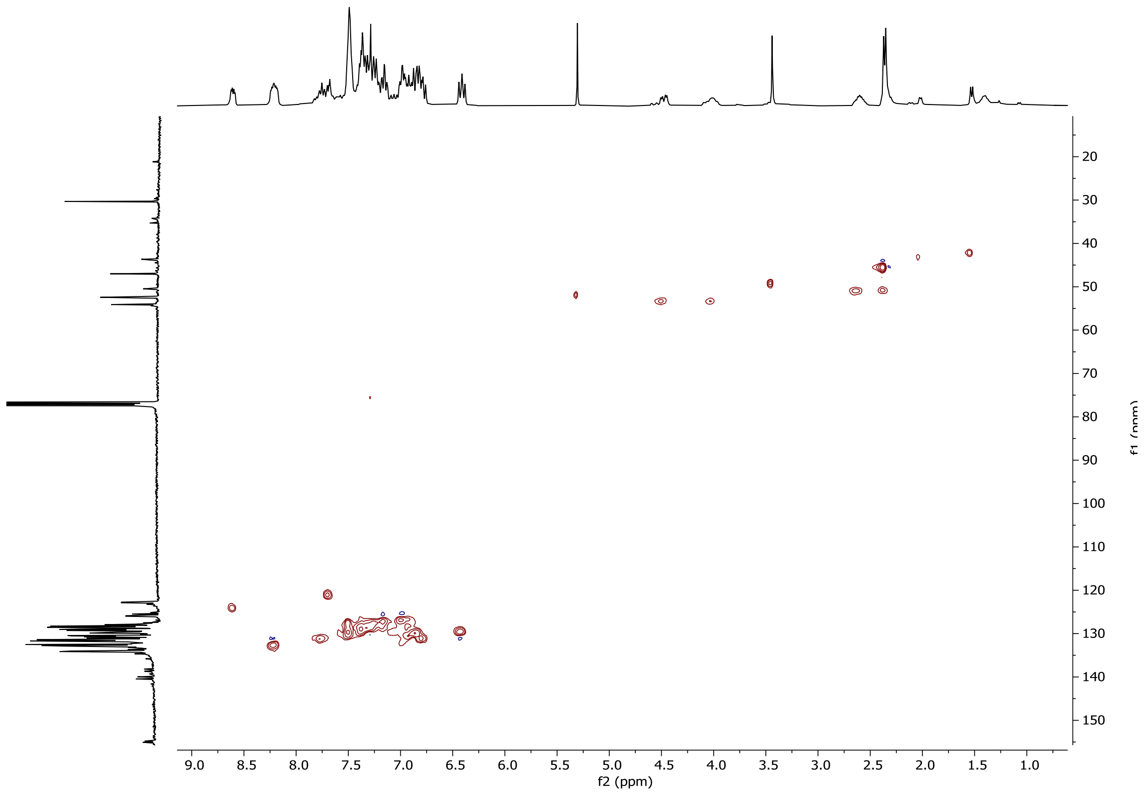

Figure S42 ^{1}H-^{13}C HSQC spectrum of complex 3b in $CDCl_3$

Compound 3d

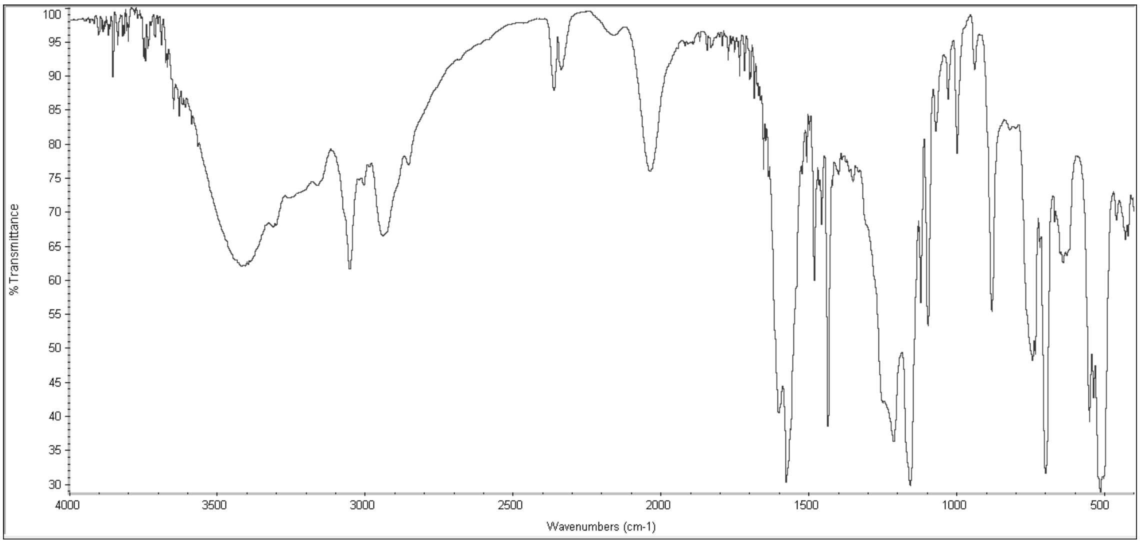

Figure S43 IR Spectrum of complex 3d

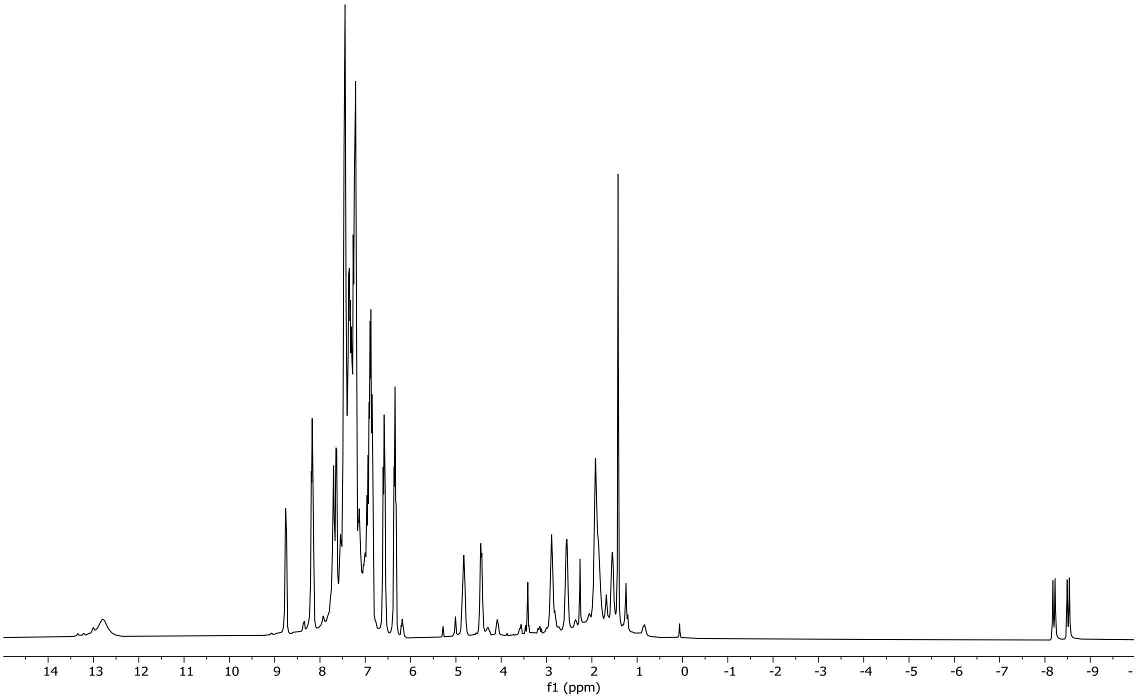

Figure S44 ^{1}H NMR of complex 3d in $CDCl_3$

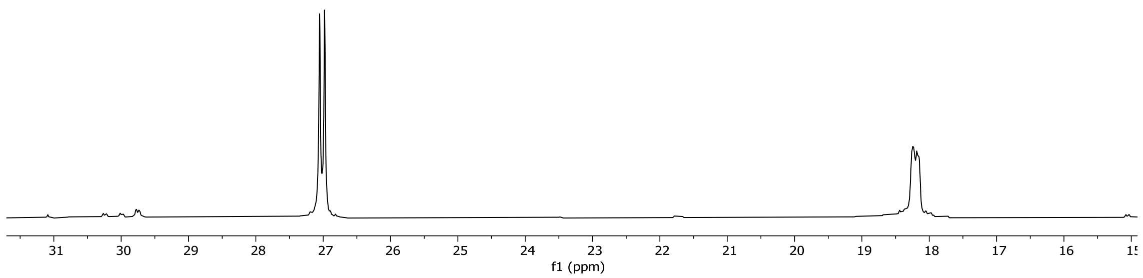

Figure S45 $^{31}P\{^1H\}$ NMR of complex 3d in $CDCl_3$

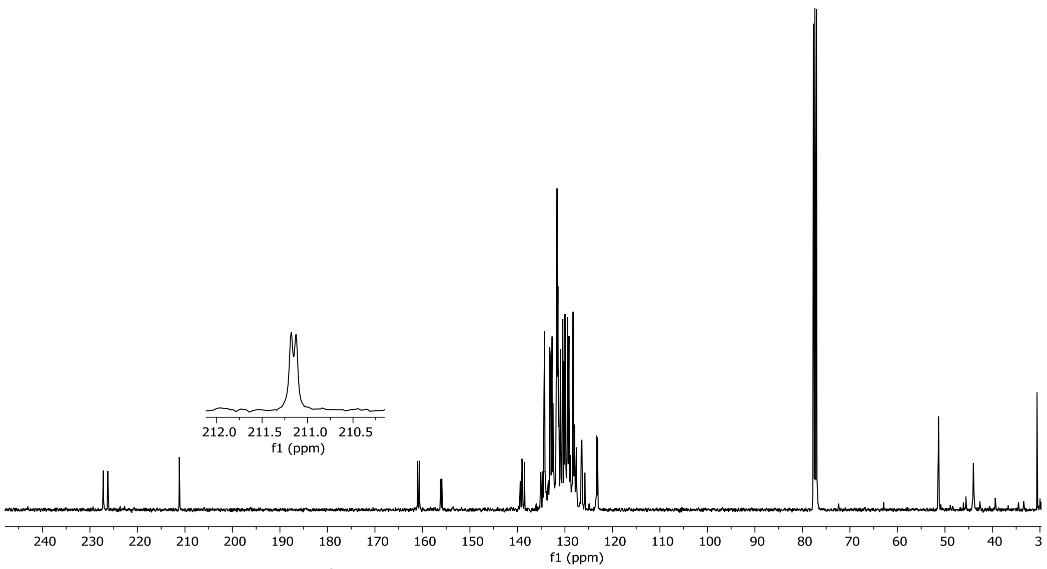

Figure S46 $^{13}C\{^1H\}$ NMR of complex 3d in $CDCl_3$

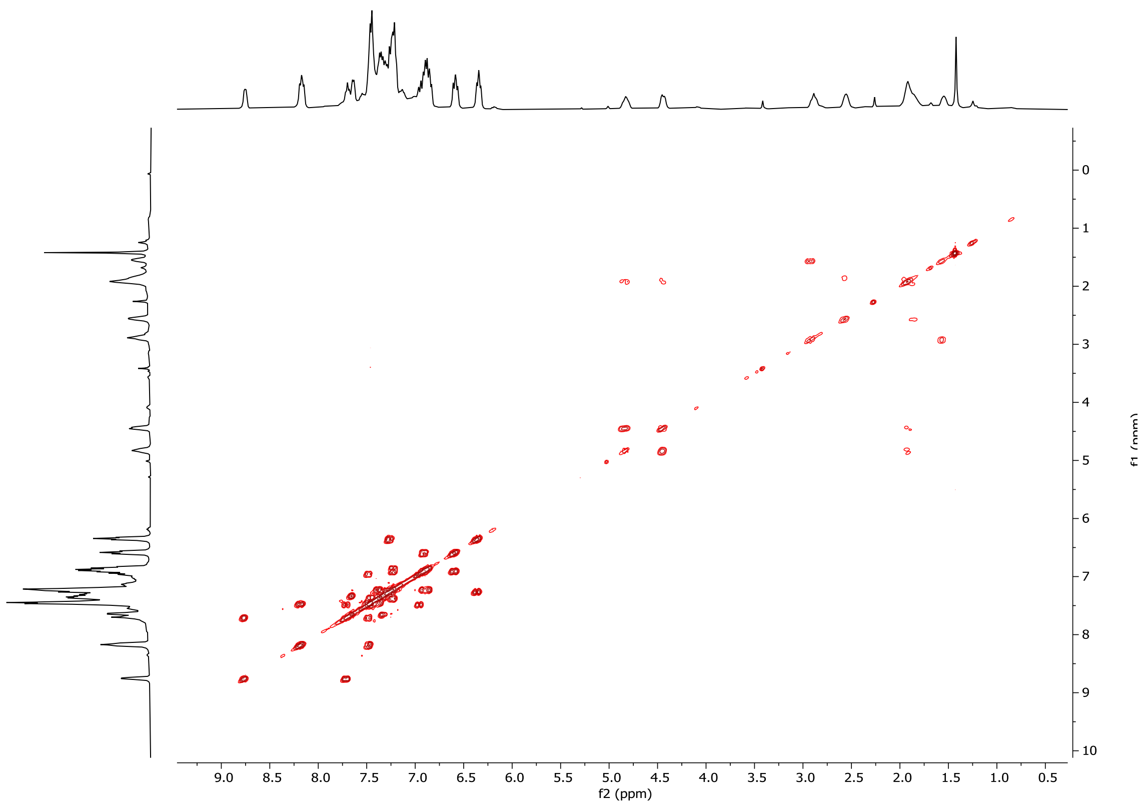

Figure S47 COSY spectrum of complex 3d in $CDCl_3$

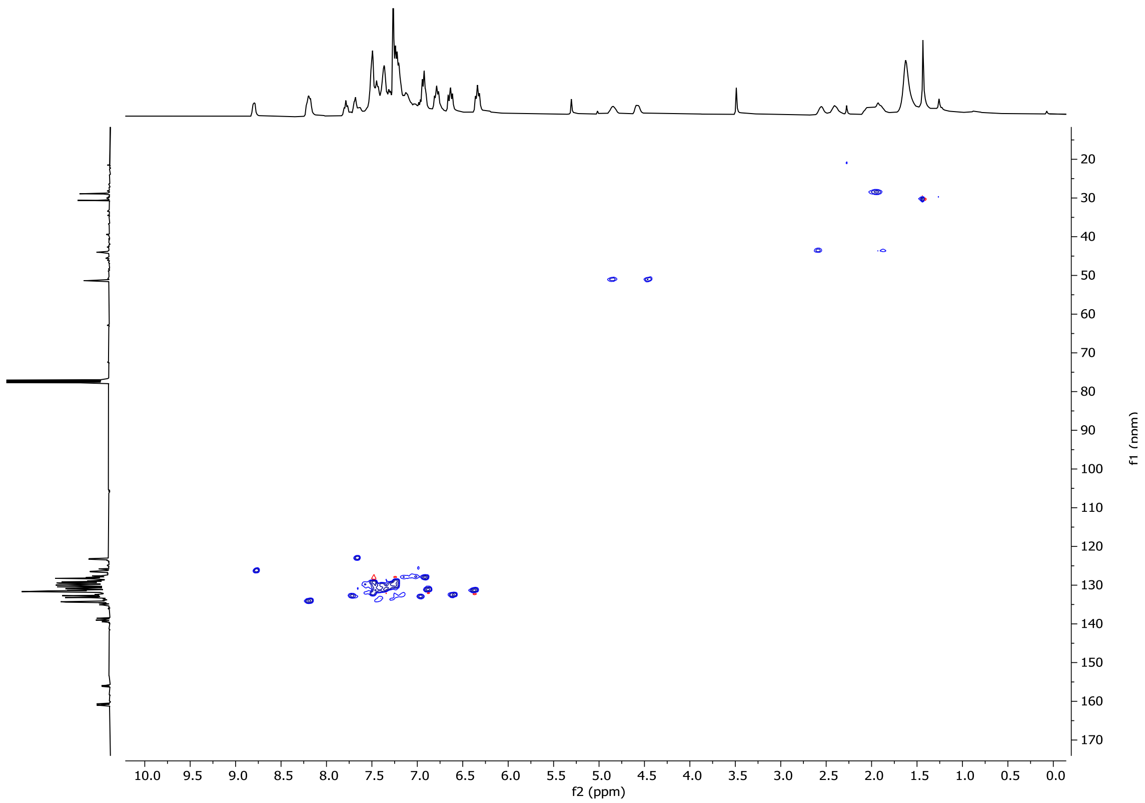

Figure S48 ^{1}H-^{13}C HSQC spectrum of complex 3d in $CDCl_3$

Compound 3e

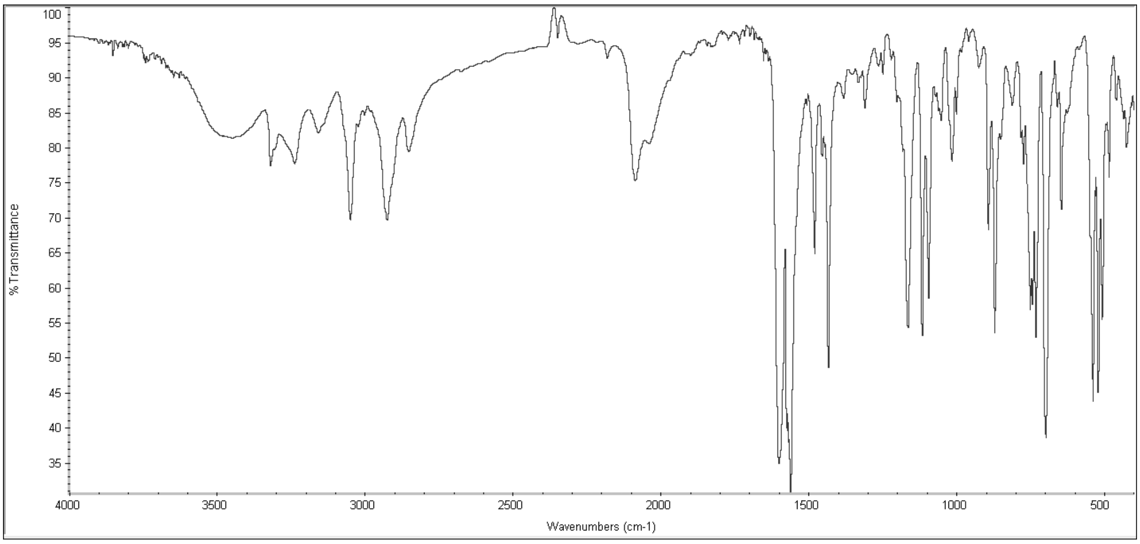

Figure S49 IR Spectrum of complex 3e

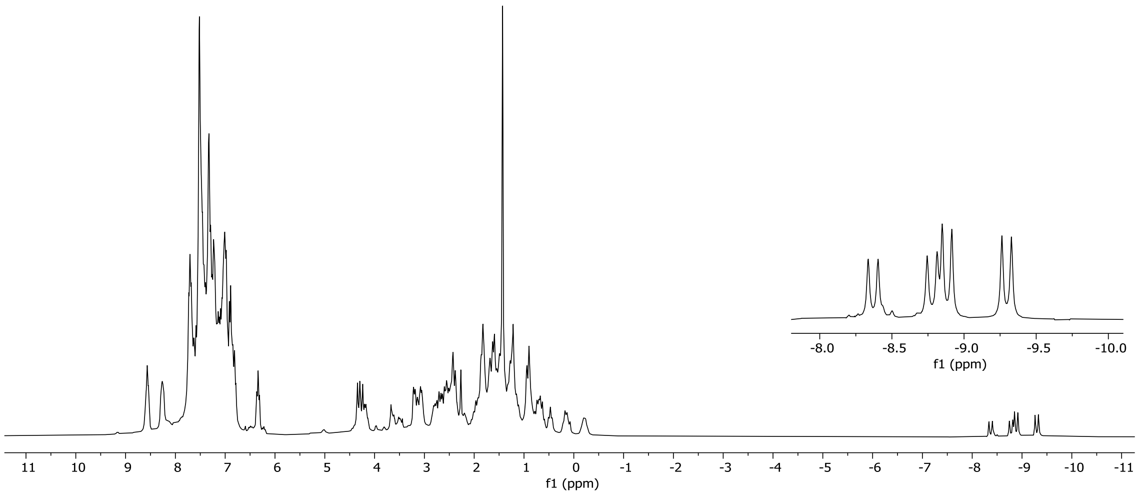

Figure S50 ^{1}H NMR of complex 3e in $CDCl_3$

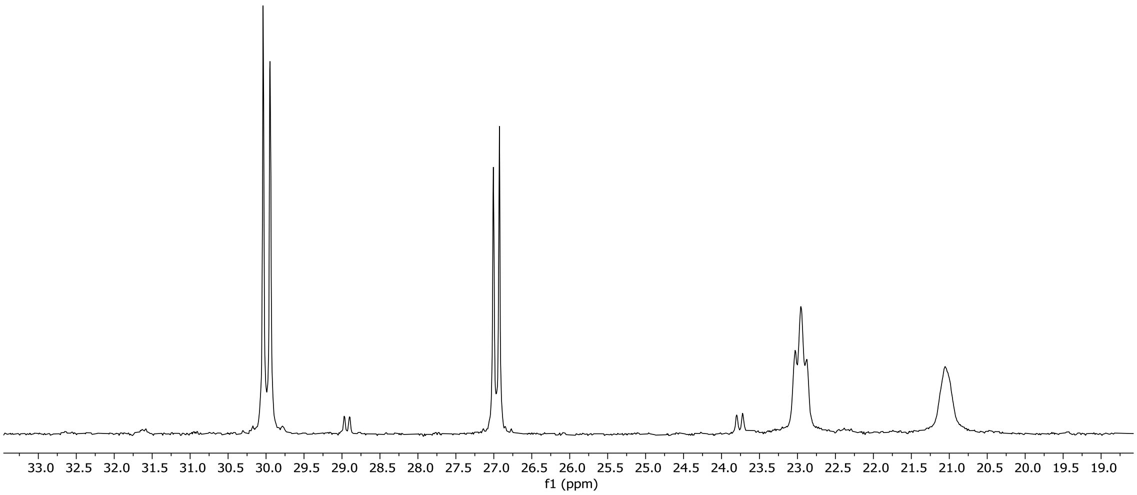

Figure S51 $^{31}P\{^{1}H\}$ NMR of complex 3e in $CDCl_3$

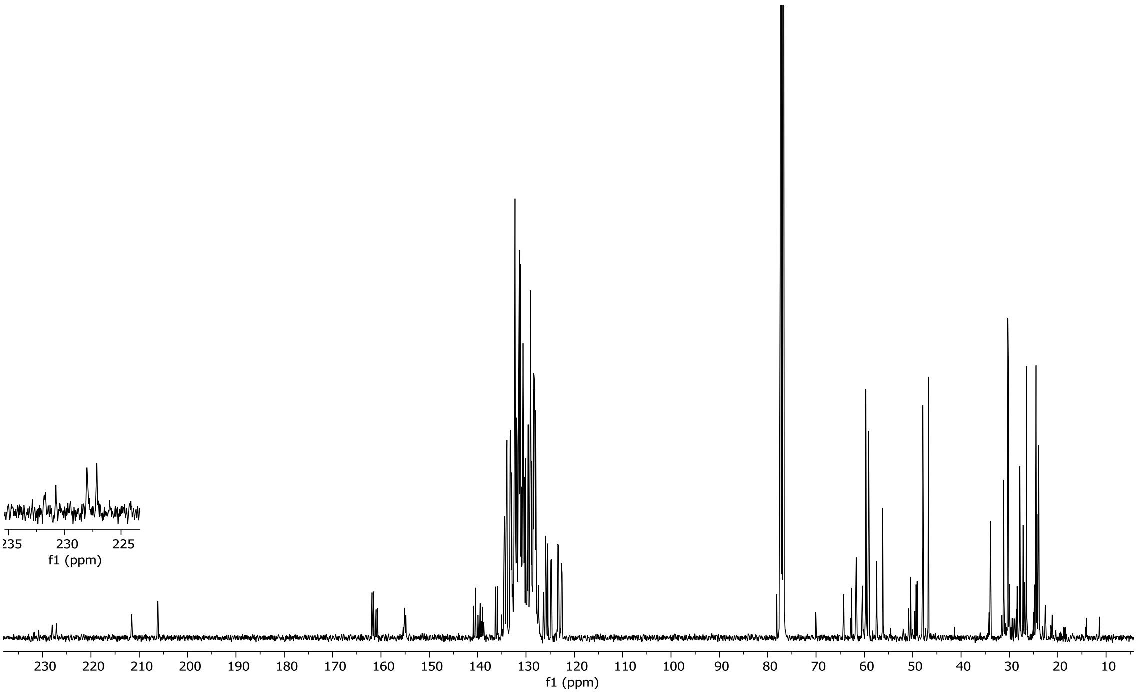

Figure S52 $^{13}C\{^{1}H\}$ NMR of complex 3e in $CDCl_3$

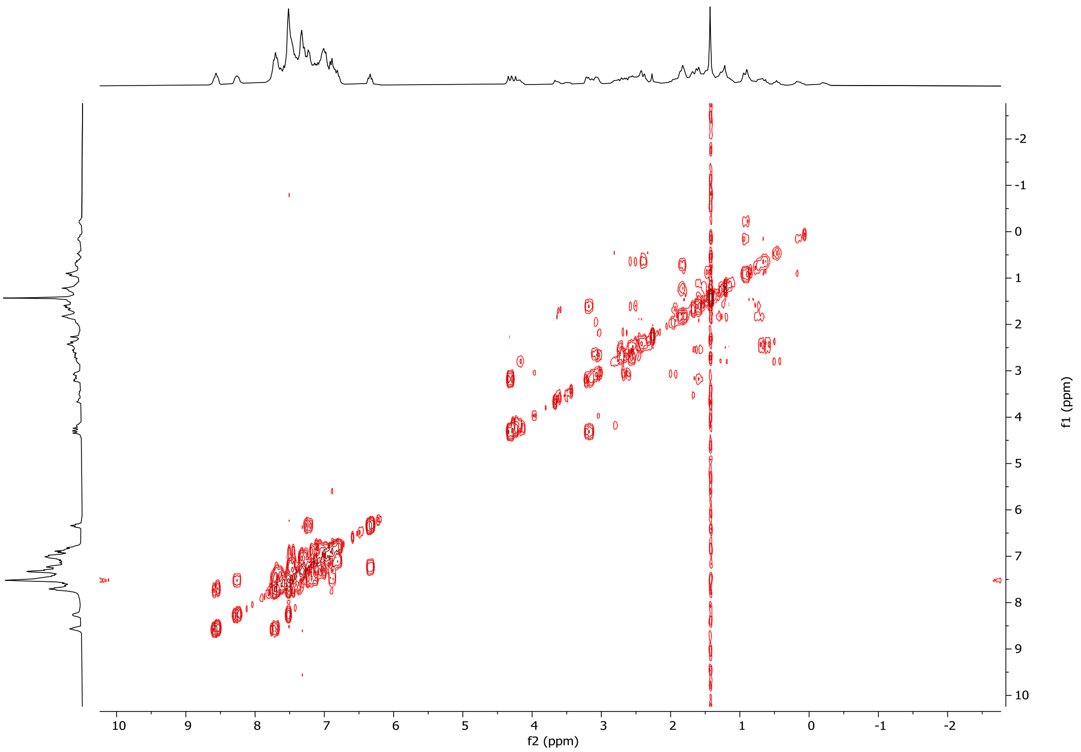

Figure S53 COSY spectrum of complex 3e in $CDCl_3$

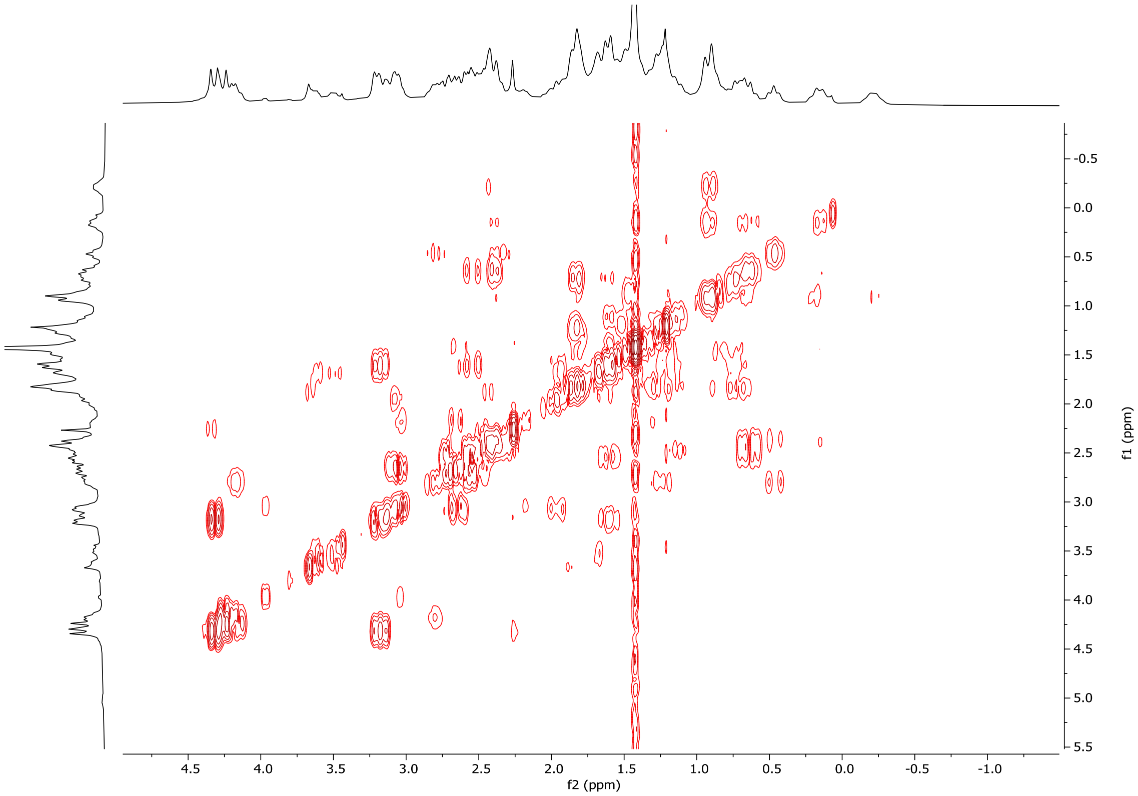

Figure S54 COSY spectrum of complex 3e in $CDCl_3$

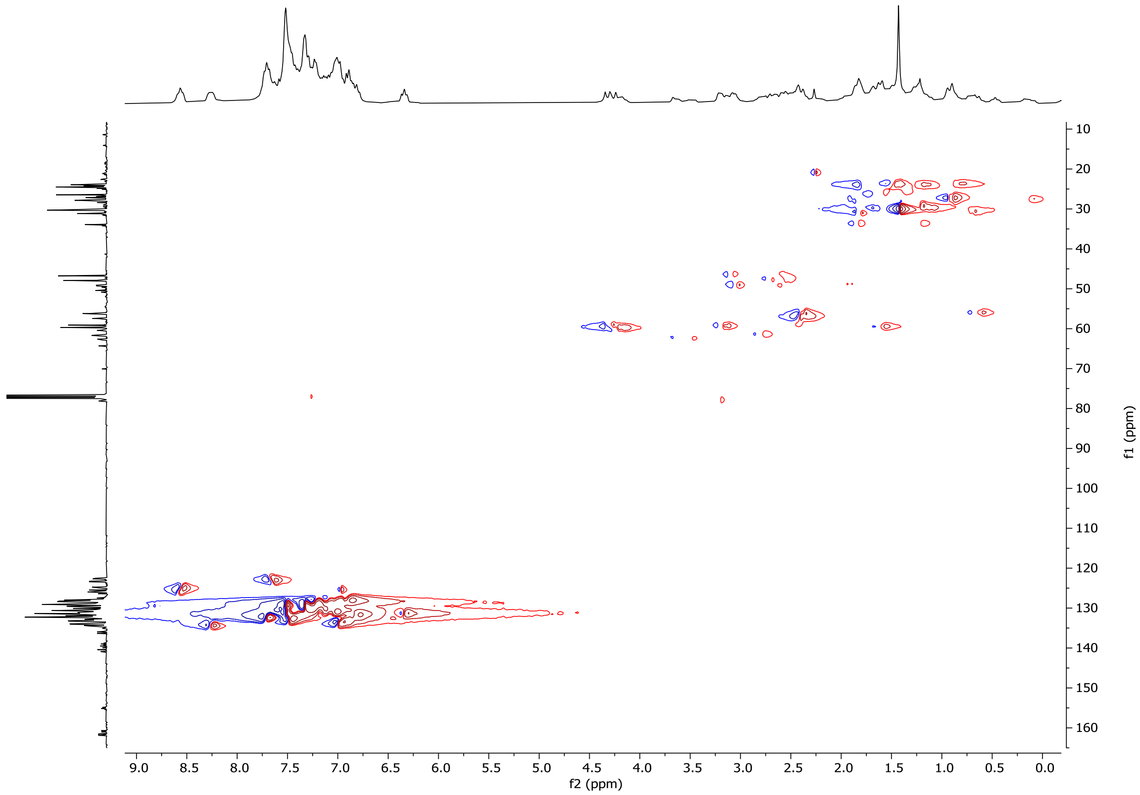

Figure S55 ^1H-^{13}C HSQC spectrum of complex 3e in $CDCl_3$

Compound 4a

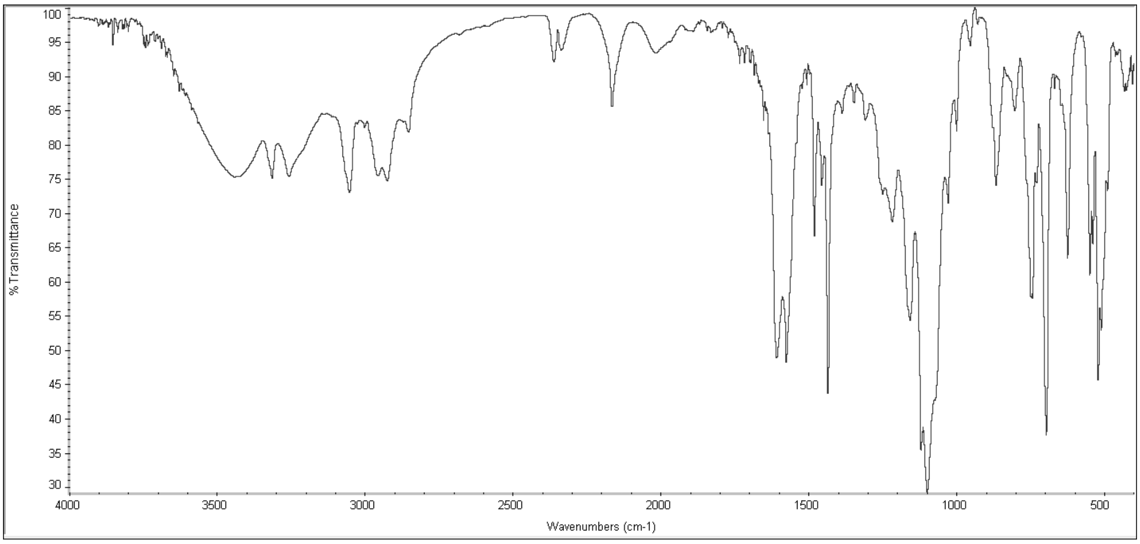

Figure S56 IR Spectrum of the [4a]ClO_4/[3a]ClO_4 mixture

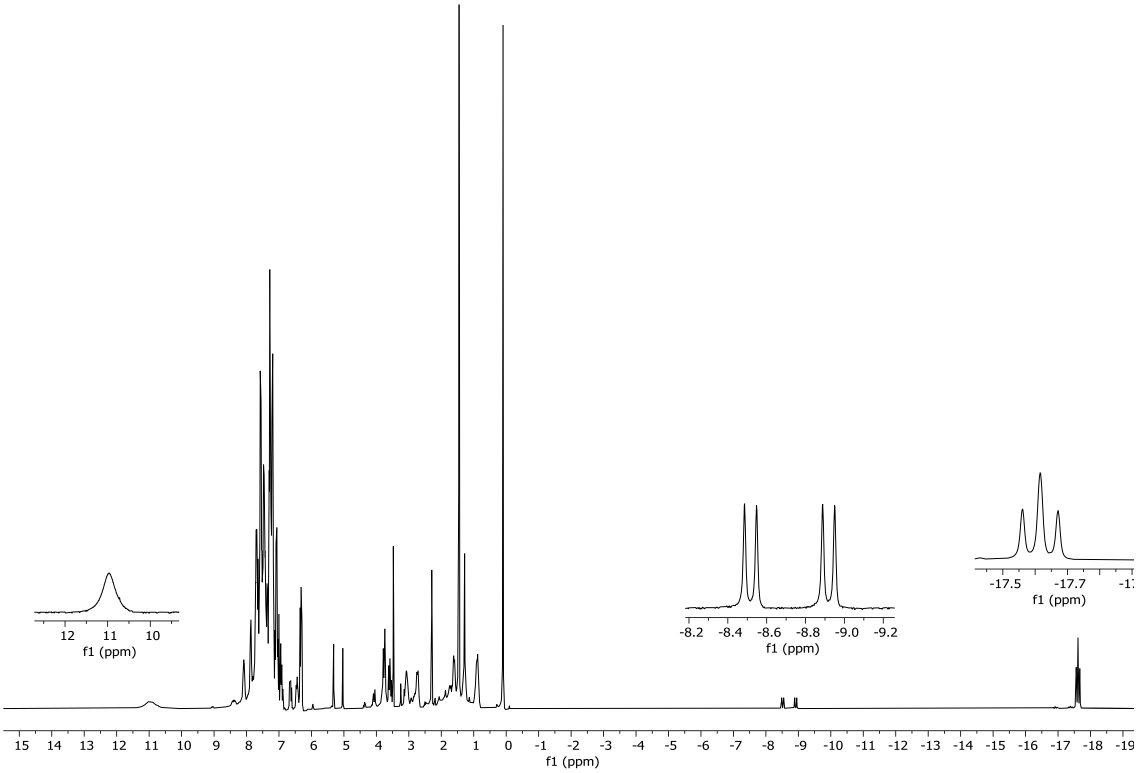

Figure S57 ^{1}H NMR of the [4a]ClO_4/[3a]ClO_4 mixture in $CDCl_3$

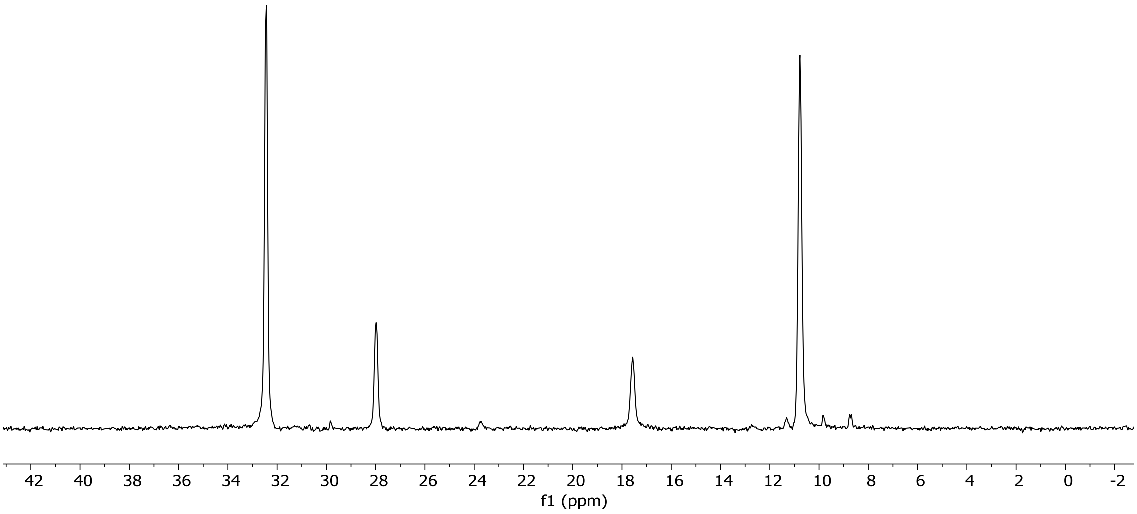

Figure S58 $^{31}P\{^{1}H\}$ NMR of the [4a]ClO_4/[3a]ClO_4 mixture in $CDCl_3$

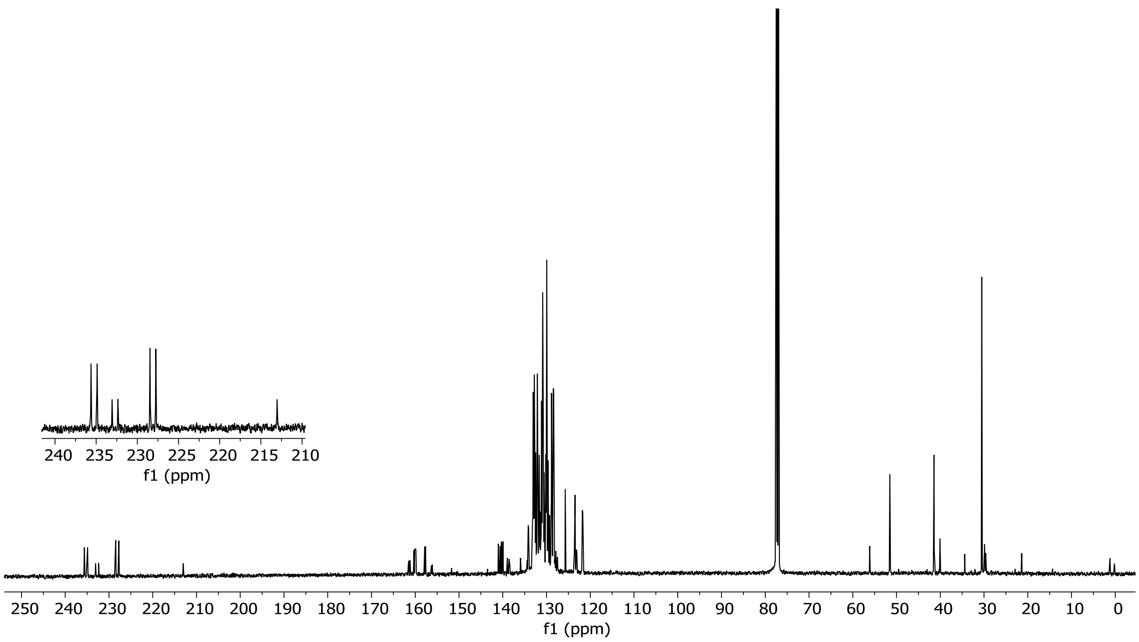

Figure S59 $^{13}C\{^1H\}$ NMR of the [4a]ClO$_4$/[3a]ClO$_4$ mixture in CDCl$_3$

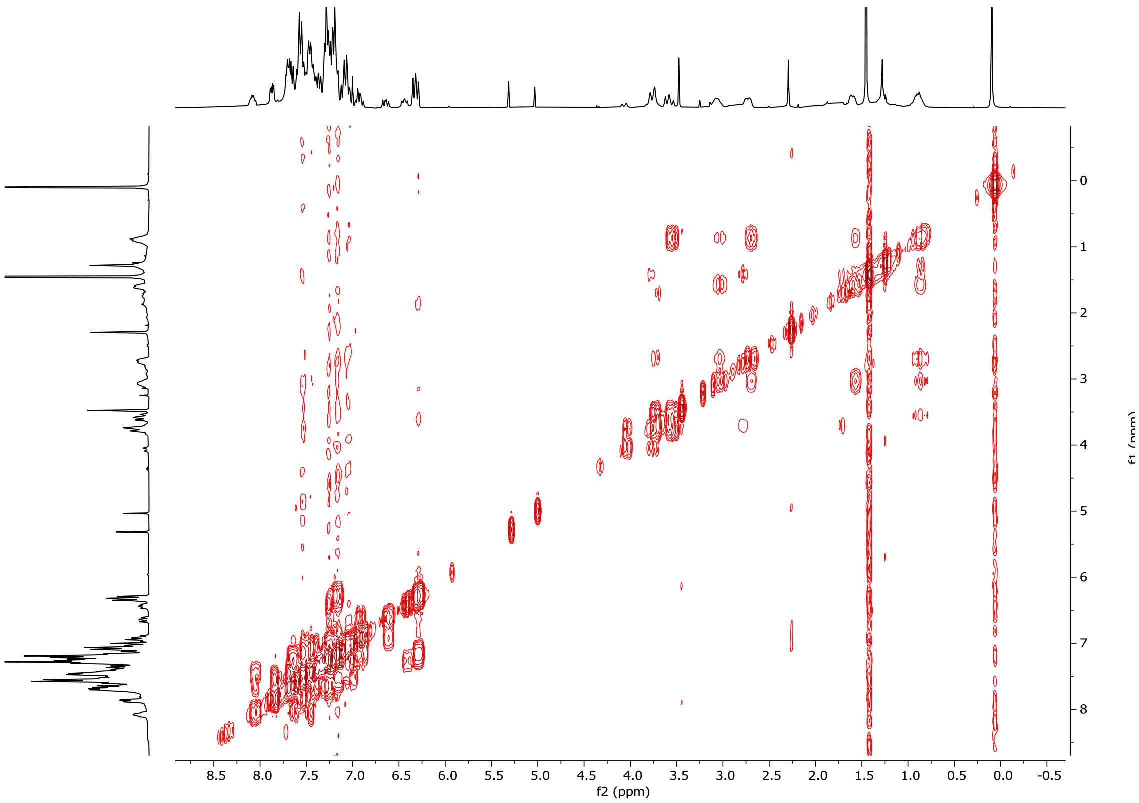

Figure S60 COSY spectrum of the [4a]ClO$_4$/[3a]ClO$_4$ mixture in CDCl$_3$

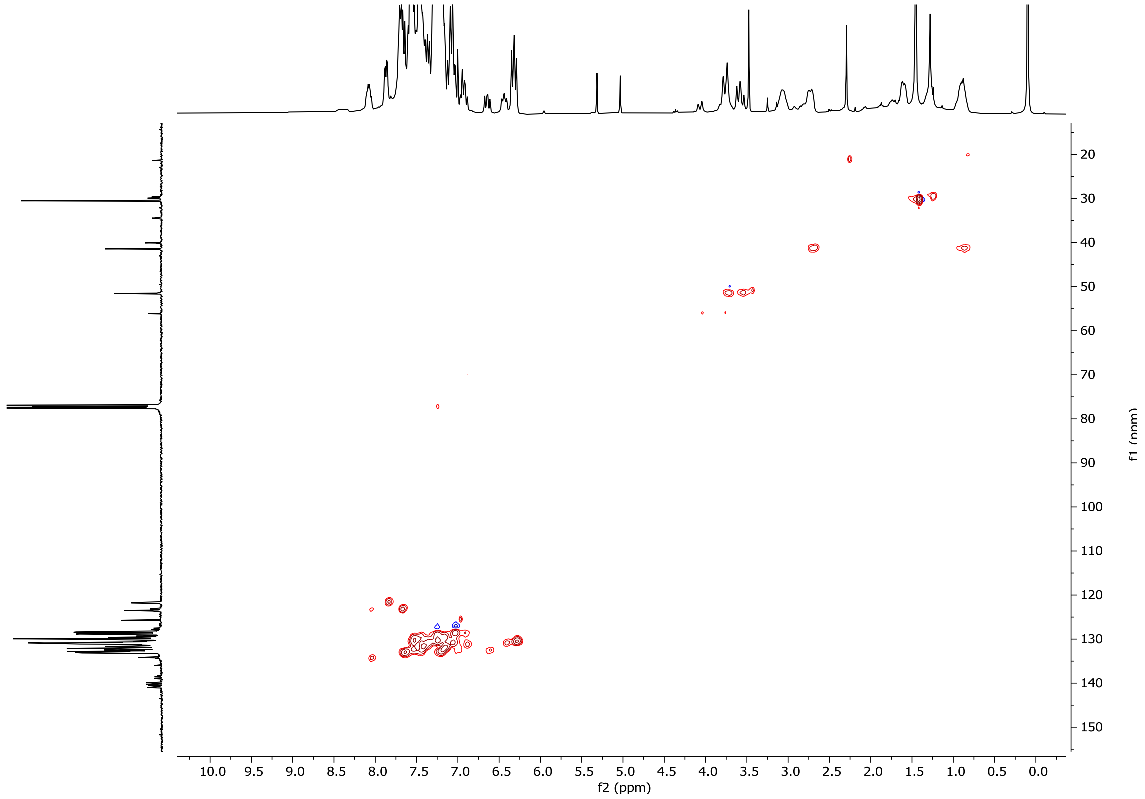

Figure S61 ^{1}H-^{13}C HSQC spectrum of the [4a]ClO_4/[3a]ClO_4 mixture in $CDCl_3$

Compound 4b

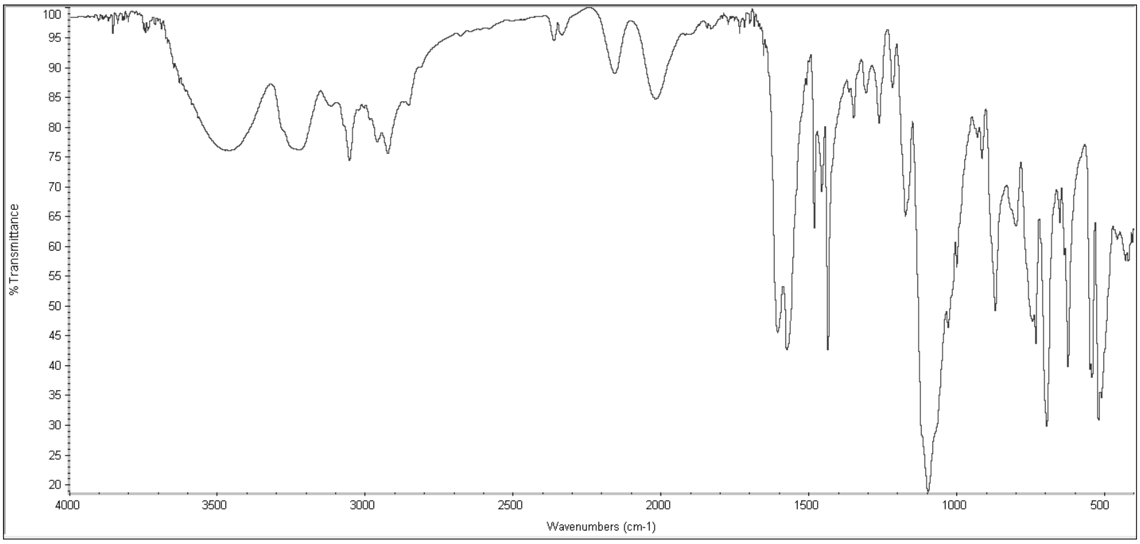

Figure S62 IR Spectrum of the [4b]ClO_4/[3b]ClO_4 mixture

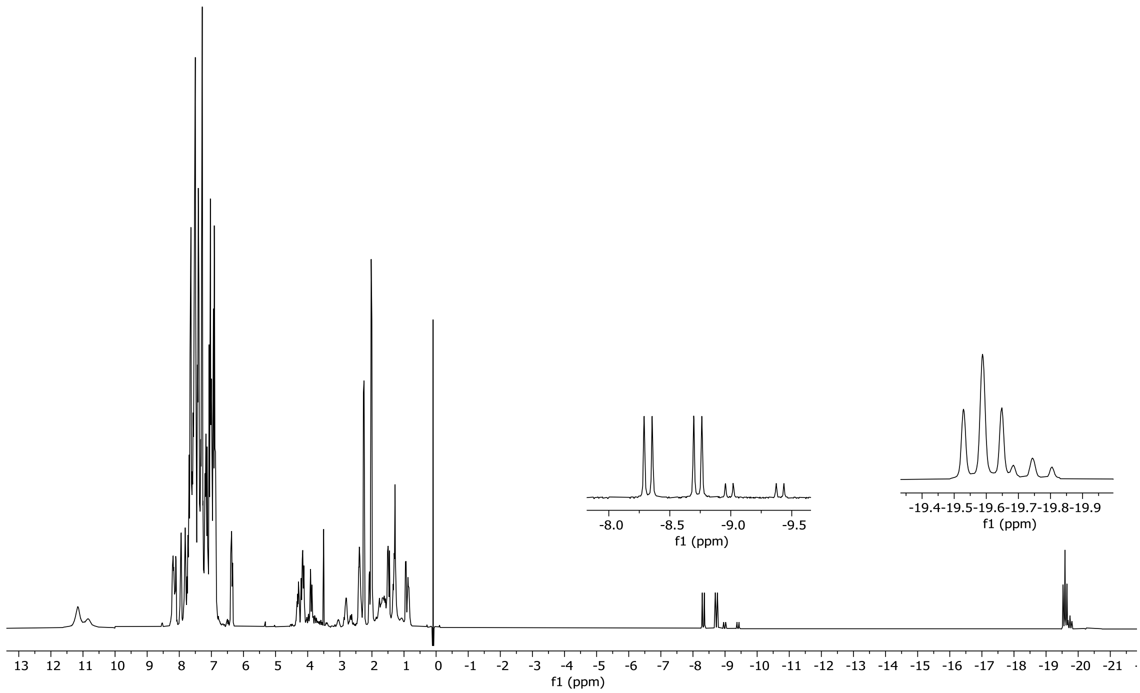

Figure S63 ^{1}H NMR of the [4b]ClO_4/[3b]ClO_4 mixture in $CDCl_3$

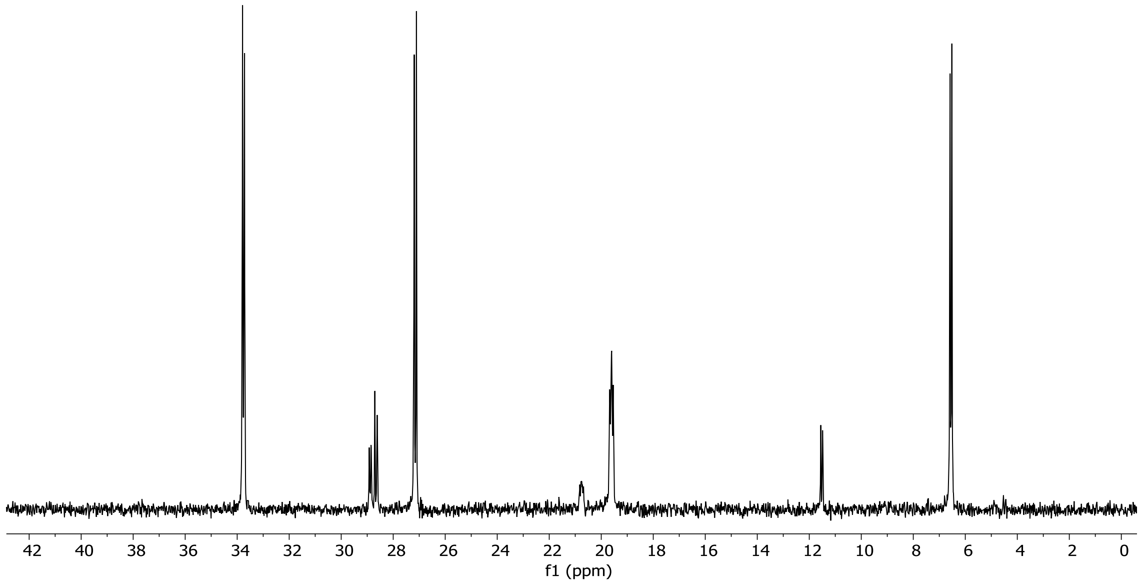

Figure S64 $^{31}P\{^{1}H\}$ NMR of the [4b]ClO_4/[3b]ClO_4 mixture in $CDCl_3$

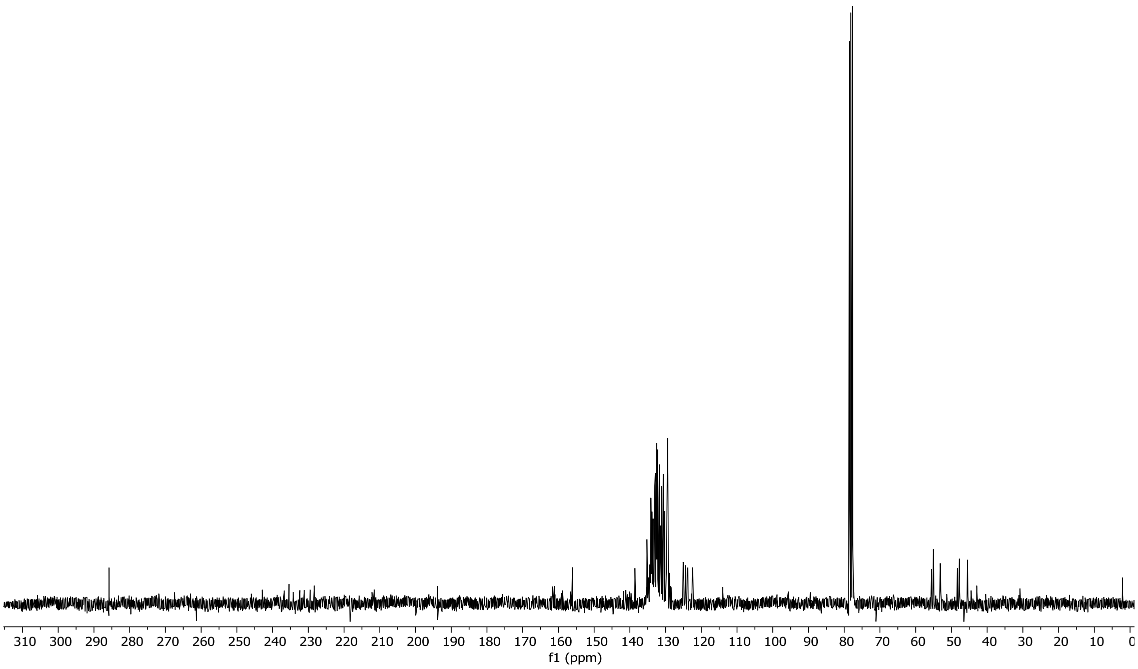

Figure S65 $^{13}C\{^1H\}$ NMR of the [4b]ClO_4/[3b]ClO_4 mixture in $CDCl_3$

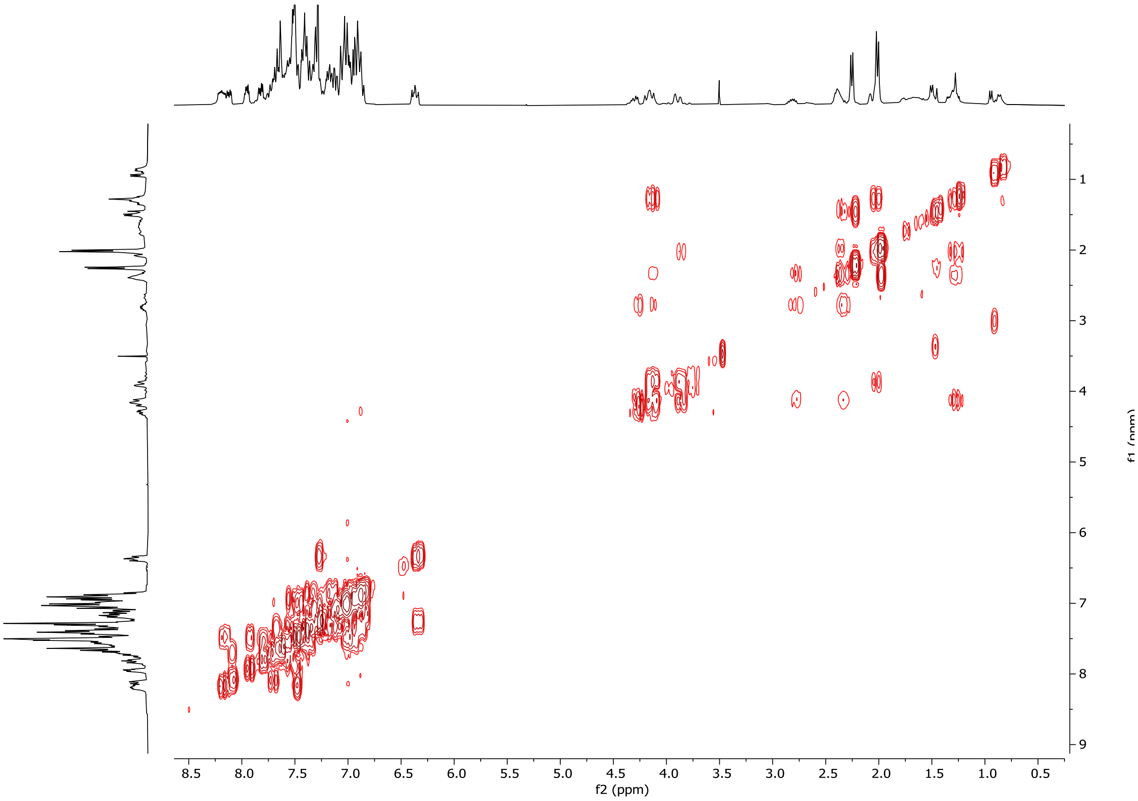

Figure S66 COSY spectrum of the [4b]ClO_4/[3b]ClO_4 mixture in $CDCl_3$

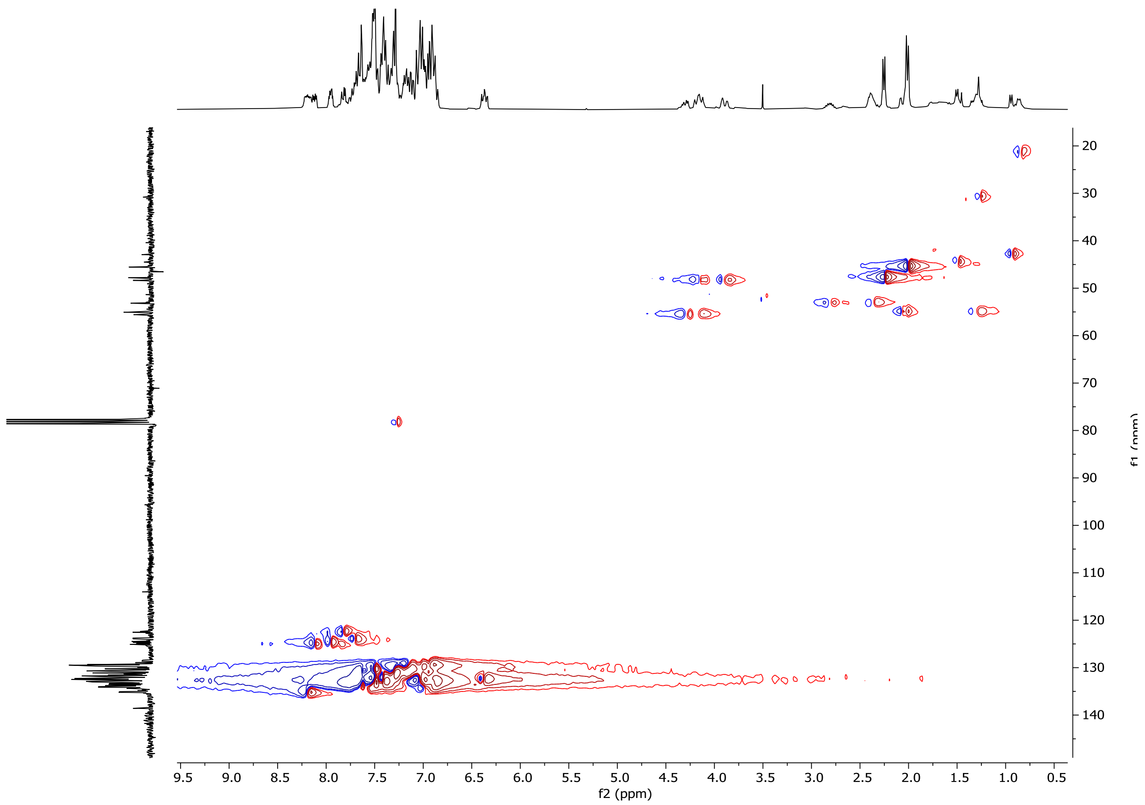

Figure S67 ^{1}H-^{13}C HSQC spectrum of the [4b]ClO_4/[3b]ClO_4 mixture in $CDCl_3$

Compound 5a

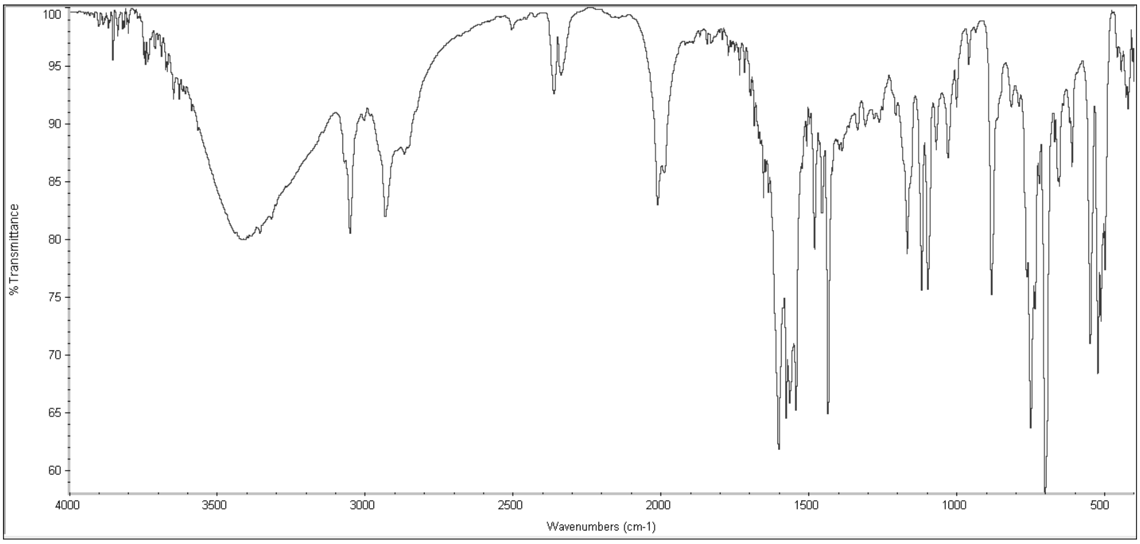

Figure S68 IR Spectrum of complex 5a

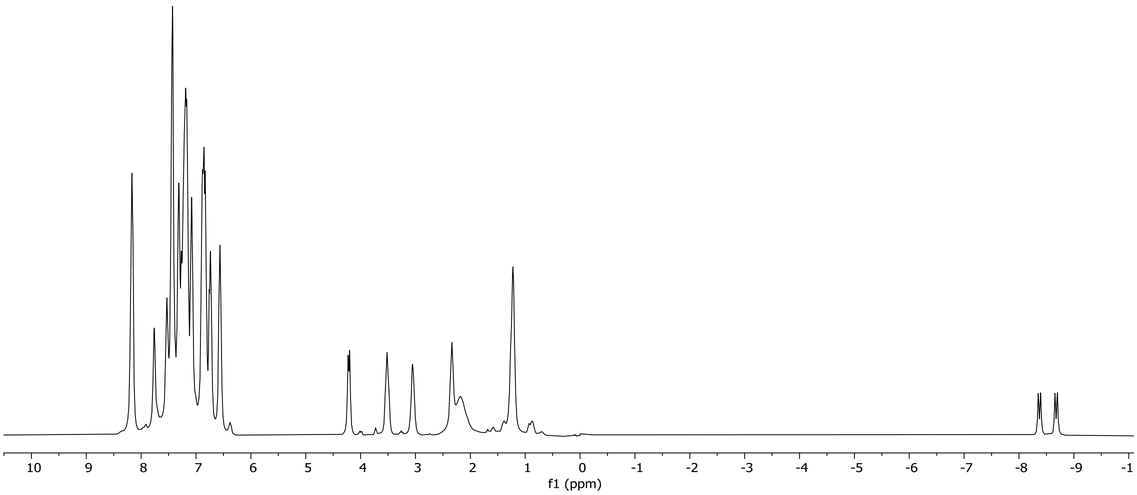

Figure S69 ^{1}H NMR of complex 5a in $CDCl_3$

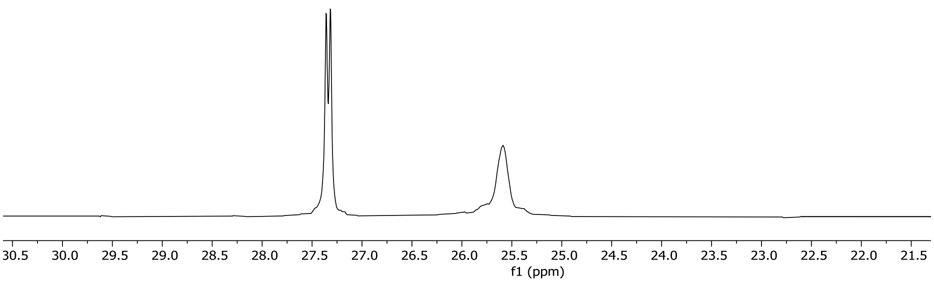

Figure S70 $^{31}P\{^{1}H\}$ NMR of complex 5a in $CDCl_3$

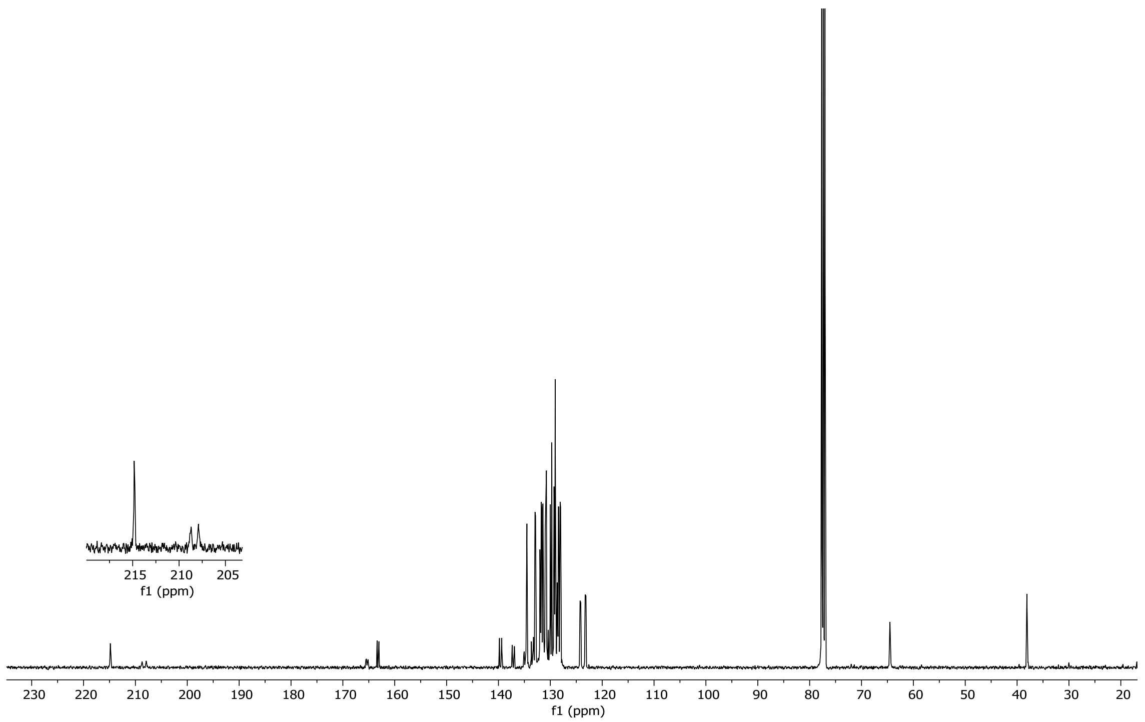

Figure S71 $^{13}C\{^{1}H\}$ NMR of complex 5a in $CDCl_3$

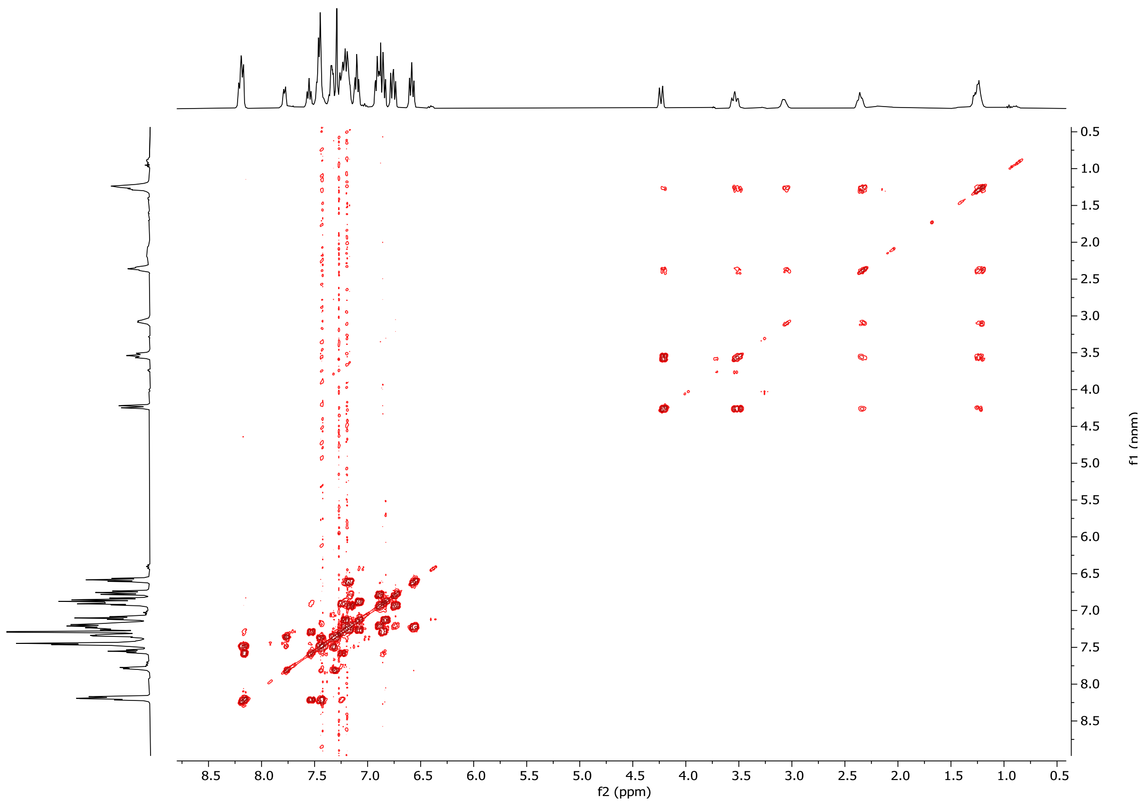

Figure S72 COSY spectrum of complex 5a in $CDCl_3$

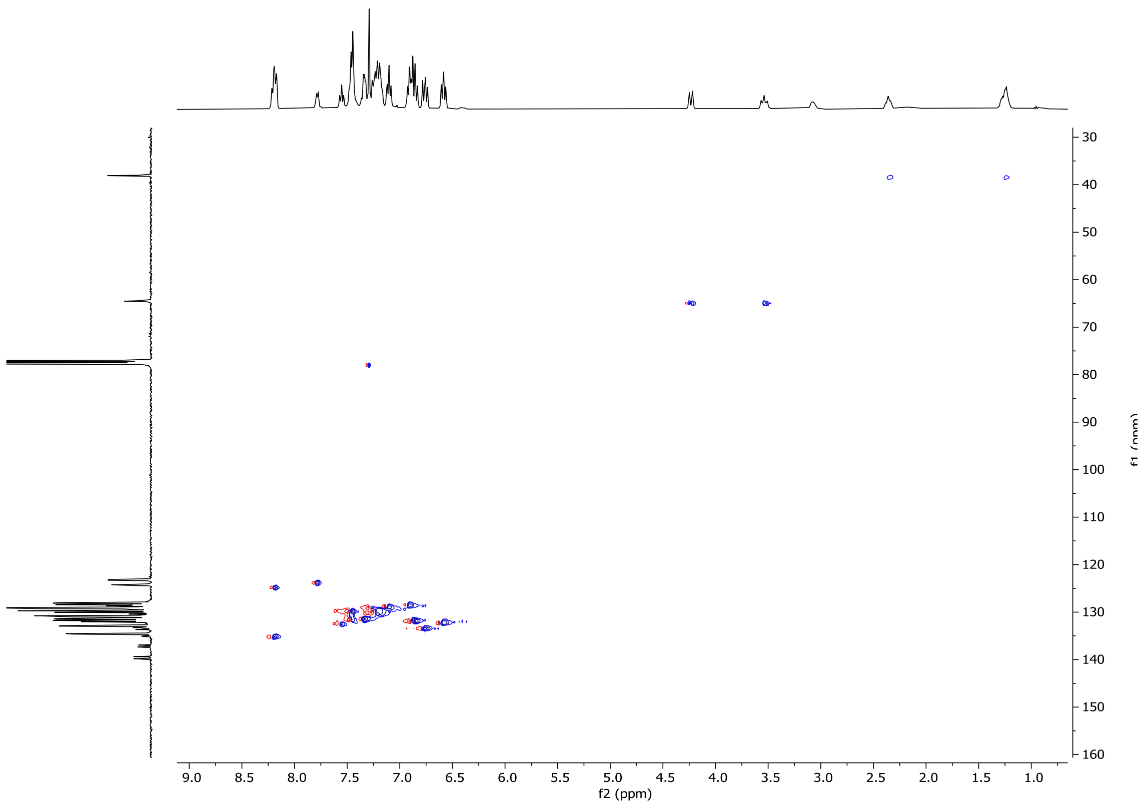

Figure S73 ^{1}H-^{13}C HSQC spectrum of complex 5a in $CDCl_3$

Compound 5b

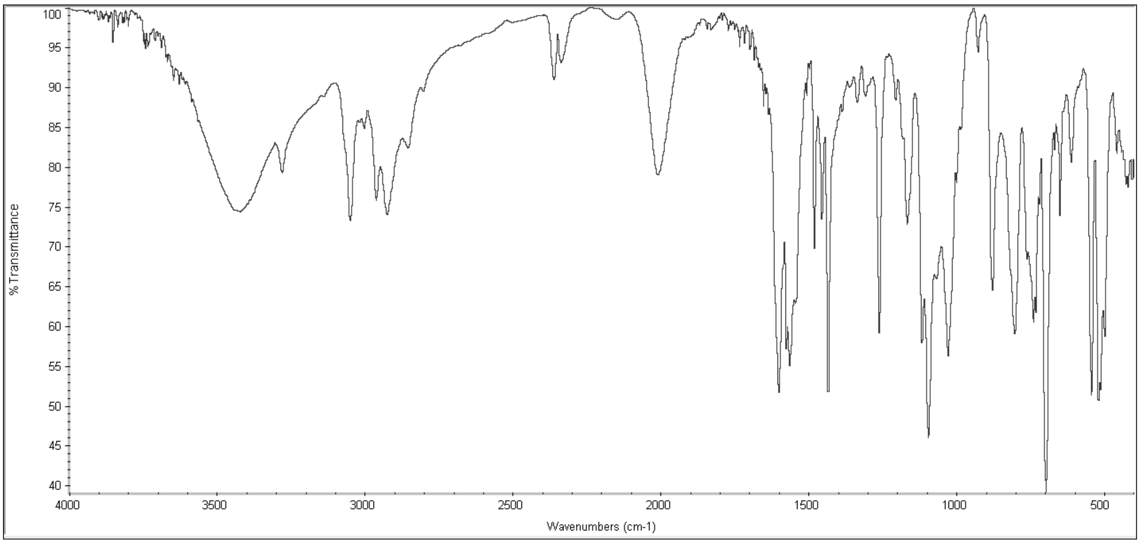

Figure S74 IR Spectrum of complex 5b

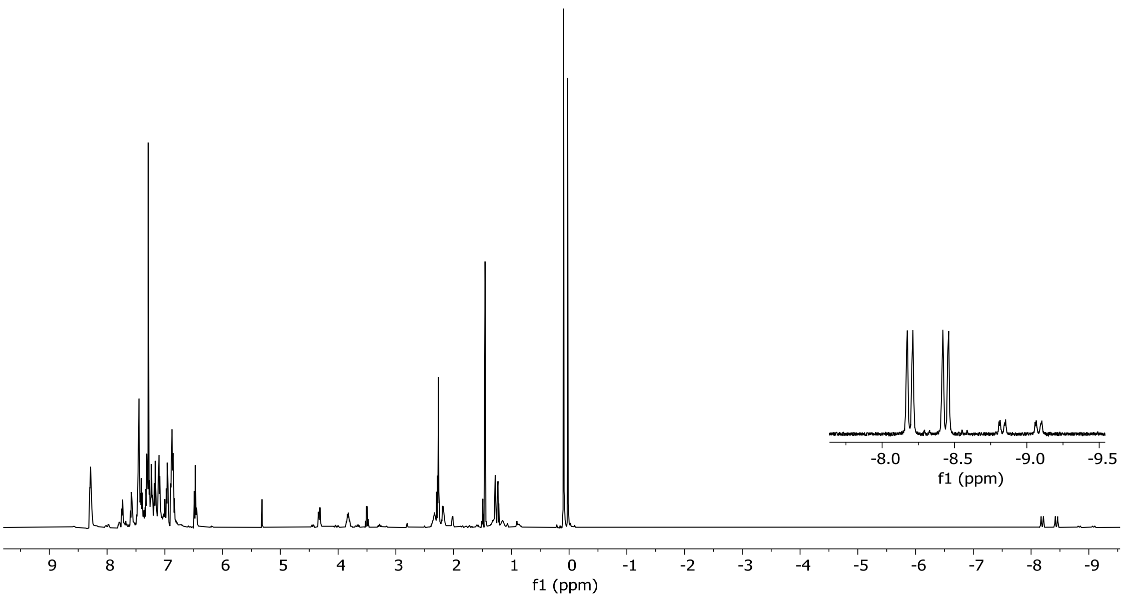

Figure S75 ^{1}H NMR of complex 5b in $CDCl_3$

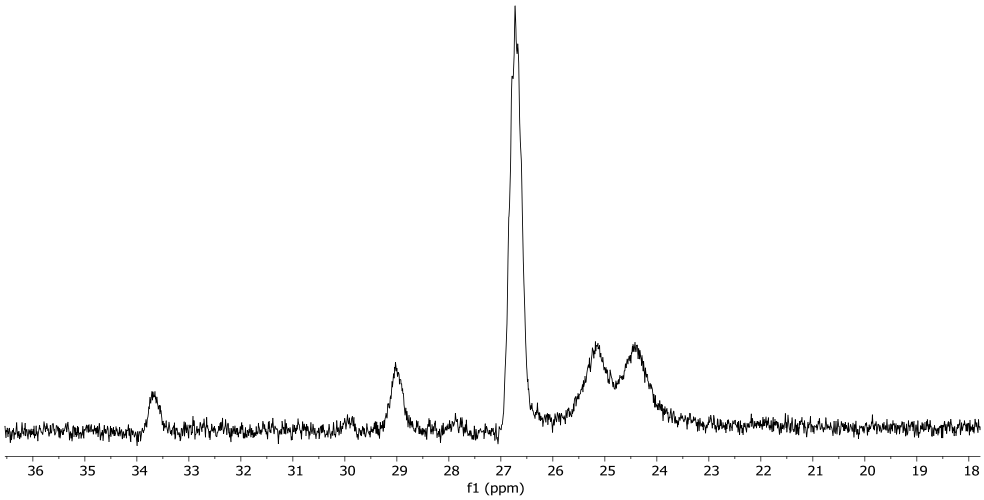

Figure S76 ^{31}P NMR of complex 5b in $CDCl_3$

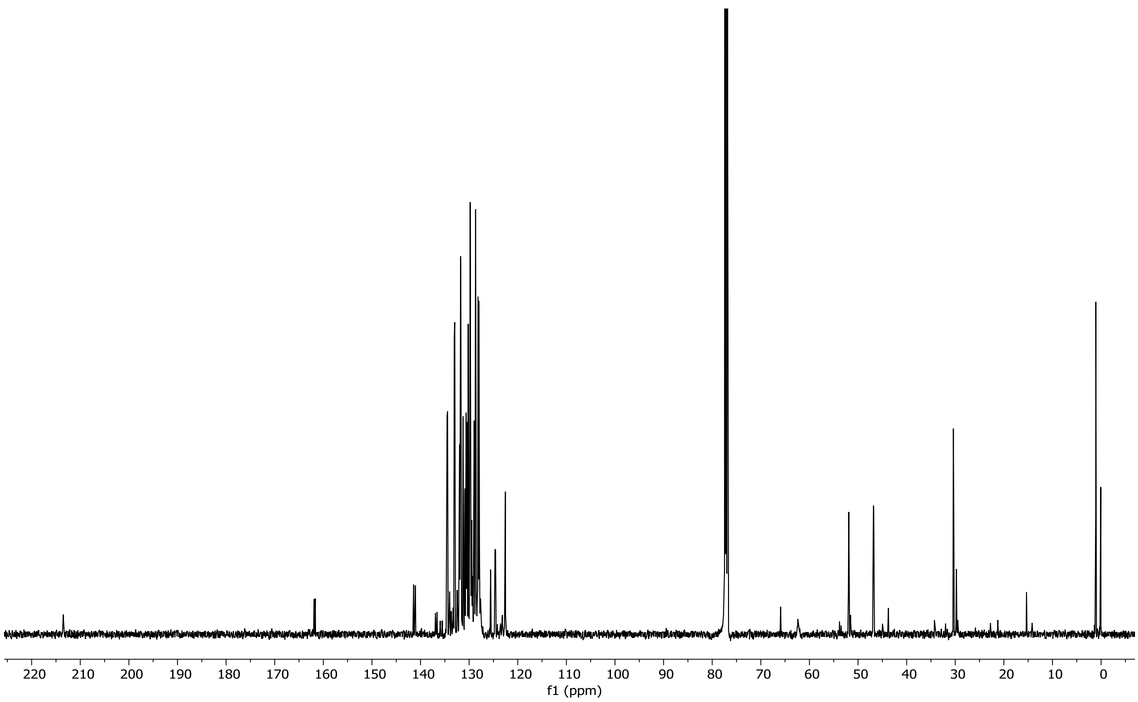

Figure S77 $^{13}C\{^{1}H\}$ NMR of complex 5b in $CDCl_3$

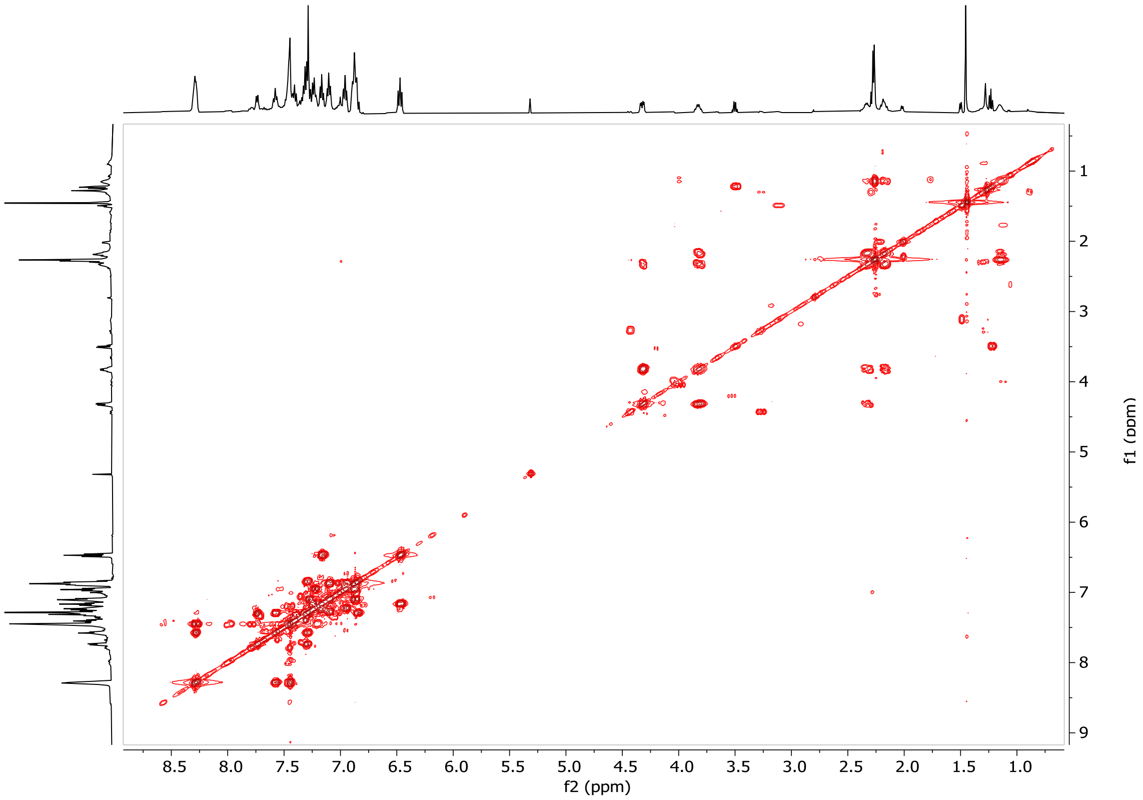

Figure S78 COSY spectrum of complex 5b in $CDCl_3$

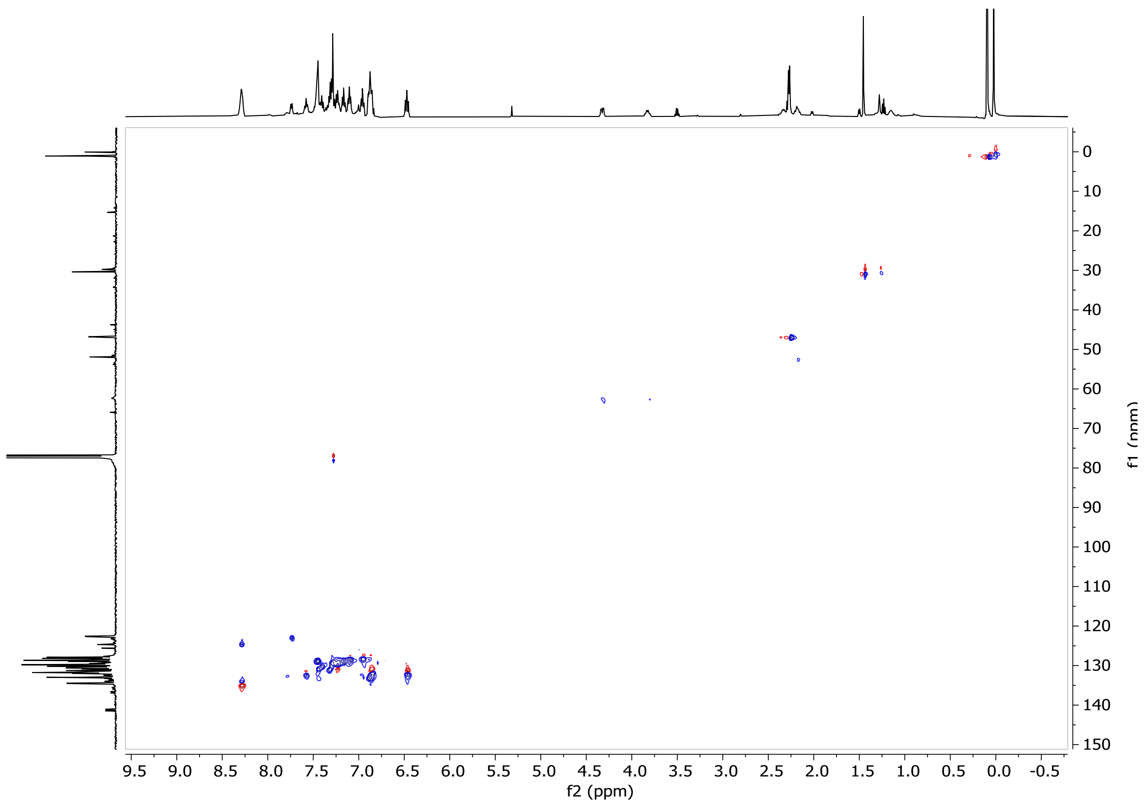

Figure S79 ^{1}H-^{13}C HSQC spectrum of complex 5b in $CDCl_3$

Compound 5d

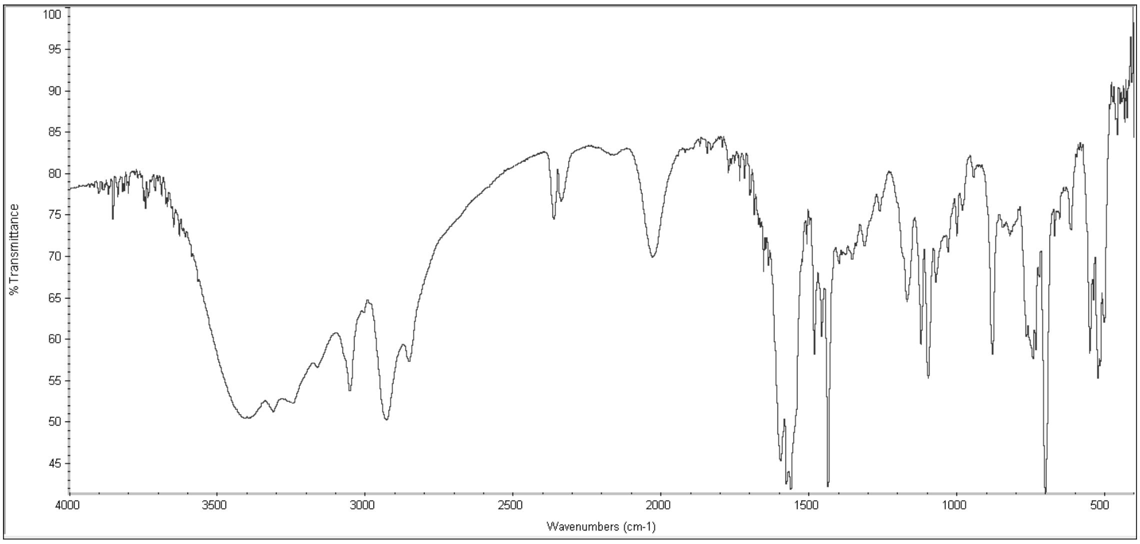

Figure S80 IR Spectrum of complex 5d

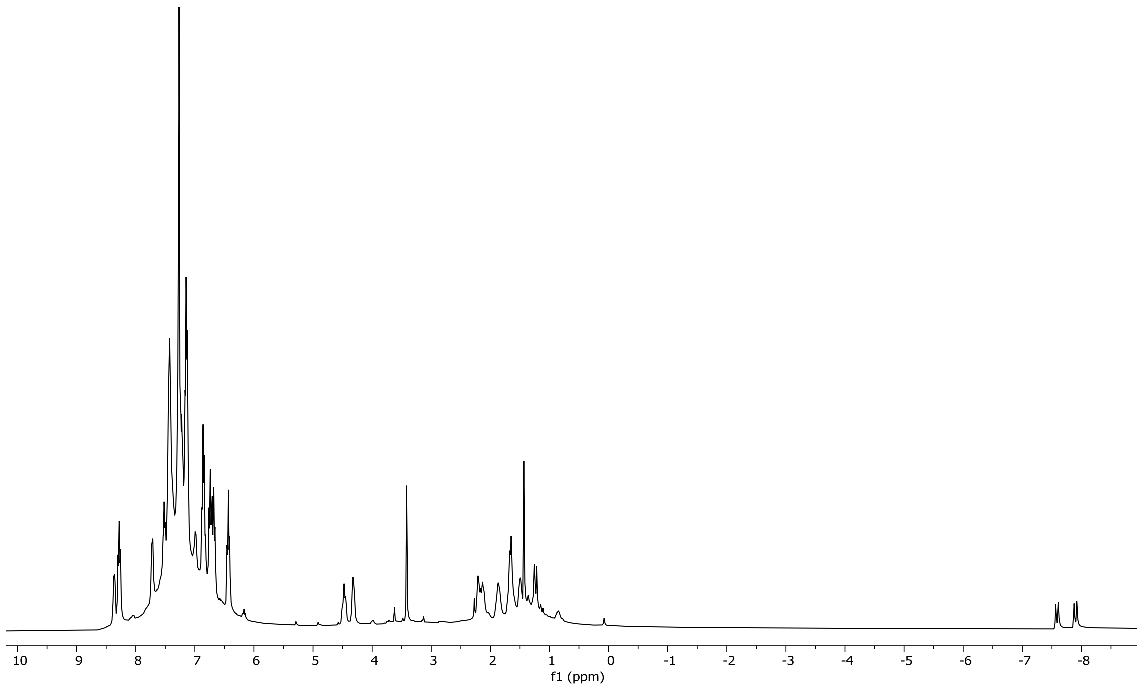

Figure S81 ^{1}H NMR of complex 5d in $CDCl_3$

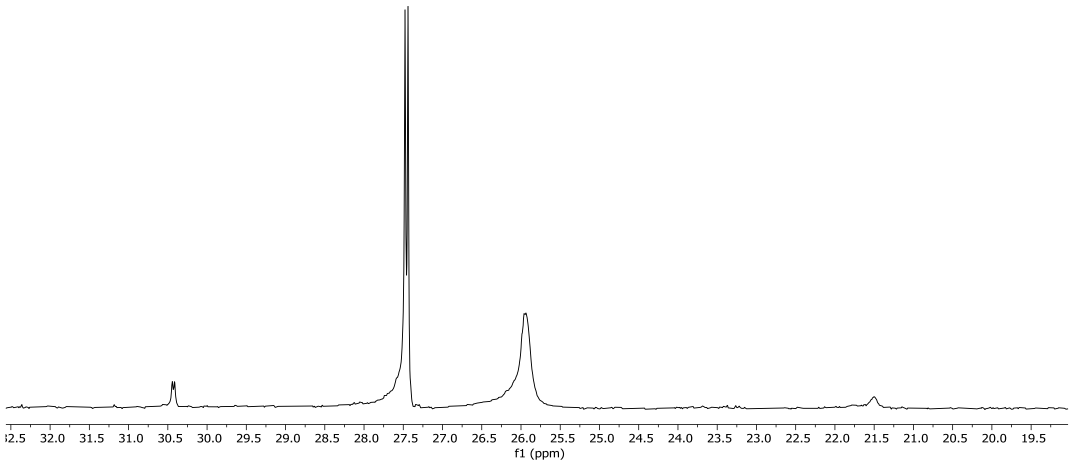

Figure S82 $^{31}P\{^{1}H\}$ NMR of complex 5d in $CDCl_3$

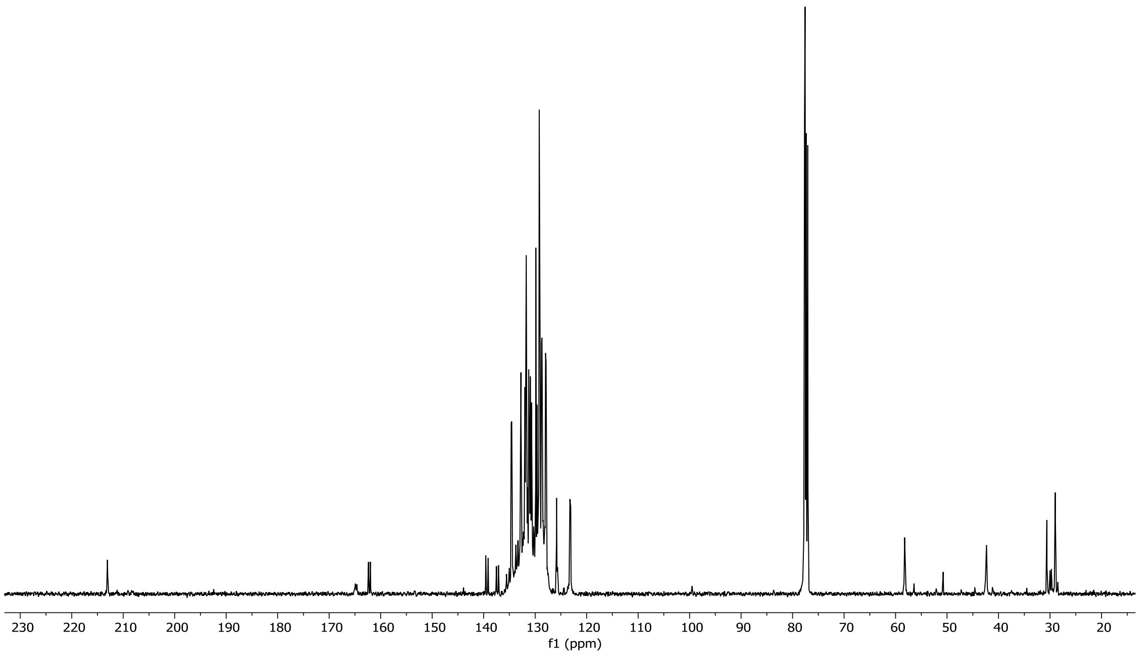

Figure S83 $^{13}C\{^{1}H\}$ NMR of complex 5d in $CDCl_3$

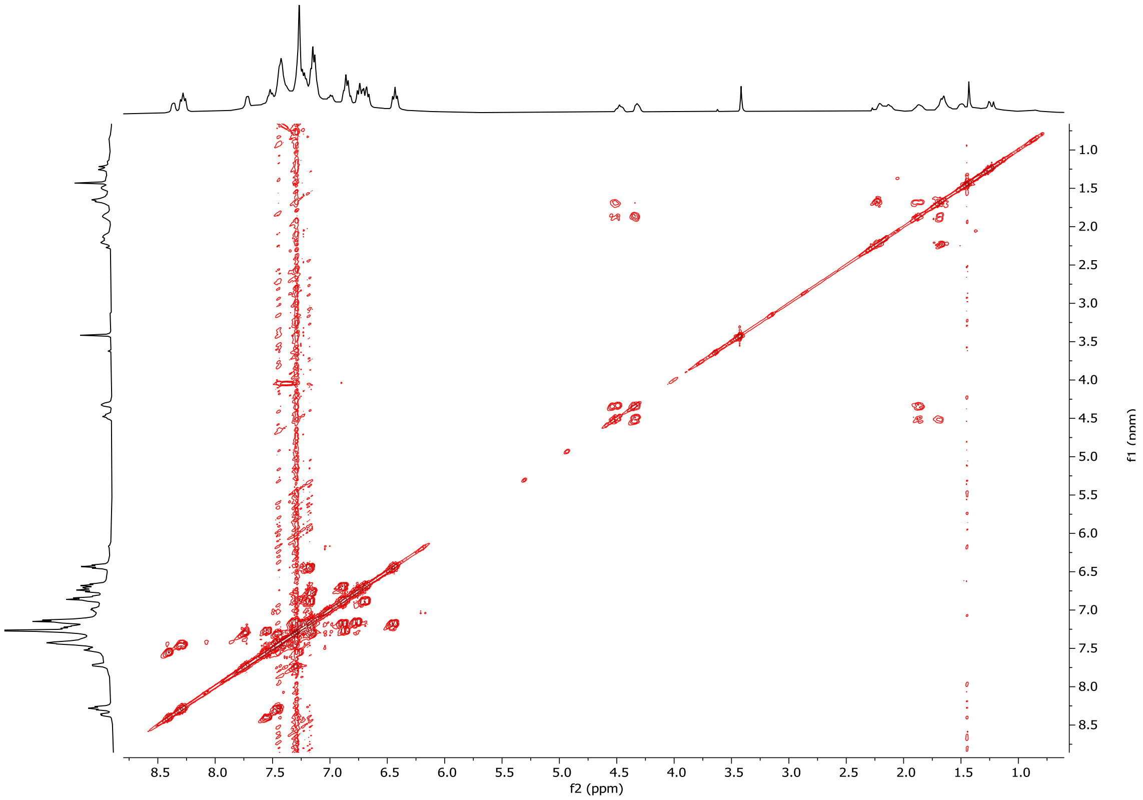

Figure S84 COSY spectrum of complex 5d in $CDCl_3$

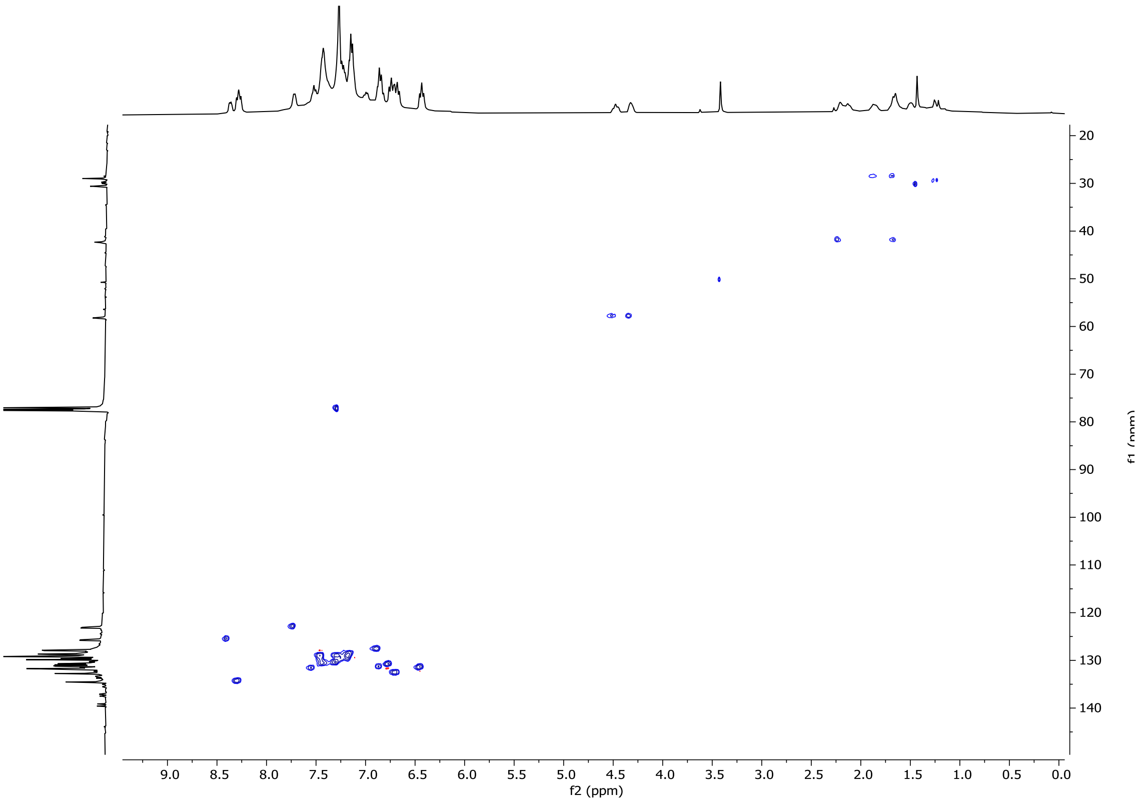

Figure S85 ^{1}H-^{13}C HSQC spectrum of complex 5d in $CDCl_3$

X-Ray diffraction data

Table S1 Diffraction data

**Compound**	**2a**	**3a**	**3b**	**4a**	**5b**
Formula	$C_{42}H_{44}N_2O_3ClP_2Ir$	$C_{47}H_{56}N_2O_3ClP_2Ir$	$C_{45}H_{42}N_2OCl_{13}P_2Ir$	$C_{44}H_{46}N_2O_6ClP_2Ir$	$C_{47}H_{49}N_2O_2Cl_6P_2Ir$
M_r	914.38	986.52	1341.79	988.42	1140.72
Crystal system	Triclinic	Triclinic	Triclinic	Monoclinic	Triclinic
Space group	*P-1*	*P-1*	*P-1*	*P21/c*	*P-1*
a (Å)	12.4725(6)	9.484(3)	10.4247(5)	11.5176(3)	10.9899(4)
b (Å)	12.5770(6)	13.765(3)	13.8608(7)	25.8417(6)	13.4251(5)
c (Å)	13.6087(6)	17.745(3)	20.5812(10)	13.9211(4)	18.1781(8)
a (°)	74.250(2)	112.577(3)	70.560(2)	90.00	72.676(2)
β (°)	70.8400(10)	95.277(3)	87.027(2)	91.942(1)	75.023(2)
γ (°)	89.034(2)	93.435(3)	74.011(2)	90.00	66.884(2)
V (Å^3)	1934.65(16)	2118.5(8)	2693(2)	4141.02(19)	2323.98(16)
Z	2	2	2	4	2
D_c (g cm^{-3})	1.570	1.547	1.655	1.585	1.630
m(MoK$_a$) (mm^{-1})	3.644	3.334	3.219	3.416	3.327
T (K)	100(2)	100(2)	100(2)	100(2)	100(2)
Observed reflections	10039	11027	7797	10676	12072
Parameters	460	506	577	507	548
F000	916	1000.0	1324.0	1984.0	1140.0
Tmin,Tmax	0.695, 0,746	0.621, 0.746	0.638, 0.745	0.636, 0.746	0667, 0.746
h,k,lmax	16, 17, 18	12,18, 24	11, 15, 22	15, 34, 18	14,18,24

Catalytic studies

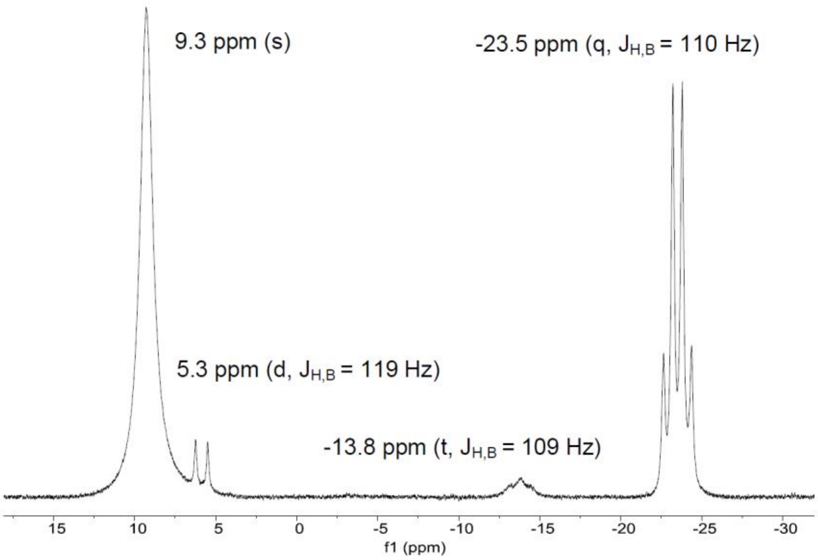

Figure S86 ^{11}B NMR of the *in situ* methanolysis of AB by complex 2a showing the different intermediates; NH_3BH_3 at -23.5 ppm, $NH_3BH_2(OCH_3)$ at -13.8 ppm, .$NH_3BH(OCH_3)_2$ at 5.3 ppm and $[B(OCH_3)_4]^-$ at 9.3 ppm.

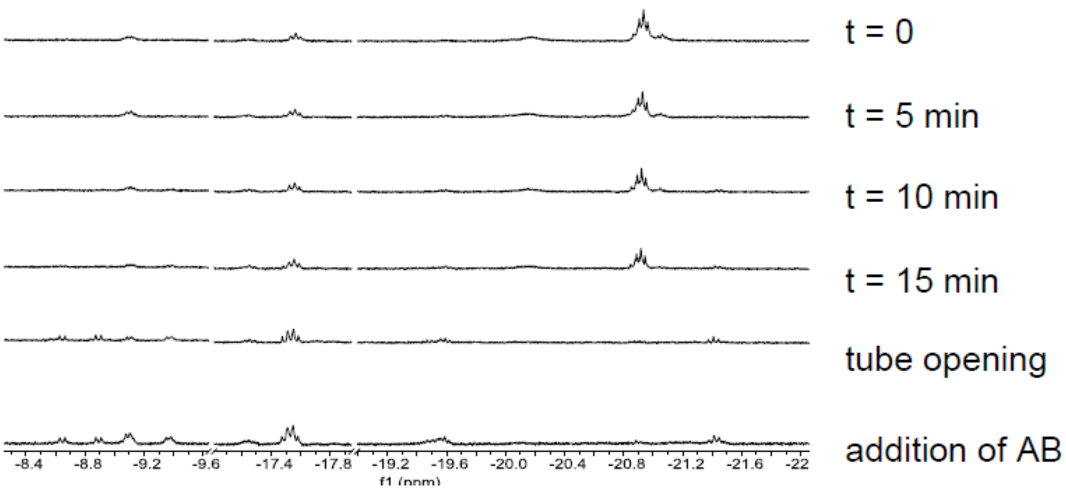

Figure S87 *In situ* ^{1}H NMR in the methanolysis of AB by 2a.

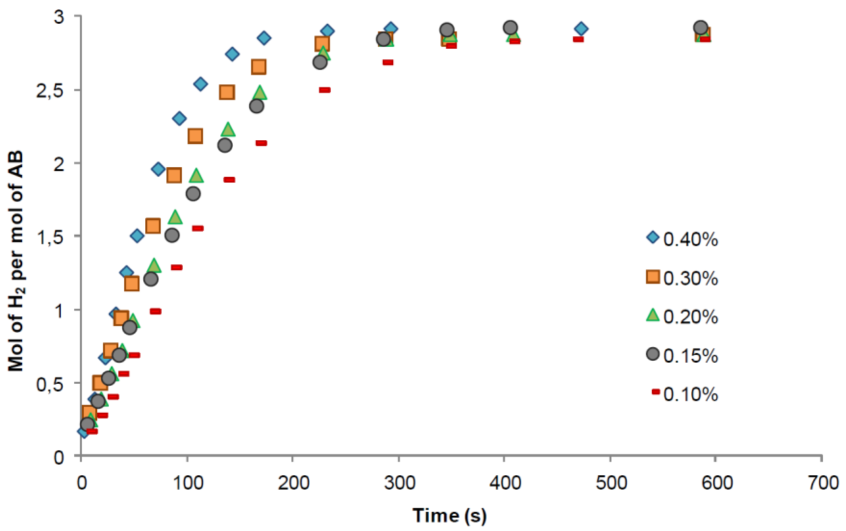

Figure S88 Hydrogen release from 0.46 M AB with various concentrations of catalyst 2d in 2.5 ml of MeOH.

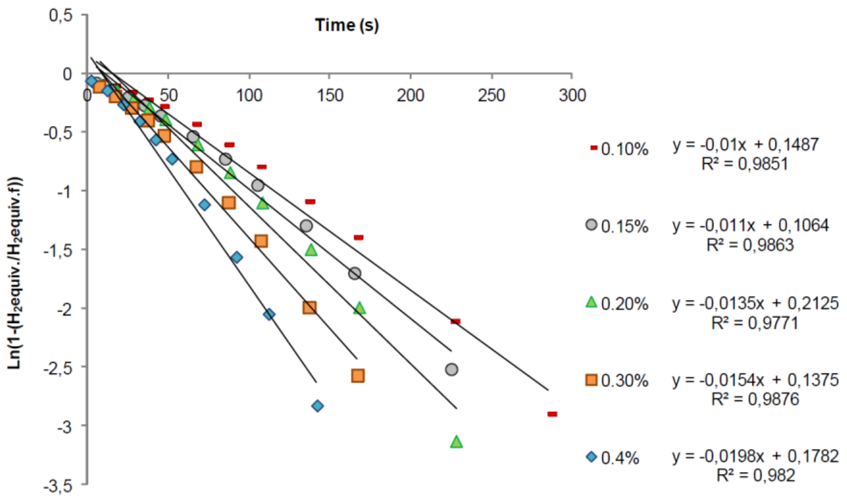

Figure S89 First order plots for the hydrogen release from 0.46 M AB with 2d in different concentrations at 60 °C.

Table S2 conversion, time required and rate constant for the methanolysis of 0.46 M of AB with different loadings of catalyst 2d.

Catalyst %	Conversion %	Time (s)	$10^3 \cdot k_{obs}$ (s^{-1})
0.10	95	480	10±0.4
0.15	97.7	420	11±0.4
0.20	96	360	13.5±0.7
0.30	96	300	15.4±0.6
0.40	97.3	240	19.8±0.9

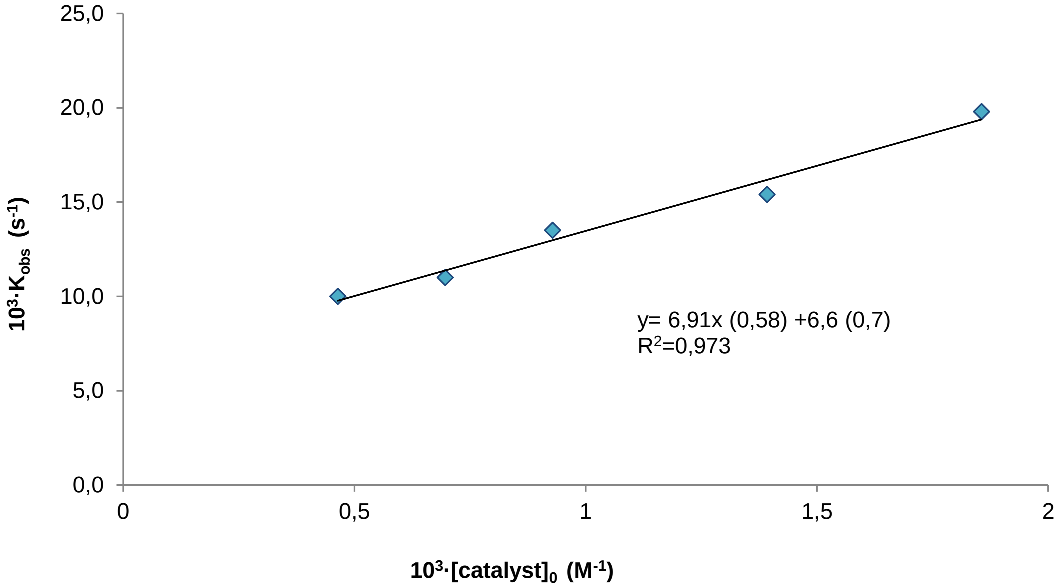

Figure S90 A plot of the pseudo-first-order rate constant (k_{obs}) versus $[catalyst]_0$ in the 1.86 × 10^{-3} to 0.46 × 10^{-3} M range for the catalyst 2d.
